# Alternative Growth Promoters Modulate Broiler Gut Microbiome and Enhance Body Weight Gain

**DOI:** 10.3389/fmicb.2017.02088

**Published:** 2017-10-26

**Authors:** Serajus Salaheen, Seon-Woo Kim, Bradd J. Haley, Jo Ann S. Van Kessel, Debabrata Biswas

**Affiliations:** ^1^Environmental Microbial and Food Safety Laboratory, Beltsville Agricultural Research Center, Agricultural Research Services, United States Department of Agriculture, Beltsville, MD, United States; ^2^Department of Animal and Avian Sciences, University of Maryland, College Park, MD, United States; ^3^Center for Food Safety and Security Systems, University of Maryland, College Park, MD, United States

**Keywords:** metagenomics, antibiotic growth promoters, antibiotic resistance genes, phenolics

## Abstract

Antibiotic growth promoters (AGPs) are frequently used to enhance weight-gain in poultry production. However, there has been increasing concern over the impact of AGP on the emergence of antibiotic resistance in zoonotic bacterial pathogens in the microbial community of the poultry gut. In this study, we adopted mass-spectrophotometric, phylogenetic, and shotgun-metagenomic approaches to evaluate bioactive phenolic extracts (BPE) from blueberry (*Vaccinium corymbosum*) and blackberry (*Rubus fruticosus*) pomaces as AGP alternatives in broilers. We conducted two trials with 100 Cobb-500 broiler chicks (in each trial) in four equal groups that were provided water with no supplementation, supplemented with AGP (tylosin, neomycin sulfate, bacitracin, erythromycin, and oxytetracycline), or supplemented with 0.1 g Gallic acid equivalent (GAE)/L or 1.0 g GAE/L (during the last 72 h before euthanasia) of BPE for 6 weeks. When compared with the control group (water only), the chickens supplemented with AGP and 0.1 g GAE/L of BPE gained 9.5 and 5.8% more body weight, respectively. The microbiomes of both the AGP- and BPE-treated chickens had higher Firmicutes to Bacteroidetes ratios. AGP supplementation appeared to be associated with higher relative abundance of bacteriophages and unique cecal resistomes compared with BPE supplementation or control. Functional characterization of cecal microbiomes revealed significant animal-to-animal variation in the relative abundance of genes involved in energy and carbohydrate metabolism. These findings established a baseline upon which mechanisms of plant-based performance enhancers in regulation of animal growth can be investigated. In addition, the data will aid in designing alternate strategies to improve animal growth performance and consequently production.

## Introduction

The discovery of antibiotics in the early 20th century followed by their economization due to large scale production during World War II for controlling human infections revolutionized food animal production, specifically poultry and swine, in the post war era (Jukes, 1977). Antibiotics used in agricultural animals account for more than half of the antibiotics produced in the United States (Lipsitch et al., 2002; Landers et al., 2012). Concerns over environmental and public health risks associated with the emergence of antibiotic resistance in zoonotic bacterial pathogens due to therapeutic and/or non-therapeutic use of antibiotics have led to global interest in adopting more stringent use of antibiotics in food animal production. In 1986 Sweden banned the use of antibiotic growth promoters (AGPs) and this was followed by a series of events that led to an EU-wide ban that took effect on January 1, 2006.^1^ Recently, the Center for Veterinary Medicine, Food and Drug Administration (FDA) recommended judicious use of medically important antibiotics in feed based on key reports and studies describing the impacts of AGP on development and transfer of antibiotic resistance traits among intestinal microbiota and to the environment and humans (Kuehn, 2014). In addition, the United States Department of Agriculture (USDA) established the National Organic Program which includes organic poultry production focusing on antibiotic free poultry production and good environmental practices.

Withdrawing sub-therapeutic use of antibiotics in conventional as well as organic poultry production systems may help to mitigate the emergence of antibiotic resistance in pathogens. However, reduced growth rates in animals that are observed in the absence of AGPs will impact the efficiency of production and perhaps jeopardize food security. These emerging issues for both conventional and organic poultry production highlight the need for alternative approaches to improve feed efficiency in the absence of AGP supplementation.

Byproducts (pomaces) from the berry juice industry: including blueberry (*Vaccinium corymbosum*) and blackberry (*Rubus fruticosus*), are major sources of phenolics that have roles in health improvement through anti-inflammatory, antimicrobial, anti-carcinogenic, anti-oxidant, and vasodilatory along with other beneficial properties (Boivin et al., 2007; Jepson and Craig, 2007; Tzounis et al., 2011; Salaheen et al., 2014). Dietary supplementation of plant phenolic extracts has been demonstrated to enhance growth performance in broilers but the mechanism of action has not yet been elucidated (Hernández et al., 2004). Recent observational and epidemiological studies indicated differences in the microbial communities of the ceca of conventional broilers to their organic counterparts (Torok et al., 2011; Singh et al., 2013; Mancabelli et al., 2016). Observed neutral effects of AGPs on germ free animals indicated the importance of AGP-dependent gut microbiota modulation on growth promotion in animals (Coates et al., 1963). Turnbaugh et al. (2006) revealed the association of two dominant bacterial phyla, the Bacteroidetes and the Firmicutes, with weight gain, an observation that was supported by other subsequent studies (Gong et al., 2008; Singh et al., 2013; Mancabelli et al., 2016). Gong et al. (2008) reported an increased abundance of *Lactobacillus* spp., Clostridiales and Enterobacteriaceae in chicks that were treated with AGP. Additionally, a correlation between the Firmicutes/Bacteroidetes (F/B) ratio, weight gain, and antibiotic treatment was reported by Singh et al. (2013). These findings inspired a comparative investigation on the effect of AGP versus phenolics on the modulation of broiler gut microbiota, resistome profile, functional enzymes involved in digestion, phage induction and other potential mechanisms behind the improved growth performance.

The aim of this study was to determine the composition of phenolic extracts from blueberry and blackberry pomaces and evaluate their functionality as an alternative intervention to promote the growth of broilers via modulation of the gut microbiota. We further aimed to study the cecal resistomes of these broilers which will provide important insights into the applicability of berry pomace phenolics to replace AGPs in poultry production.

## Materials and Methods

### Extracts Preparation and HPLC-Tandem Mass Spectrometry (LC-MS/MS) Analysis

Blueberry and blackberry pomace extracts were prepared according to the protocol previously described using commercial pomaces (powder form) that were kindly donated by Milne Fruit Products Inc., WA and stored at 4°C (Salaheen et al., 2014, 2016). Berry pomace extract (BPE) was comprised of blackberry and blueberry pomace extracts at 1:1 v/v ratio. Total phenolic content in each extract was determined spectrophotometrically (Singleton et al., 1999) and expressed as Gallic Acid Equivalent (GAE). The pH of the crude extracts varied from 3.5 to 4.5. Screening of phenolic compounds was performed using the HPLC-MS method described previously by Peng et al. (2015). Briefly, sample injections were 5 μL and separations were performed on an Agilent 1100 system (Agilent Technologies, Santa Clara, CA, United States) coupled to an Agilent MSD-TOF (time-of-flight) mass spectrometer. Reversed-phase liquid chromatography was used to separate the samples with a Waters Atlantis T3 column (3 μm, 150 × 2.1 mm i.d.) (Waters, Milford, MA, United States). A binary mobile phase consisting of solvent systems A and B was used in gradient elution where A was 0.1% formic acid (v/v) in ddH_2_O and B was 0.1% formic acid (v/v) in acetonitrile. The mobile phase flow rate was 0.3 mL/min. The linear gradient was as follows: time 0 – 1 min, 0% B; time 40 min, 90% B; time 41 min, 90% B; time 42 min, 0% B; time 52 min, 0% B. Following the separation, the column effluent was introduced by electrospray ionization (ESI) into the MSD-TOF. In this study, samples were assayed using negative mode ESI. Source parameters were: gas temperature = 350°C, gas flow = 9 L/min, nebulizer = 35 psi, fragmentor = 125 V, and capillary voltage = 3500 V. Data were acquired with a mass range of 75 – 1000 m/z. Mass accuracy was guaranteed by the continuous infusion of Agilent Reference Mass Solution (G1969-85001). Individual chromatographic peaks were identified using Agilent’s Mass Hunter Qualitative Analysis software (v. B.06). Compounds were identified using Agilent’s Mass Profiler Professional software (v. 13.1). Peaks in duplicate injections were aligned to account for instrumental drifts in retention time and mass. Compounds were retained only if they appeared in both duplicate samples. Compounds were annotated by querying Agilent’s METLIN human metabolite database, with a mass error criteria of <5 ppm.

### Diet Regimens and Weight Gain in Chick Model

Diet supplement experiments in chickens were carried out in duplicate trials. In each trial, 100 one-day-old Cobb-500 broiler chicks were obtained from Longenecker’s Hatchery Inc. (Elizabethtown, PA, United States). All procedures were approved by the Institutional Animal Care and Use Committee (IACUC, protocol number R-16-33) in accordance with established chick husbandry guidelines recommended by the aforementioned committee. The chicks were provided with commercially available crumbles (Purina Animal Nutrition, Gray Summit, MO, United States) with no antibiotic supplementation. The chicks were assigned into four groups of 25 chicks each in floor pens using a Completely Randomized Design consisting of a negative control, a positive control, and two treatment groups. The negative control group A was provided non-supplemented tap water; the positive control group B was provided tap water supplemented with AGP (a combination of Oxytetracycline 1 μg/mL, Erythromycin 2 μg/mL, Tylosin 2 μg/mL, Bacitracin 4 μg/mL and Neomycin sulfate 32 μg/mL); the treatment group C was provided tap water supplemented with 0.1 g GAE/L of BPE; and treatment group D was provided tap water supplemented with 0.1 g GAE/L of BPE and the treatment concentration was increased to 1 g GAE/L during the last 72 h before euthanasia. The chicks were reared for 6 weeks and individual weights was recorded weekly. The data were analyzed with the Statistical Analysis System software (SAS, Institute Inc., Cary, NC, United States) using mixed effect Analysis of Variance (ANOVA) with both group and trial as variables and Tukey’s modification for multiple mean comparisons.

### Sample Collection, Processing and Analysis

After 6 weeks, all the birds were euthanized with cervical dislocation followed by decapitation. Blood samples were collected in VACUETTE^®^ Heparin Tubes (Greiner Bio-One, Monroe, NC, United States) and were analyzed with a ProCyte Dx^®^ Hematology Analyzer (IDEXX, Westbrook, ME, United States) according to the manufacturer’s instructions. Chicken organs, e.g., spleen, liver, heart, and pancreas, were collected and weighed immediately. Ceca lengths were measured and contents from both ceca were thoroughly mixed followed by storage at -80°C until DNA extraction for metagenomic analysis. Cecal contents from 5 ceca per group (total 20) were randomly selected for metagenomic analysis. DNA extraction was carried out with QIAamp Fast DNA Stool Mini Kit (QIAGEN, Valencia, CA, United States) according to the manufacturer’s instructions. Nextera DNA libraries were made for each of the 20 samples separately using Nextera DNA Library Preparation Kit and Nextera Index Kit (Illumina, San Diego, CA, United States) followed by pooling into equimolar concentrations according to the manufacturer’s instructions. Paired-end sequencing (2 × 151 bp) was conducted on an Illumina NextSeq 500 sequencing platform with a NextSeq 500/550 v2 High Output flow cell. Metagenomic sequence data have been deposited at NCBI under accession numbers SRR5280289, SRR5280393, SRR5280514, SRR5280757, SRR5280758, SRR5280759, SRR5281813, SRR5281814, SRR5281815, SRR5282096, SRR5282097, and SRR5282098.

### Analysis of Metagenomic Datasets

Data were demultiplexed using the BCL2FastQ program and PhiX reads were removed using DeconSeq (Schmieder and Edwards, 2011). Reads were further cleaned using Trimmomatic V 0.36 (leading 20, trailing 20, sliding 4:20, min len 36) (Bolger et al., 2014). Only paired data were further analyzed. After cleaning and curating the data the total reads in each sample ranged from 4.3 × 10^7^ to 8.7 × 10^7^ reads. Taxonomic labels were assigned to reads with taxonomic sequence classifier, Kraken, using Kraken-translate and –mpa format which reported levels of the taxonomy with standard rank assignments (Wood and Salzberg, 2014). The Kraken database was prepared using NCBI taxonomic information as well as the complete genomes in RefSeq for the bacterial, archaeal, and viral domains^2^. Output files from Kraken were formatted with custom scripts to generate files containing taxonomic information and abundances in a tab delimited csv format which were loaded into MEGAN (version 5.11.3) (Huson et al., 2007) to generate matrices for sample comparisons. We determined relative abundances of microbial taxa in a cecum sample by dividing the number of reads of a specific taxon by the total number of reads in that sample. Initially the Bray-Curtis distance matrix and the Firmicutes/Bacteroidetes (F/B) ratio were determined for each sample in each treatment group (groups A, B, C, and D). Samples within a treatment group that clustered together and possessed an F/B value ranging from 0.10 to 10.0 (F/B is a gauge of overall gut microbiota balance and associated with AGP supplementation and weight gain in animals) were determined to be the core microbiome representing that group and these samples were selected for further analysis (total 12 samples, 3 from each group) (Mariat et al., 2009; Singh et al., 2013; Mancabelli et al., 2016). Relative abundances from MEGAN-generated abundance matrices were used for statistical analysis, calculation of alpha- and beta-diversity. Rarefaction curves with rarefied number of taxa, and alpha diversity indices were calculated using the vegan and phyloseq packages in R (version 3.3.1) (McMurdie and Holmes, 2013). Non-metric multi-dimensional scaling (NMDS) on Bray-Curtis distances were performed with vegan. Permutational ANOVA (PERMANOVAs) was carried out with the ‘adonis’ function in vegan.

For functional analysis, paired sequences were analyzed with metAMOS pipeline (Treangen et al., 2013). Output files from functional annotation section in metAMOS, which consisted UniProt ID of predicted proteins, were formatted with custom scripts to generate assigned UniProt ID and abundances in a tab delimited file. Retrieved UniProt entries were converted to the corresponding KEGG Orthology (KO) entries using Retrieve/ID mapping at http://www.uniprot.org/uploadlists/. A comparison matrix was prepared with Microsoft Query in Excel (version 2013) and fed to GraphPad Prism software (version 7) for statistical analysis. Interrogation of sequence reads for identity to known antibiotic resistance genes (ARGs) was performed using DIAMOND (Buchfink et al., 2015) (sequence identity ≥ 90%, matched amino acid sequence length ≥ 25, *e*-value ≤ 10^-5^) with the database CARD (McArthur et al., 2013), which encompasses amino acid sequences of ARGs.

## Results And Discussion

### Composite Analysis of BPE and Their Functional Roles

As rich sources of bioactive phenolics, blackberry and blueberry pomaces are plausible and economic raw materials for phenolics extraction and utilization in poultry production (Salaheen et al., 2016). In this study, we used 1:1 v/v mixture of blackberry and blueberry pomace extracts (BPE) as alternative to AGP in poultry production. The concentrations of the BPE stock solutions were adjusted to 6–8 g GAE/L which remained stable (no significant change in total phenolic contents) for up to 1 month at room temperature and 6 months at 4°C. HPLC-MS analysis of these crude extracts with negative ionization mode showed the presence of a wide array of components (Supplementary File 1). With the negative ionization mode in HPLC-MS, 5108 (2320 unique) and 4445 (2221 unique) compounds were detected in blackberry and blueberry pomace extracts, respectively (**Table 1**). Phenolic compounds in these extracts included apigenin, acetoxyeugenol, chlorogenic acid, cinnamic acid, coumarin, ellagic acid, flavan, flavanone, gallic acid, gingerol, glucuronides, hydroxydaidzein, myricetin, phenols, quercetin, quinones, rhamnosides, and xanthine. Wide structural variability in the phenolic derivatives was observed. The list of components identified using positive ionization mode was previously published (Salaheen et al., 2016).

**Table 1:** Number of compounds detected in blueberry and blackberry pomace extracts using MSD-TOF.

Source	Ionization mode	No. of compounds^a^	No. of unique compounds
Blueberry pomace extract	(+) ve	1103	605
Blueberry pomace extract	(-) ve	4445	2221
Blackberry pomace extract	(+) ve	1638	985
Blackberry pomace extract	(-) ve	5108	2320


^a^*Complete list of compounds are included in Supplementary File 1*.

**FIGURE 1:**
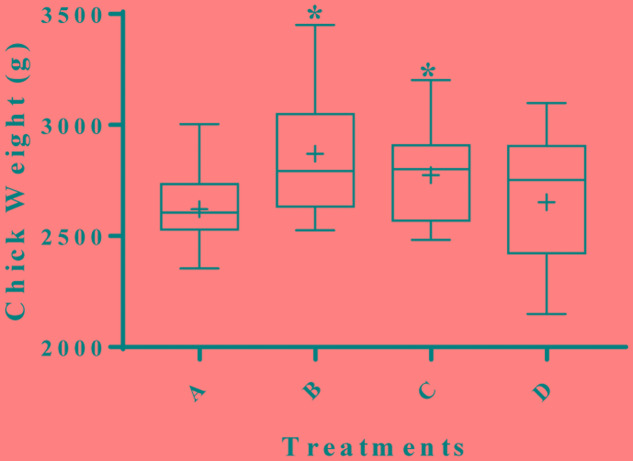
Effect of berry pomace extracts (BPE) compared to AGPs on the performance of broilers at day 42. Groups were assigned in the following manner; broilers from group A (negative control) were provided with: only tap water, group B (positive control): tap water with AGP, group C: tap water with 0.1 g GAE/L of BPE, and group D: tap water with 0.1 g GAE/L of BPE for 39 days and 1.0 g GAE/L of BPE for last 3 days before euthanasia. ^∗^ indicates significant variation compared to the negative control (*P* < 0.05).

The effect of supplementing water with BPE on chicken weight was measured in a total of 200 Cobb-500 broilers grown up to 6 weeks of age in duplicate trials (**Figure 1**). The body weight of group B chickens (supplemented with AGP) was 9.5% higher (2871 g) than the mean body weight of chickens from the control group A (2621 g) (*P* = 0.001). The mean weights of group C chickens (2774 g), that were given water supplemented with 0.1 g GAE/L of BPE was 5.8% higher compared to the control (*P* = 0.029). No significant differences in final mean weights were observed between group B and group C (*P* = 0.242). Chickens from group D, where BPE concentration was increased to 1.0 g GAE/L during the 72 h before euthanasia, had a mean body weight of more than 1% (2651 g) compared to the control (*P* = 0.488). However, in a separate study with similar conditions, when chickens were given water with 1.0 g GAE/L BPE for consecutive 6 weeks, mean body weight was 4% lower than the control (data not shown), which suggested a concentration dependent mechanism of BPE on chicken growth performance. Previously, supplementation of plant extracts was also reported to be associated with increased weight gain in broilers (Hernández et al., 2004; Zhang et al., 2009). The organ weight to whole body weight ratios were the same for spleen (*P* = 0.844), liver (*P* = 0.548), heart (*P* = 0.560), and pancreas (*P* = 0.599) among chickens from all four groups (**Table 2**). However, mean cecum length was > 12% greater when chickens were provided water supplemented with a higher concentration of BPE for the last 72 h (*P* = 0.037). When blood samples collected from five randomly selected chickens from each group were tested, no significant differences were observed in tested parameters at alpha value of 0.05 (**Table 3**). Unaltered hematologic characteristics in chickens provided non-supplemented water or water supplemented with BPE or AGP indicate the presence of non-systemic growth promotion mechanisms in chickens.

**Table 2:** Relative organ to body weights and cecum length of broilers at day 42.

Organs	A	B	C	D	*p*-value
Spleen^a^	0.13_a_	0.12_a_	0.12_a_	0.13_a_	0.844^c^
Liver^a^	2.18_a_	2.22_a_	2.07_a_	2.08_a_	0.548
Heart^a^	0.53_a_	0.53_a_	0.50_a_	0.53_a_	0.56
Pancreas^a^	0.86_a_	0.79_a_	0.79_a_	0.79_a_	0.599
Cecum^b^	17.24_a_	18.64_a_	19.15_b_	19.43_b_	0.037


^a^*Ratio, organ weight to total body weight at time of euthanasia*; ^b^*Length (cm)*. ^c^*Multiple mean conmarison with Tukey. Ratios with different subscript letters (a–d) in a row are significantly different (p < 0.05)*.

**Table 3:** Effect of BPE compared to AGPs on blood parameters and indices of broilers at day 42.

Blood parameters	A	B	C	D	*p*-value
RBC (×10^6^/μL)	2.5 ± 0.2^a^	2.8 ± 0.2	2.8 ± 0.1	2.7 ± 0.1	0.07^b^
WBC (×10^3^/ μL)	551.5 ± 212.6	644.2 ± 159.2	565.7 ± 100.3	774.6 ± 87.3	0.11
Lymphocyte (×10^3^/μL)	13.5 ± 2.6	17.5 ± 6.7	15.4 ± 6.1	17.8 ± 5.4	0.58
Monocyte (×10^3^/μL)	0.4 ± 0.4	0.5 ± 0.3	0.7 ± 0.4	0.8 ± 1.5	0.85
Hemoglobin (g/100 mL)	8.2 ± 0.5	8.8 ± 0.6	9.1 ± 0.4	8.8 ± 0.3	0.07
Hematocrit (%)	29.4 ± 2.1	30.8 ± 1.7	31.4 ± 1.5	30.7 ± 0.9	0.26


^a^*Values indicate Mean ± Standard deviation*; ^b^*Multiple mean comparison with Tukey*.

### Comparisons of Gut Microbial Communities

**FIGURE 2:**
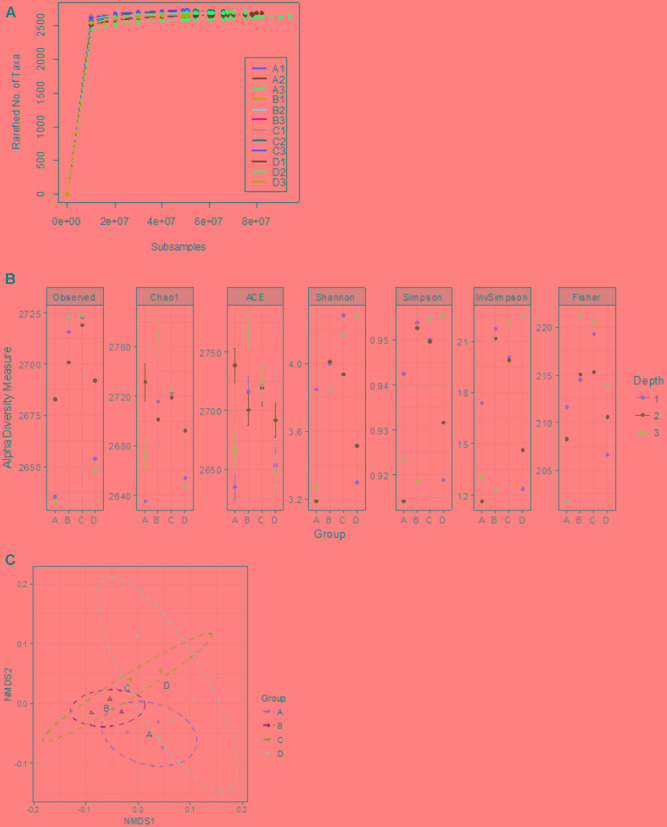
Assessment of alpha- and beta- diversity in samples from the control and treatment groups. **(A)** Displays the rarefaction curves indicating rarefied number of taxa at increasing sequencing depth of samples from groups A, B, C, and D. **(B)** Shows alpha-diversity measures of individual samples. **(C)** Exhibits non-metric multidimensional scaling (NMDS) plot of based on Bray-Curtis distance matrix encompassing 12 datasets from all the groups.

**FIGURE 3:**
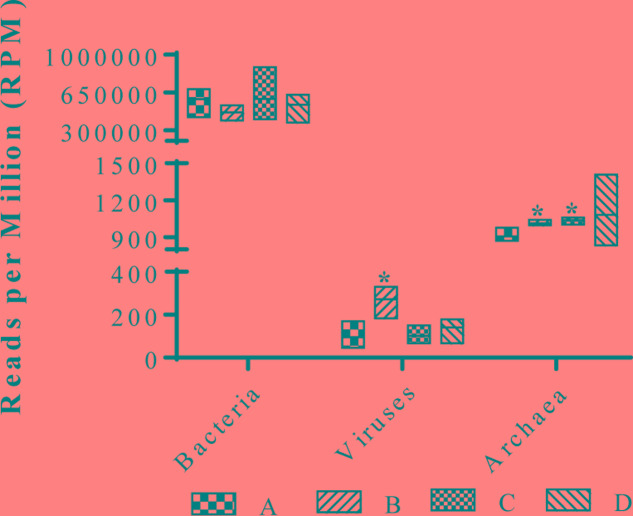
Relative abundances of various microbial taxa in broiler cecum from groups A, B, C, and D. ^∗^indicates significant difference in a group compared to negative control (group A) at *P* < 0.05.

To assess the composition of gut microbiota in the four treatment groups, we analyzed randomly selected cecal samples from 12 chickens (three chickens from each group) with next-generation shotgun sequencing. The total number of reads in the cleaned and curated datasets from each sample ranged from 4.3 × 10^7^ to 8.7 × 10^7^. Evaluation of rarefaction curves based on rarefied number of taxa (alternatively Operational Taxonomic Units) on sample datasets indicated that all 12 curves tend to form a plateau which bear evidence of adequate sequence coverage for the majority of biodiversity contained within the samples (**Figure 2A**). Rarefaction curves did not reveal a noticeable difference among bacterial taxa composition in the cecal contents from 12 chickens. However, higher alpha-diversity index values (Chao1, ACE, Shannon, Simpson, InvSimpson, and Fisher) were noticed in groups B and C, compared to the control (group A) (**Figure 2B**). The NMDS plot and PERMANOVA analysis on the Bray-Curtis distances among the microbial taxa revealed similarity between microbial communities in groups B and C, though statistical significance (at an alpha of 0.05) was not detected in this relationship (PERMANOVA *F* = 2.11, *R*^2^= 0.44, *P* = 0.095) (**Figure 2C**). Kruskal–Wallis rank sum test on relative abundances of microbial domains revealed that group B included a higher number of DNA viruses compared to the other groups (χ^2^ 7.21, *P* = 0.06) whereas no significant differences in the number of bacteria were observed among all the groups (**Figure 3**). A numeric reduction in the mean relative abundance of bacteria was noticed in group B compared to the other groups (χ^2^ 1.67, *P* = 0.64). Unpaired *t*-test analysis on relative abundance of Archaea demonstrated significantly higher numbers in groups B and C compared to group A (*P* = 0.049 and 0.031). Investigation into the biological significance of increased archaeal population in the microbiomes from groups B and C revealed that influence of archaeal communities in poultry gut is still an understudied area. It is known that conversion of organic compounds to simple volatile fatty acids by fermentative bacteria leads growth inhibition due to H_2_ accumulation in the gut. Methanogenic archaea in the GI tract consume H_2_ and help to lower H_2_ partial pressure in gut (Saengkerdsub and Ricke, 2014).

**FIGURE 4:**
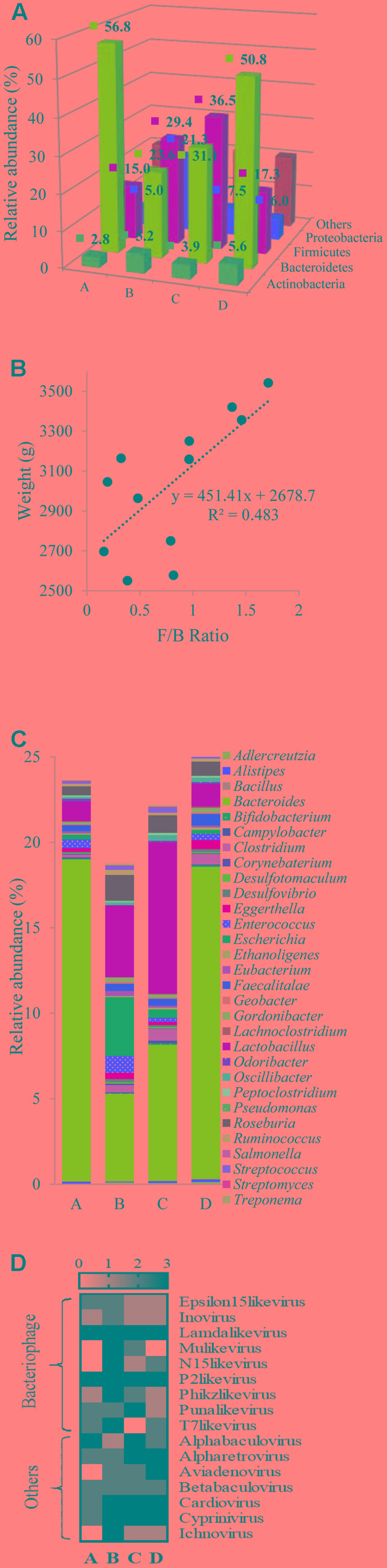
Differential composition of chicken cecal microbiota. **(A)** Depicts bacterial distribution at phylum level in 3D plot with pooled datasets. **(B)** Demonstrates a scatter plot of Firmicutes to Bacteoirdetes (F/B) ratio to broiler weight at 42 days of age. **(C)** Shows bacterial distribution at genus level in all the broiler groups. Finally, **(D)** displays the variation in the presence or absence of DNA viruses at genus level in samples from groups A, B, C, and D.

Assigned taxonomic profiles at the bacterial phylum level for pooled cecal samples revealed that Bacteroidetes was the dominant phylum (56.8%) in group A followed by Firmicutes (15%) and Proteobacteria (5%) (**Figure 4A**). Relative abundances of bacterial phyla in individual samples are presented in Supplementary File 2. In group A, relative abundances of Bacteroidetes, Firmicutes, and Proteobacteria ranged between 44.6–64.3%, 10.3– 21.5%, and 5.0–9.1%, respectively (Supplementary File 2). In groups B and C, bacterial phyla distribution was dominated by Firmicutes (29.45 and 36.46%) followed by Bacteroidetes (23.62 and 31.11%) and Proteobacteria (21.29 and 7.55%). Differences in the community composition were observed among the different treatment groups. A higher relative abundance of Bacteroidetes in group A (χ^2^ 7.20, *P* = 0.06) was detected, whereas a higher abundance of Firmicutes in groups B and C was observed compared to the other groups (χ^2^ 8.74, *P* = 0.03). Group B possessed highest relative abundance of Proteobacteria (χ^2^ 5.05, *P* = 0.17). This finding was supported by Looft et al. (2012) who also reported that non-therapeutic concentration of chlortetracycline, sulfamethazine, and penicillin administered to piglets increased the prevalence of Proteobacteria. It is important to note that Proteobacteria include a wide variety of human pathogens, such as *Escherichia*, *Campylobacter*, *Salmonella*, *Helicobacter*, *Pseudomonas*, and many other notable pathogenic genera.

Significant variation in the mean Firmicutes to Bacteroidetes (F/B) ratio (0.32, 1.32, 1.09, and 0.46 in groups A, B, C, and D, respectively) was observed (*P* = 0.01). Spearman correlation analysis indicated a positive correlation between F/B ratio to chicken body weight (Spearman ρ = 0.70, *P* = 0.01) (**Figure 4B**). This finding is supported by previous studies that showed increased F/B ratios were associated with AGP supplementation in feed and growth promotion in broilers (Singh et al., 2013; Mancabelli et al., 2016). In previous *in vitro* co-culture studies conducted in our lab, growth stimulation in Firmicutes, specifically probiotic *Lactobacillus* strains was detected in the presence of berry extracts in broth, or chicken fecal medium and addition of berry extracts resulted in a selective bias toward probiotic population when co-cultured with pathogens (Yang et al., 2014; Salaheen et al., 2015). These findings indicate that an increased Firmicutes level in chicken ceca might be one of the many factors responsible for growth promotion in chickens with BPE supplementation.

At the genus level a total of 670, 678, 691, and 683 bacterial taxa were identified in groups A, B, C, and D, respectively. As the reads were mapped to the lowest common ancestor (LCA) in this study, the identified genera in the broiler ceca may not be comprehensive. Mancabelli et al. (2016) identified 252 taxa at the genus level in broilers with 16s rRNA profiling. Sequencing methods, source of samples, and diet regimens may be responsible for variability in the number of identified bacterial genera in broilers. Noticeable differences in the relative abundances of *Bacteroides*, *Enterococcus*, *Escherichia*, and *Eubacterium* genera were identified among treatment groups where relative abundance of *Bacteroides* was the highest in group A; *Enterococcus* and *Escherichia* in group B; and *Lactobacillus* and *Eubacterium* in group C (**Figure 4C**; Supplementary File 3). Oakley and Kogut (2016) also reported significantly higher abundance of *Bacteroides* in ceca of broilers grown without AGP. Whereas, Viveros et al. (2011) reported higher abundance of *Lactobacillus* in broilers provided with plant extracts. Observed differences in the composition of bacterial genera may influence the dynamics of feed to energy conversion in chickens from different treatment groups.

There were no significant differences in the relative abundances of DNA viruses in the samples. However, based on presence or absence of a virus in a sample, bacteriophages e.g., Mu-like viruses, N15-like viruses, Phikz-like viruses, T7-like viruses, and Inoviruses were more prevalent in group B (**Figure 4D**). In addition, a numerical (but not statistically significant) increase in the mean abundances of Lamda-like viruses, Mardiviruses, and T4-like viruses was noticed in group B. Metagenomic studies in swine and mice models reported phage induction in the gut due to oral supplementation with antibiotics (Allen et al., 2011; Modi et al., 2013). A collateral consequence of phage induction is gene transfer that promotes both pathogen evolution and transfer of ARGs (Allen and Stanton, 2014).

### Functional Classification of Chicken Cecal Microbiome

**FIGURE 5:**
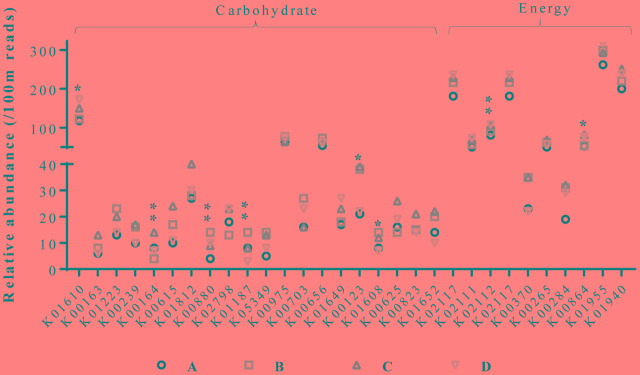
Functional classification of chicken cecal microbiomes. Relative abundances were calculated based on the numbers for every 100 million reads. ^∗^ and ^∗∗^ indicates significant difference in relative abundance of a KO functional orthog at alpha value of 0.15 and 0.10, respectively.

Assessment of the functional classification of open reading frames based on the KEGG Orthology (KO) database obtained from metagenomic datasets revealed significant variation in functional orthologs among the groups (**Figure 5**). Analysis was directed toward enzymes involved in carbohydrate metabolism and energy production and further sorted to enzymes showing variation among different datasets. Significant variations were observed in the relative abundances of several enzymes. Phosphoenolpyruvate carboxykinase, formate dehydrogenase major subunit, and 2-oxoglutarate dehydrogenase E1 component (involved in carbon metabolism, glyoxylate and dicarboxylate metabolism, methane metabolism, tryptophan metabolism, and lysine degradation) were more abundant in group C than in the other groups. Whereas, L-xylulokinase, α-glucosidase, and tartronate-semialdehyde synthase (involved in pentose and glucuronate interconversions, ascorbate, aldarate, starch, sucrose, glyoxylate, and dicarboxylate metabolism) were highly abundant in group B. Chicken cecal microbiome from group D possessed higher relative abundance of F-type H+-transporting ATPase subunit beta, and glycerol kinase. These observations indicate metabolic diversions in chickens that were provided with AGPs or BPE, though both of these treatments resulted in higher body weight in chickens. These differences can be explained by the variability in the microbial communities, especially, Bacteroidetes and Firmicutes, that harbor important enzymes (e.g., pectinase, and cellulase) to hydrolyze cell wall components from plant based diets (Thomas et al., 2011).

### Chicken Cecal Resistomes

**FIGURE 6:**
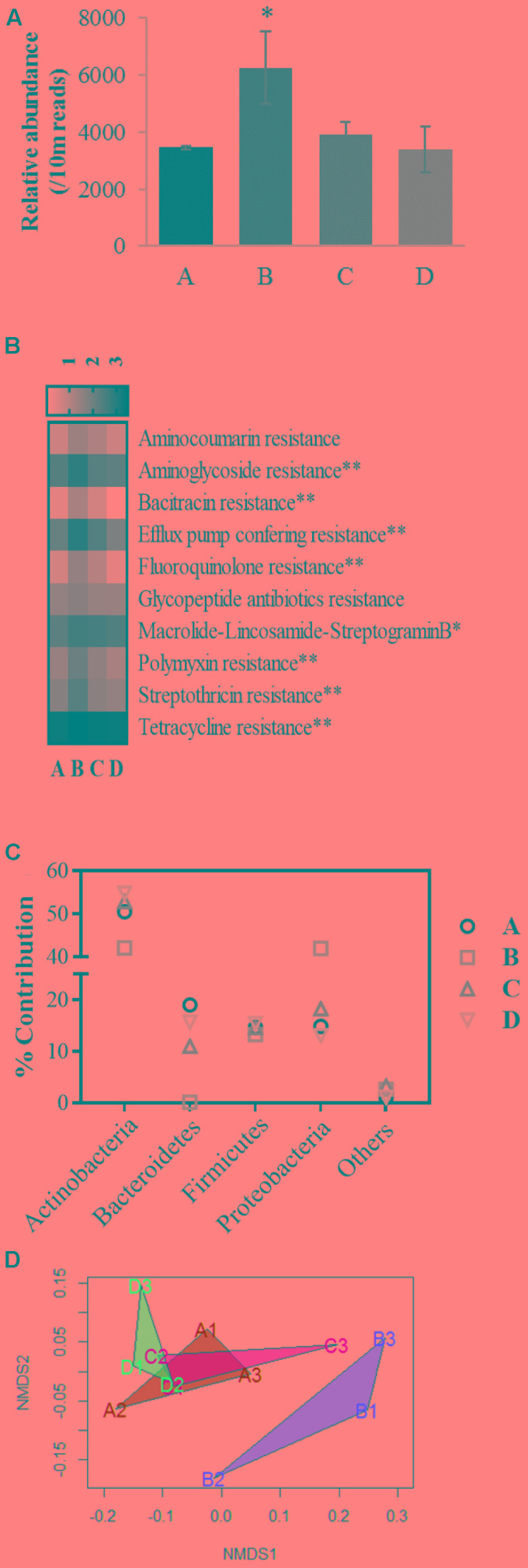
Evaluation of resistome profiles in chicken gut microbiome. **(A)** Shows relative abundance (reads per 10 million sequences) of total Antibiotic Resistance Genes (ARGs) like reads observed in resistomes of broiler ceca from groups A, B, C, and D. **(B)** Exhibits a heatmap encompassing relative abundances of shared ARGs among the treatment groups in logarithmic scale where ^∗∗^ and ^∗^ indicates significant difference at alpha value of 0.10 and 0.15, respectively. **(C)** Depicts percent contribution of microbial taxa on relative abundances of ARGs in the core resistomes. **(D)** Shows non-metric multidimensional scaling (NMDS) plot of Bray-Curtis distances of the chicken cecal resistomes.

The core resistome of the microbial consortia residing in chicken cecal contents collected from various groups were generated by screening for known bacterial ARGs followed by sorting the genes based on their relative abundances (at-least one read per 10 million sequences) in the cecal samples. *In silico* analysis of the resistome profiles demonstrated a higher relative abundance of ARG like reads in group B compared to group A (χ^2^ 8.13, *P* = 0.04) (**Figure 6A**). Core resistomes of groups A, B, C, and D consisted 69, 103, 88, and 69 unique ARGs that represented 3286, 5870, 3685, and 3081 reads per 10 million sequences, respectively. Both transferable ARGs and efflux pump mediatory ARGs were present in these core resistomes (Supplementary File 4). Core resistomes of the treatment groups were generated in the following way: 112, 79, and 103 ARGs in the resistomes from group A; 129, 124, and 121 ARGs in group B; 108, 102, and 115 ARGs in group C; and finally 94, 111, and 80 ARGs in group D. Based on gene functions, the identified ARGs in individual samples were further assigned to 14 groups: resistance to aminocoumarin, aminoglycosides, beta-lactam, sulfonamides, bacitracin, chloramphenicol, fluoroquinolone (transferable element based), glycopeptide antibiotics, trimethoprim, macrolide-lincosamide-streptogramin B, polymyxin, streptothricin, tetracyclines, and efflux pump conferring resistance. Chloramphenicol acetyltransferase (*cat*) and *CcrA* beta-lactamase genes were found in all the samples but due to assigned abundance threshold, i.e., at-least one read per 10 million sequences, these genes were not listed in core resistome of group B. Higher relative abundance of beta-lactamases were previously reported in broilers grown with AGPs but absence of beta-lactam antibiotics in the AGP-mixture that was used in this study may be responsible for lower abundance of beta-lactamases (Laube et al., 2013; Mancabelli et al., 2016). Two out of three samples from group B contained the trimethoprim resistance gene, *dfrA1*, hence not included in the group B resistome but no trimethoprim resistance gene was identified in other groups. Sulfonamide resistant dihydropteroate synthase genes (*sul1* and *sul2*) were only observed in all the samples from group B but not in other groups. The genes *sul1* and *sul2* are commonly associated with mobile genetic elements and predominance of resistant enteric bacteria associated with these genes has been reported in broilers grown with AGPs (Diarra et al., 2007; Simmons et al., 2016). In this study, percentages of ARGs associated with efflux pump mediated antibiotic resistance in core resistomes of groups A, B, C, and D were 39.1, 49.5, 46.6, and 39.1, respectively.

A total of 52 ARGs from 10 out of 14 ARG groups showed coexistence in all of the core resistomes. Relative abundances of aminoglycoside, bacitracin, fluoroquinolone (transferable elements), polymyxin, streptothricin, tetracycline and efflux pump conferring ARGs were higher in group B compared to the other groups (χ^2^ 6.44–7.7, *P* < 0.10) (**Figure 6B**). Relative abundances of macrolide-lincosamaide-streptogramin resistance genes were also numerically higher in group B (χ^2^ 5.82, *P* = 0.12). No significant variation in the relative abundances of aminocoumarin and glycopeptide ARGs were observed. In this study, incorporation of bacitracin (glycopeptide), erythromycin (macrolide), neomycin sulfate (aminoglycoside), oxytetracycline (tetracycline), and tylosin (macrolide), supplementation in chickens from group B is associated with higher relative abundances of ARGs in the cecal resistome. These findings are supported by previous studies that reported an association between the uses of antibiotics in feed with development of ARGs in complex ecosystems (Andersson and Hughes, 2014; Levy, 2014; Roca et al., 2015).

Based on the best-hit results from the DIAMOND analysis using the CARD database, the majority of identified ARGs were highly similar to those previously identified in Actinobacteria, Bacteroidetes, Firmicutes, and Proteobacteria (**Figure 6C**). Relative abundance of ARGs associated with Actinobacteria, more specifically Bifidobacteriales order ranged from 42.07 to 54.83% in the core resistomes. Significantly lower ARGs associated with *Bacteroidetes* were identified in group B (0.14%) compared to groups A, C, and D (19.03, 11.01, and 15.64%, respectively) which can be explained by reduced relative abundance of Bacteroidetes in cecal microbiome of group B. Mancabelli et al. (2016) also observed high abundance of Bacteroidetes-associated ARGs in cecal resistomes of chickens grown without antibiotic supplementation. Alternatively, higher relative abundances of ARGs associated with Proteobacteria were observed in group B (41.93%) compared to the other groups. Higher relative abundances of Proteobacteria in chicken cecal microbiomes from group B were likely to be responsible for higher Proteobacteria-associated ARGs (Danzeisen et al., 2011). The NMDS plot and PERMANOVA analysis on the Bray-Curtis distances among the cecal resistomes revealed significant variation in the cecal resistomes from group B compared to the other groups (PERMANOVA *F* = 7.77, *R*^2^= 0.74, *P* = 0.004), while resistomes from groups A, C, and D clustered together which indicate that BPE supplementation may be safe in terms of development and transfer of ARGs among microbial communities (**Figure 6D**).

To conclude, in this study we discovered BPE supplementation in water increased the mean body weight of chickens by 5.8% and caused an AGP-like pattern in the cecal bacterial community with a comparative increase of Firmicutes and a concomitant reduction of Bacteroidetes in chicken ceca. Findings from this study suggest that the use of bioactive phenolics from berry by-products as an AGPs alternative that will potentially play significant roles to mitigate the development of antibiotic resistance among zoonotic bacterial pathogens. However, further investigations at the farm level are needed to confirm our findings and evaluate its practicality through cost-benefit analysis.

## Author Contributions

SS, DB, and JVK contributed to design of the work. SS, S-WK, and DB contributed by performing experiments. SS, BH, S-WK, DB, and JVK contributed by analysis and interpretation of data for the work, responsible for the integrity of the work, drafting the manuscript, and revising the work. DB contributed to the conception and design of the work, ensuring that questions related to the accuracy or integrity of any part of the work are appropriately investigated and resolved, critically revising the final approval of the version to be published, and responsibility for the integrity of the work.

## Conflict of Interest Statement

The authors declare that the research was conducted in the absence of any commercial or financial relationships that could be construed as a potential conflict of interest.

## Footnotes

1 http://europa.eu/rapid/press-release_IP-05-1687_en.htm

2 https://www.ncbi.nlm.nih.gov/refseq/

## References

[B1] Allen H. K. Looft T. Bayles D. O. Humphrey S. Levine U. Y. Alt D. (2011). Antibiotics in feed induce prophages in swine fecal microbiomes. *mBio* 2:e00260-11. 10.1128/mBio.00260-11 22128350 PMC3225969

[B2] Allen H. K. Stanton T. B. (2014). Altered egos: antibiotic effects on food animal microbiomes. *Annu. Rev. Microbiol.* 68 297–315. 10.1146/annurev-micro-091213-113052 25002091

[B3] Andersson D. I. Hughes D. (2014). Microbiological effects of sublethal levels of antibiotics. *Nat. Rev. Microbiol.* 12 465–478. 10.1038/nrmicro3270 24861036

[B4] Boivin J. Bunting L. Collins J. A. Nygren K. G. (2007). International estimates of infertility prevalence and treatment-seeking: potential need and demand for infertility medical care. *Hum. Reprod.* 22 1506–1512. 10.1093/humrep/dem046 17376819

[B5] Bolger A. M. Lohse M. Usadel B. (2014). Trimmomatic: a flexible trimmer for Illumina sequence data. *Bioinformatics* 30 2114–2120. 10.1093/bioinformatics/btu170 24695404 PMC4103590

[B6] Buchfink B. Xie C. Huson D. H. (2015). Fast and sensitive protein alignment using DIAMOND. *Nat. Methods* 12 59–60. 10.1038/nmeth.3176 25402007

[B7] Coates M. E. Fuller R. Harrison G. F. Lev M. Suffolk S. F. (1963). A comparison of the growth of chicks in the Gustafsson germ-free apparatus and in a conventional environment, with and without dietary supplements of penicillin. *Br. J. Nutr.* 17 141–150. 10.1079/BJN19630015 14021819

[B8] Danzeisen J. L. Kim H. B. Isaacson R. E. Tu Z. J. Johnson T. J. (2011). Modulations of the chicken cecal microbiome and metagenome in response to anticoccidial and growth promoter treatment. *PLOS ONE* 6:e27949. 10.1371/journal.pone.0027949 22114729 PMC3218064

[B9] Diarra M. S. Silversides F. G. Diarrassouba F. Pritchard J. Masson L. Brousseau R. (2007). Impact of feed supplementation with antimicrobial agents on growth performance of broiler chickens, *Clostridium perfringens* and *Enterococcus* number, antibiotic resistant phenotype, and distribution of antimicrobial resistance determinants in *Escherichia coli*. *Appl. Environ. Microbiol.* 73 6566–6576. 10.1128/AEM.01086-07 17827305 PMC2075079

[B10] Gong J. Yu H. Liu T. Gill J. J. Chambers J. R. Wheatcroft R. (2008). Effects of zinc bacitracin, bird age and access to range on bacterial microbiota in the ileum and caeca of broiler chickens. *J. Appl. Microbiol.* 104 1372–1382. 10.1111/j.1365-2672.2007.03699.x 18201175

[B11] Hernández F. Madrid J. García V. Orengo J. Megías M. D. (2004). Influence of two plant extracts on broilers performance, digestibility, and digestive organ size. *Poult. Sci.* 83 169–174. 10.1093/ps/83.2.169 14979566

[B12] Huson D. H. Auch A. F. Qi J. Schuster S. C. (2007). MEGAN analysis of metagenomic data. *Genom. Res.* 17 377–386. 10.1101/gr.5969107 17255551 PMC1800929

[B13] Jepson R. G. Craig J. C. (2007). A systematic review of the evidence for cranberries and blueberries in UTI prevention. *Mol. Nutr. Food Res.* 51 738–745. 10.1002/mnfr.200600275 17492798

[B14] Jukes T. H. (1977). The history of the “antibiotic growth effect”. *Fed. Proc.* 36 2514–2518. 332532

[B15] Kuehn B. M. (2014). FDA moves to curb antibiotic use in livestock. *JAMA* 311 347–348. 10.1001/jama.2013.285704 24449297

[B16] Landers T. F. Cohen B. Wittum T. E. Larson E. L. (2012). A review of antibiotic use in food animals: perspective, policy, and potential. *Public Health Rep.* 127 4–22. 10.1177/003335491212700103 22298919 PMC3234384

[B17] Laube H. Friese A. von Salviati C. Guerra B. Käsbohrer A. Kreienbrock L. (2013). Longitudinal monitoring of extended-spectrum-beta-lactamase/AmpC-producing *Escherichia coli* at German broiler chicken fattening farms. *Appl. Environ. Microbiol.* 79 4815–4820. 10.1128/AEM.00856-13 23747697 PMC3754693

[B18] Levy S. (2014). Reduced antibiotic use in livestock: how Denmark tackled resistance. *Environ. Health Perspect.* 122:A160. 10.1289/ehp.122-A160 24892505 PMC4050507

[B19] Lipsitch M. Singer R. S. Levin B. R. (2002). Antibiotics in agriculture: when is it time to close the barn door? *Proc. Natl. Acad. Sci. U.S.A*. 99 5752–5754. 10.1073/pnas.092142499 11983874 PMC122845

[B20] Looft T. Johnson T. A. Allen H. K. Bayles D. O. Alt D. P. Stedtfeld R. D. (2012). In-feed antibiotic effects on the swine intestinal microbiome. *Proc. Natl. Acad. Sci. U.S.A.* 109 1691–1696. 10.1073/pnas.1120238109 22307632 PMC3277147

[B21] Mancabelli L. Ferrario C. Milani C. Mangifesta M. Turroni F. Duranti S. (2016). Insights into the biodiversity of the gut microbiota of broiler chickens. *Environ. Microbiol.* 18 4727–4738. 10.1111/1462-2920.13363 27129897

[B22] Mariat D. Firmesse O. Levenez F. Guimarăes V. Sokol H. Doré J. (2009). The Firmicutes/Bacteroidetes ratio of the human microbiota changes with age. *BMC Microbiol.* 9:123. 10.1186/1471-2180-9-123 19508720 PMC2702274

[B23] McArthur A. G. Waglechner N. Nizam F. Yan A. Azad M. A. Baylay A. J. (2013). The comprehensive antibiotic resistance database. *Antimicrob. Agents Chemother.* 57 3348–3357. 10.1128/AAC.00419-13 23650175 PMC3697360

[B24] McMurdie P. J. Holmes S. (2013). phyloseq: an R package for reproducible interactive analysis and graphics of microbiome census data. *PLOS ONE* 8:e61217. 10.1371/journal.pone.0061217 23630581 PMC3632530

[B25] Modi S. R. Lee H. H. Spina C. S. Collins J. J. (2013). Antibiotic treatment expands the resistance reservoir and ecological network of the phage metagenome. *Nature* 499 219–222. 10.1038/nature12212 23748443 PMC3710538

[B26] Oakley B. B. Kogut M. H. (2016). Spatial and temporal changes in the broiler chicken cecal and fecal microbiomes and correlations of bacterial taxa with cytokine gene expression. *Front. Vet. Sci.* 3:11. 10.3389/fvets.2016.00011 26925404 PMC4759570

[B27] Peng M. Aryal U. Cooper B. Biswas D. (2015). Metabolites produced during the growth of probiotics in cocoa supplementation and the limited role of cocoa in host-enteric bacterial pathogen interactions. *Food Control* 53 124–133. 10.1016/j.foodcont.2015.01.014

[B28] Roca I. Akova M. Baquero F. Carlet J. Cavaleri M. Coenen S. (2015). The global threat of antimicrobial resistance: science for intervention. *New Microbes New Infect.* 16 22–29. 10.1016/j.nmni.2015.02.007 26029375 PMC4446399

[B29] Saengkerdsub S. Ricke S. C. (2014). Ecology and characteristics of methanogenic archaea in animals and humans. *Crit. Rev. Microbiol.* 40 97–116. 10.3109/1040841X.2013.763220 23425063

[B30] Salaheen S. Jaiswal E. Joo J. Peng M. Ho R. OConnor D. (2016). Bioactive extracts from berry byproducts on the pathogenicity of *Salmonella* Typhimurium. *Int. Food Microbiol.* 237 128–135. 10.1016/j.ijfoodmicro.2016.08.027 27565525

[B31] Salaheen S. Nguyen C. Hewes D. Biswas D. (2014). Cheap extraction of antibacterial compounds of berry pomace and their mode of action against the pathogen *Campylobacter jejuni*. *Food Control* 46 174–181. 10.1016/j.foodcont.2014.05.026

[B32] Salaheen S. Nguyen C. Mui C. Biswas D. (2015). Bioactive berry juice byproducts as alternative and natural inhibitors for *Salmonella* Gallinarum and *Salmonella* Pullorum. *Appl. Poult. Res.* 24 186–197. 10.3382/japr/pfv021

[B33] Schmieder R. Edwards R. (2011). Fast identification and removal of sequence contamination from genomic and metagenomic datasets. *PLOS ONE* 6:e17288. 10.1371/journal.pone.0017288 21408061 PMC3052304

[B34] Simmons K. Islam M. R. Rempel H. Block G. Topp E. Diarra M. S. (2016). Antimicrobial resistance of *Escherichia fergusonii* isolated from broiler chickens. *J. Food Prot.* 79 929–938. 10.4315/0362-028X.JFP-15-575 27296596

[B35] Singh P. Karimi A. Devendra K. Waldroup P. W. Cho K. K. Kwon Y. M. (2013). Influence of penicillin on microbial diversity of the cecal microbiota in broiler chickens. *Poult. Sci.* 92 272–276. 10.3382/ps.2012-02603 23243258

[B36] Singleton V. L. Orthofer R. Lamuela-Raventós R. M. (1999). Analysis of total phenols and other oxidation substrates and antioxidants by means of folin-ciocalteu reagent. *Methods Enzymol.* 299 152–178. 10.1016/S0076-6879(99)99017-1

[B37] Thomas F. Hehemann J. H. Rebuffet E. Czjzek M. Michel G. (2011). Environmental and gut bacteroidetes: the food connection. *Front. Microbiol.* 2:93 10.3389/fmicb.2011.00093 PMC3129010 21747801

[B38] Torok V. A. Allison G. E. Percy N. J. Ophel-Keller K. Hughes R. J. (2011). Influence of antimicrobial feed additives on broiler commensal posthatch gut microbiota development and performance. *Appl. Environ. Microbiol.* 77 3380–3390. 10.1128/AEM.02300-10 21441326 PMC3126468

[B39] Treangen T. J. Koren S. Sommer D. D. Liu B. Astrovskaya I. Ondov B. (2013). MetAMOS: a modular and open source metagenomic assembly and analysis pipeline. *Genome Biol.* 14:R2. 10.1186/gb-2013-14-1-r2 23320958 PMC4053804

[B40] Turnbaugh P. J. Ley R. E. Mahowald M. A. Magrini V. Mardis E. R. Gordon J. I. (2006). An obesity-associated gut microbiome with increased capacity for energy harvest. *Nature* 444 1027–1031. 10.1038/nature05414 17183312

[B41] Tzounis X. Rodriguez-Mateos A. Vulevic J. Gibson G. R. Kwik-Uribe C. Spencer J. P. (2011). Prebiotic evaluation of cocoa-derived flavanols in healthy humans by using a randomized, controlled, double-blind, crossover intervention study. *Am. J. Clin. Nutr.* 93 62–72. 10.3945/ajcn.110.000075 21068351

[B42] Viveros A. Chamorro S. Pizarro M. Arija I. Centeno C. Brenes A. (2011). Effects of dietary polyphenol-rich grape products on intestinal microflora and gut morphology in broiler chicks. *Poult. Sci.* 90 566–578. 10.3382/ps.2010-00889 21325227

[B43] Wood D. E. Salzberg S. L. (2014). Kraken: ultrafast metagenomic sequence classification using exact alignments. *Genom. Biol.* 15:R46. 10.1186/gb-2014-15-3-r46 24580807 PMC4053813

[B44] Yang H. Hewes D. Salaheen S. Federman C. Biswas D. (2014). Effects of blackberry juice on growth inhibition of foodborne pathogens and growth promotion of *Lactobacillus*. *Food Control* 37 15–20. 10.1016/j.foodcont.2013.08.042

[B45] Zhang G. F. Yang Z. B. Wang Y. Yang W. R. Jiang S. Z. Gai G. S. (2009). Effects of ginger root (*Zingiber officinale*) processed to different particle sizes on growth performance, antioxidant status, and serum metabolites of broiler chickens. *Poult. Sci.* 88 2159–2166. 10.3382/ps.2009-00165 19762870

