# Supplementary material for: Alternative Growth Promoters Modulate Broiler Gut Microbiome and Enhance Body Weight Gain
Source: Front Microbiol. 2017 Oct 26;8:2088. doi: 10.3389/fmicb.2017.02088 (PMC5662582; doi:10.3389/fmicb.2017.02088)
Supplement: Supplementary file 1 [file Data_Sheet_1.PDF]

Supplementary Material

Alternative growth promoters modulate broiler gut microbiome and enhance body weight gain

Serajus Salaheen, Seon-Woo Kim, Bradd J. Haley, Jo Ann S. Van Kessel, Debabrata Biswas

*** Correspondence:** Debabrata Biswas: dbiswas@umd.edu

Supplementary Table 1. List of compounds in blackberry and blueberry pomace extracts from HPLC-MS analysis

Blackberry pomace extract	Blueberry pomace extract
4-Amino-2,6-dinitrotoluene	4-Amino-2,6-dinitrotoluene
bayogenin 3-O-cellobioside	3,4-Dehydrothiomorpholine-3-carboxylate
Phosphoric acid	D-Glucuronic acid
2,3-Dihydroxybenzoic acid	2,3-Dihydroxybenzoic acid
Dulcitol	Valiolone
Glucaric acid lactone	Dimethyl phosphate
ellagic acid	4-Hydroxyphenylpyruvic acid
Dimethyl phosphate	MCPA-thioethyl
2-(alpha-D-Galactosyl)-sn-glycerol 3-phosphate	Phosphoric acid
L-Galactonate	Hamamelose
1-Hydroxypentane-1,2,5-tricarboxylate	BEC
gallic acid	1,4-beta-D-Glucan
9S,10S,11R-trihydroxy-12Z-octadecenoic acid	Gallic acid
2-Dehydro-3-deoxy-D-glucarate	8-Hydroxyluteolin 7-glucoside
Rhizocticin A	Fonofos
Diethyl Oxalpropionate	alpha-D-Galactosyl-(1,1')-sn-glycerol 3-phosphate
L-Glutamate	Apigenin 7-(4''-Z-p-coumarylglucoside)
Gly-Val-OH	ent-Epifisetinidol-4beta-ol
(R)-(Homo)3-citrate	Carbon dioxide
D-Ribitol 5-phosphate	Glucaric acid lactone
Amaronol A	8-Hydroxyluteolin 7-xyloside
5-(3'-Carboxy-3'-oxopropenyl)-4,6-dihydroxypicolinate	D-Sorbitol
Glucuronic acid	Glyceraldehyde
Pyrroline hydroxycarboxylic acid	Pyrroline hydroxycarboxylic acid
6-Hydroxyluteolin 6-glucuronide	Lipoic acid, reduced
Pedunculagin	Quercetin 7-(6''-acetylglucoside)

3-Methylmuconolactone	Myricetin 3-alpha-L-arabinofuranoside
Difenoconazole	Dinoseb acetate
Glucoheptonic acid	6-Hydroxyluteolin 7-glucoside
Glyceraldehyde	2-Oxo-4-hydroxy-5-aminovalerate
1-Phosphatidyl-D-myo-inositol	L-Malic acid
Dryopteric acid	Methyl N-(a-methylbutyryl)glycine
6-Hydroxyluteolin 7-[6"-(3-hydroxy-3-methylglutaryl)glucoside]	Kaempferol 3-glucoside-7-galactoside
4,12-dihydroxy-hexadecanoic acid	Glucaric acid
Galactaric acid	Glucoheptonic acid
Methyl N-(a-methylbutyryl)glycine	Oxdemetonmethyl
Hydroquinone	p-Salicylic acid
dihydrodigoxin	6-Phosphogluconic acid
Dinor-PGD2	Syringic acid
Ribose-1-arsenate	Diethylphosphate
ent-Epifisetinidol-4beta-ol	Cinnamtannin B1
Oxdemetonmethyl	2-Dehydro-3-deoxy-D-glucarate
N-Oleoyl-L-Serine	1-Phosphatidyl-D-myo-inositol
Glu Glu Glu	5'-Butyrylphosphoinosine
Ribothymidine	Ser His Ser
2-Hydroxy-6-ketononatrienedioate	Cys Val Met
Diethylphosphate	11-Deoxytetrodotoxin
Mitoxantrone dicarboxylic acid	2-(beta-D-Glucosyl)-sn-glycerol 3-phosphate
Lipoic acid, reduced	Prostaglandin D2-biotin
3-Deoxyarabinohexonic acid	Luteolin 4'-methyl ether 7-sophoroside
Arabinonic acid	6-Methoxypulcherrimin
Phosphatidyl glycerol	1,2-beta-D-Glucuronosyl-D-glucuronate
Pretazettine	Quercetagetin 6-glucoside
1,4-beta-D-Glucan	Prephenic acid
6-Hydroxyluteolin 7-rutinoside	Dihydromyricetin
1-Deoxy-D-altro-heptulose 7-phosphate	Salicyl phenolic glucuronide
6-Phosphogluconic acid	Ellagic acid
DL-Glycerol 1-phosphate	Glu Glu Glu
Î²-Hydroxypyruvic acid	Radicicol
3-thio-Pheneacrylic Acid methyl ester	Chlordiazepoxide
RITA	2-Fluorobenzoate
32-Hydroxyrifabutin	Cellobiono-1,5-lactone
Ser Gly His	6-Hydroxyl-1,6-dihydropurine ribonucleoside
Vanilpyruvic acid	allo-Inositol
Guanosine	Allophanic acid methyl ester
Bay-K-8644	Phloionolic acid
Isoscutellarein	Î²-Hydroxypyruvic acid

Orbencarb	Chlorogenic Acid
L-beta-aspartyl-L-phenylalanine	furazolidone
Tautomycin	Biflorin
Furosemide glucuronide	Glutathione, oxidized
UDP-D-galacturonate	Kaempferol 3-[2''',3''',4'''-triacetyl-alpha-L-arabinopyranosyl-(1->6)-glucoside]
N-Acetyldemethylphosphinothricin	Apionic acid
Ser Asn Asn	12-Octadecenoic acid, 9,10,18-trihydroxy-; 9,10,18-Trihydroxyoctadec-12-enoic acid
Xylitol	Kaempferol 3-(2'',6''-di-(E)-p-coumarylglucoside)
Hygromycin A	Inucrithmin
Harrisonin	Galactosylglycerol
Sulprofos	b-D-Glucopyranuronic acid, 1-(6-methoxy-2-naphthaleneacetate)
L-Malic acid	3-Amino-2-oxopropyl phosphate
Glyceryl phosphate	Pyraclofos
Glutathione, oxidized	Arabino-galactose
Pinocembrin	Gln Glu Glu
Met Asp Met	Kaempferol 3-[2''',3''',5'''-triacetyl-alpha-L-arabinofuranosyl-(1->6)-glucoside
GDP-4-Dehydro-6-deoxy-D-mannose	DL-Benzylsuccinic acid
Ser His Ser	Xylitol
Alnusiin	Isobrucein A
Hydroxymethylphosphonate	2,4-Dichloro-3-oxoadipate
3,5,7,2',5'-Pentahydroxyflavone	Chlorate
4-Naphthalimidobutyric Acid	DL-Glycerol 1-phosphate
Duartin, Dimethyl Ether	Uridine monophosphate (UMP)
CDP-N-methylethanolamine	Gulonolactone
Isorhapontin	Deoxyribonolactone
2-Hydroxy-3-carboxybenzalpyruvate	Plantamajoside
Uridine	6,7-dihydroxy-4-oxo-2-heptenoic acid
Furocaespitane	3,3-Dimethylglutaric acid
Amaronol B	7-(Acetyloxy)-3-(3-pyridinyl)-2H-1-benzopyran-2-one
Protionamide sulfate	8,11-octadecadienoic acid
DL-Î±-Lipoic Acid	N-Acetylglucosamine 6-phosphate
D-4-Hydroxy-2-oxoglutarate	Diglycolic acid
Bongkrekic acid	12-hydroxy-10-octadecynoic acid
Pseudouridine 5'-phosphate	D-threonic acid
2-Hydroxy-6-oxo-6-(2-carboxyphenyl)-hexa-2,4-dienoate	Piperonyl butoxide
Asulam	Cinnamtannin D1
Mesaconic acid	2,6-Dihydroxy-4-Methoxytoluene

PR-toxin	Aminopterin
Hydroxyphthioceranic acid (C36)	Pyrophosphate
albendazole (II)	1-Caffeoyl-4-deoxyquinic acid
PE(12:0/0:0)	4-phenyl-5-methyl-1,2,3-Thiadiazole
Pandaroside B	Theogallin
9,10-dioxo-octadecanoic acid	Scopolin
luteolin 7-(2''-p-coumaroylglucoside)	2H-1-Benzopyran-6-acetic acid, 7-hydroxy-8-methoxy-2-oxo-
7-alpha-D-Ribosyladenine 5'-phosphate	Mitoxantrone dicarboxylic acid
Asp-His-OH	3-Hydroxy-4-methoxyphenylacetic acid
6-Hydroxyl-1,6-dihydropurine ribonucleoside	Vidarabine
L-Ascorbic acid-2-glucoside	Triacetate
3-Amino-3-(4-hydroxyphenyl)propanoate	12,13S-epoxy-9Z,11-octadecadienoic acid
Leptodactylone	2-Aminoethylphosphonic acid
Undecanedioic acid	Ethyl glucuronide
2'-Hydroxy-3',4',6',3,4-pentamethoxychalcone	Myricetin 3-(4''-malonylrhamnoside)
Estradiol-17-phenylpropionate	Dinitramine
7-(Acetyloxy)-3-(3-pyridinyl)-2H-1-benzopyran-2-one	Aurasperone D
Zearalenone	Benfotiamine
Soyasaponin III	Glyceryl phosphate
N-Acetylmuramoyl-Ala	Acetaminophen-cysteine
2',3'-Cyclic GMP	Dryopteric acid
3-(2,3-Dihydroxyphenyl)propanoate	(E)-C-HDMAPP
Dihydroclavaminic acid	Hydroxymethylphosphonate
Isowighteone	9E,12Z,15Z-octadecatrienoic acid
Nap-Ser-OH	Proanthocyanidin A2
Gibberellin A15	Sinapoyltartronate
Glu Glu	Tetroquinone
Robinetin	Khellol glucoside
10-Deacetylbaccatin III	CBS 113A
Geraniin	Quercetin 7-xyloside
Stipitatate	Monoethyl phthalate
Gln Thr Asp	5-Dehydroshikimate
6-Lactoyltetrahydropterin	2,5-Furandicarboxylic acid
Sapropterin	3,4-Dihydroxyphthalate
4-Hydroxybenzaldehyde	Pyrifenox
Glutaconic acid	methyl (+)-7-isojasmonate
Carboxyltolmetin Diglucuronide	Echioidinin 2'-(6''-acetylglucoside)
Floxacillin	L-Ascorbic acid-2-glucoside
Mallotinic acid	Asp-Trp-OH
7-hydroxy-5-heptynoic acid	Eupachlorin

Limonoate	Apigenin 7-(2",3"-diacetylglucoside)
Tellimagrandin I	Mascaroside
Anhydrobrazilic Acid	Busulfan
butabarbital	3-Amino-3-(4-hydroxyphenyl)propanoate
Phellodensin D	Strictosamide
chlorogenic Acid	Myricetin 3,4'-dimethyl ether 3'-xyloside
(S)-3-Sulfolactate	2,6,3',4'-Tetrahydroxy-2-benzylcoumaranone
10-hydroxy-16-oxo-hexadecanoic acid	meso-Tartaric acid
Apigeniflavan 5-O-xyloside	Irigenol
Thiophanate	Nedocromil
Val Asn Lys	PG(12:0/0:0)
Triacetate	1,4-Bis(chloromethoxymethyl)benzene
gamma-Pentachlorocyclohexene	Stipitatate
12-Octadecenoic acid, 9,10,18-trihydroxy-; 9,10,18-Trihydroxyoctadec-12-enoic acid	Elesclomol
Dehydroascorbic acid	Miglitol
cholesterol sulfate	9S,10-epoxy-10,12Z-octadecadienoic acid
Chloromethiuron	4-Carboxy-2-hydroxy-6-methoxy-6-oxohexa-2,4-dienoate
2,3,4-Trihydroxybenzylhydrazide	EA4
Y 23684	Cys Asn
Haematommic Acid	EUK 118
Fucofuroeckol B	Quercetin 3-vicianoside
Formylpyruvate	3,4,2',3',4',6',alpha-Heptahydroxychalcone 2'-glucoside
4-Methoxyglucobrassicin	4-Deoxytetronic acid
12,13S-epoxy-9Z,11-octadecadienoic acid	(S)-3-Sulfolactate
Levoamine (Chloramphenicol D base)	Orbencarb
Meptazinol glucuronide	2-Hydroxy-6-oxonona-2,4-diene-1,9-dioate
9-hydroperoxy-12,13-epoxy-10-octadecenoic acid	(S)-4-Hydroxymandelate
Benfotiamine	2-Deoxy-L-arabinose
Î”17-6-keto PGF1Î±	D-Galactose 6-sulfate
Metanephrine	Kaempferol 3-(2"-feruloylglucosyl)-(1->2)-(6"-malonylglucoside)
Northienamycin	Diadenosine diphosphate
Fluroxypyr	methyl 11-(3,5-epidioxy-2-ethyl-cyclopentyl)-9-hydroperoxy-10-undecenoate
epi-4'-hydroxyjasmonic acid	Pseudopurpurin
3-Ketosucrose	Dumosol
Acacetin 7-(2G-rhamnosyl)-rutinoside	bayogenin 3-O-cellobioside
Gambiriin B1	Hygromycin A
meglumine	(R)-(Homo)2-citrate
Ticarcillin	Epicatechin Monogallate

3-Indolyllactic acid	Deoxyribose 5-phosphate
Asiatic acid	Phenylephrine 3-O-sulfate
3-Hydroxy-2-methylpyridine-4,5-dicarboxylate	meglumine
3-Vinylbacteriochlorophyllide d	4-p-Coumaroylquinic acid
trans-O-Hydroxybenzylidenepyruvate	L-Glutamate
Hydroxypentobarbital	gamma-Pentachlorocyclohexene
3-Methyl-2-butenoic acid	D-Erythritol 4-phosphate
Elesclomol	dTMP
2-Hydroxy-4,5,6-trimethoxydihydrochalcone	L-beta-aspartyl-L-phenylalanine
Allicin	9R,10S,18-trihydroxy-stearic acid
Epigallocatchin 3,4',-di-O-gallate	5,7,2',5'-Tetrahydroxy-6-methoxyflavanone
5,7-Dihydroxychromone	Sulprofos
Pro Pro Phe	Remifentanil
Cefacetrile	Spicatin
Î²-lactic acid	Myoinositol 1-phosphate
D-threonic acid	4,14-dihydroxy-octadecanoic acid
DL-3-Phenyllactic acid	Tricrozarin A
Chamissonin diacetate	Imidacloprid
Spinochalcone C	8-Oxodeoxycoformycin
7-[2-Trifluoromethyl-4-(2-hydroxyphenyl)-1,3-dioxan-cis-5-yl]-hept-5z-enoic Acid	3-Deoxy-D-manno-octulosonate
Met His Phe	IACI
Acephate	5-Dehydro-D-fructose
Hallactone B	Î²-lactic acid
(S)-2-O-Sulfolactate	(S)-ACPA
2-Furoic acid	2-(Î±-D-Mannosyl)-3-phosphoglycerate
Oxoproflaxacin	Trisphaeridine
Nigerose (Sakebiose)	1-Hydroxypentane-1,2,5-tricarboxylate
Chicoric acid	9-Nitroanthracene
Estriol-17-glucuronide	Asp Val Asn
Brusatol	Ethalfluralin
12-hydroxy-10-octadecynoic acid	Carthamin
D-Serine	2,4,6-trimethyl-3,5-dinitrobenzonitrile
Tyr Pro Pro	Formylpyruvate
Propanoyl phosphate	PA(13:0/0:0)
17alpha-estradiol 3-glucosiduronic acid	Cyazofamid
3'-Ketolactose	Asp Thr Asp
Prenyl-L-cysteine	2,3-DCPE
Glucoiberverin	methyl 13,15-epidioxy-12-hydroperoxy-9Z,16E-octadecadienoate
SAICAR	Erythrose
Purine	Methanesulfonic acid

1-Phenyl-5-mercaptotetrazole	9,10-dihydroxy-hexadecanoic acid
9S-hydroxy-12R,13S-epoxy-10E,15Z-octadecadienoic acid	5-Amino-6-(5'-phosphoribitylamino)uracil
3-Methyluridine	5-O-Feruloylquinic acid
4-Nitro-3-(trifluoromethyl)aniline	3-(2-Carboxyethenyl)-cis,cis-muconate
Chlorate	Glu Trp His
9S,10-epoxy-10,12Z-octadecadienoic acid	PG(14:0/13:0)
4-Bromocatchol	(1R,6R)-6-Hydroxy-2-succinylcyclohexa-2,4-diene-1-carboxylate
1,2,3,6-Tetrakis-O-galloyl-beta-D-glucose	butabarbital
Gly Thr Cys	4-Heptyloxyphenol
PG(12:0/0:0)	CDP-N-methylethanolamine
o-Benzosemiquinone	Quercetagetin 7-(6''-(E)-caffeoylglucoside)
abrusoside A	Diflunisal
Gambiriin B2	Bumetanide
Dacthal	5,7,2'-Trihydroxy-3,6,4',5'-tetramethoxyflavone
(S)-3-(Imidazol-5-yl)lactate	Phosphonoacetate
5'-Butyrylphosphoinosine	Epigallocatechin-(4beta->8)-epicatechin-3-O-gallate ester
Val Trp Glu	Glu Asn Glu
Avermectin A2a aglycone	Haplogenin
Niveusin C	Xylocarpus A
Nitrotyrosine	2'-Hydroxy-3',4',6',3,4-pentamethoxychalcone
Muramic acid	Levoamine (Chloramphenicol D base)
2-Chloro-1,1,2-trifluoroethyl ethyl ether	3-O-a-L-Fucopyranosyl-D-glucose
2-(4'-Methylthio)butylmalic acid	Wharangin
Piscidic Acid	CGP 28-392
Glyoxylic acid	Cinchonain Id
D-Prephenyllactate	3-Methyl-2-butenoic acid
MID42395:26,27-diethyl-1Î±,25-dihydroxy-22-thiavitamin D3 / 26,27-diethyl-1Î±,25-dihydroxy-22-thiachol	Bis-D-fructose 2',1:2,1'-dianhydride
2-Deoxy-L-arabinose	7-alpha-D-Ribosyladenine 5'-phosphate
10-hydroperoxy-8E,12Z-octadecadienoic acid	1-Aminomethylphosphonic acid
Citraconic acid dimethyl ester	Corrinoid
Dechloroethylcyclophosphamide	2-Hydroxybutane-1,2,3-tricarboxylate
Magnesium protoporphyrin	1-(Indol-3-yl)propanol 3-phosphate
beta-D-4-Deoxy-delta4-GlcA-(1->4)-beta-D-Glc-(1->4)-alpha-L-Rha-(1->3)-beta-D-Glc	D-Serine
cis-2,3-Dihydro-2,3-dihydroxy-4'-chlorobiphenyl	Actinonin
7,4'-Dihydroxyflavan	Leiocalycin
3',5'-Cyclic AMP	Î±-D-Glutamyl phosphate
Adenosine 5'-phosphoramidate	Purine

Asn Tyr Gly	Fusarenone X
Pro His His	Penicillenic acid
1-Aminomethylphosphonic acid	LY255283
Hydroxyflutamide	N-Methylethanolamine phosphate
Paratocarpin B	Gossypetin 3-methyl ether
Digoxigenin bisdigitoxoside	Quercetagetin 3'-methyl ether
Gibberellin A7	Formononetin 7-O-glucoside-6''-O-malonate
4-Heptyloxyphenol	Baileyin
Propane-1,2,3-tricarboxylate	(R)-(Homo)3-citrate
L-Phenylalanine	Okanin 4'-(6''-p-coumarylglucoside)
Vicianose	Metamitron
epi-Tulipinolide diepoxide	1-Methylhypoxanthine
3'-Methyl-2',4',6'-trihydroxydihydrochalcone	4-Hydroxy-6-methylpyran-2-one
2-Acetolactic acid	Fluorescein monoglucuronide
Petasitenine	5,7,3',4',5'-Pentahydroxyflavanone
Cephalotaxine	2-Furoic acid
L-Glyceric acid	2-Butanone, 4-[6-(sulfooxy)-2-naphthalenyl]-
Epiafzelechin 3-O-gallate	Fastigilin B
D-Glycerate 2-phosphate	Caffeic acid 3-glucoside
Cys Asp His	Norepinephrine sulfate
Adenosine 3',5'-bisphosphate (PAP)	Urate D-ribonucleotide
1-(beta-D-Ribofuranosyl)-1,4-dihydronicotinamide	5-amino-1-(5-phospho-D-ribosyl)imidazole-4-carboxylate
4-Oxoglutaramate	Albendazole-beta-hydroxysulphone
4-[2-(5-Carboxy-2-hydroxy-3-methoxyphenyl)-2-oxoethylidene]-2-hydroxy-2-pentenedioate	Guanosine
6-Cyano-7-nitroquinoxaline-2,3-dione	9S-hydroxy-12R,13S-epoxy-10E,15Z-octadecadienoic acid
6-(Ethylthio)purine	Diphenylcarbazide
14-Hydroxypergolide glucuronide	Flakinin A
5-Hydroxymethylfloxacillin	2-hydroxy-10-undecenoic acid
Fastigilin B	Pyrocatechol glucuronide
meso-Tartaric acid	Adenosine 5'-phosphoramidate
PE(P-20:0/21:0)	2-Dehydro-D-xylonate
Ser-His-OH	Nap-Asp-OH
Nicorandil	Vitamin B6
Senecionine N-oxide	4-(3,5-Diphenylcyclohexyl)phenol
PI(18:0/20:2(11Z,14Z))	Gossypetin 3,3'-dimethyl ether
Dihydromilletenone methyl ether	Methylmalonic acid
Brompheniramine (monodemethylated)	3-Deoxy-D-manno-octulosonate 8-phosphate
2,4-Dihydroxytacrine	D-4-Hydroxy-2-oxoglutarate
Filiforminol	Dantrolene

Ipolamiide	Mesquitol-4beta-ol 3,8-dimethyl ether
5'-Oxoinosine	Nitrotyrosine
Eucalyptin	Futalosine
5,7,2',5'-Tetrahydroxy-6-methoxyflavanone	Fumarprotocetraric Acid
Licodione	PR-toxin
PQQ	Tyr Phe Arg
3-butyrl propionic acid	Isoscutellarein 4'-methyl ether 8-glucoside
Temurin	Ikarisoside E
124-1	5,7,4',5'-Tetrahydroxy-3,6,8,2'-tetramethoxyflavone
Eudesmic Acid	100-2
Sphagnum acid	ensulizole
Uric acid	3',4',5'-Trimethoxyflavone
5-Azacytidine 5'-monophosphate	2',3'-Cyclic adenosine monophosphate
Bis(glycerophospho)-glycerol	D(-)-Î²-hydroxy butyric acid
Soyasapogenol B 3-O-D-glucuronide	Nigerose (Sakebiose)
3-Dehydroquinic acid	Fumaric acid
10-hydroxy-hexadecan-1,16-dioic acid	3'-Sialyllactosamine
Pubescenol	UDP-2,4-bis(acetamido)-2,4,6-trideoxy-beta-L-altropyranose
Deoxythymidine 5'-diphosphate (dTDP)	3-hydroxy-suberic acid
Anthothecol	5-O-Methylembelin
Methyl N-butyrylglycine	Apigenin 7-glucuronide-4'-rhamnoside
Phosphonoacetate	AG-82
Difluprednate	Digitalose
Casuarictin	Scoparin 2''-O-xyloside
L-Tyrosine methyl ester 4-sulfate	3-Indolyllactic acid
2-Butanone, 4-[6-(sulfooxy)-2-naphthalenyl]-	TyrMe-TyrMe-OH
1-(5'-Phosphoribosyl)-5-formamido-4-imidazolecarboxamide	Propanoyl phosphate
Gln Gln Ser	Orientin 7-O-caffeate
Actinonin	9,10-dioxo-octadecanoic acid
L-Glutamyl 5-phosphate	Robinetin
Kaempferol 3-sulfate-7-alpha-arabinopyranoside	Thr-Ser-OH
Pectolinarigenin 7-glucuronide	2-Hydroxyhepta-2,4-dienedioate
Isobrucein A	Ciprofibrate
Asn-Asn-OH	8-oxo-nonanoic acid
tannin	2-Carboxy-D-arabinitol 1-phosphate
6-Acetyl-D-glucose	Sulfaphenazole
Kanokoside A	Neoglucobrassicin
2,3-dinor-11b-PGF2Î±	Nitromide
1-Amino-2-methylanthraquinone	Iriskumaonin

Papyramine	Dihydrorobinetin
1-O,2-O,6-O-Trigalloyl-beta-D-glucose	Vanillin
Rhamnellaflavoside C	2-Formaminobenzoylacetate
Tetracenomycin D3	D-Î±-Hydroxyglutaric acid
Leu Cys Met	5-O-Caffeoylshikimic acid
D-Î±-Hydroxyglutaric acid	2-Acetolactic acid
Wanepimedoside A	Ketoconazole Metabolite (1-Piperazinecarboxaldehyde, 4-[4-[[2-(2,4-dichlorophenyl)-2-(1H-imidazol-1-
5-Hydroxy-2-oxo-4-ureido-2,5-dihydro-1H-imidazole-5-carboxylate	Methylorsellinic Acid, Ethyl Ester
11,13-dimethoxy-12-hydroxy-9-octadecenoic acid	2-Hydroxypropylphosphonate
Cefazolin	Hydroquinone
5R-hydroxy-hexanoic acid	2-Protocatechoylphloroglucinolcarboxylate
Deoxymiroestrol	Purpurin
5-Dehydro-D-fructose	Geniposidic acid
3-Deoxy-D-manno-octulosonate	Iminoaspartic acid
2-(Hydroxymethyl)-3-(acetamidomethylene)succinate	Glu Thr Leu
Trp Glu Glu	Quercetin 3-(6"-ferulylglucoside)
Chrysoeriol 7,4'-diglucuronide	Val Trp Glu
Eicosanoyl-EA	Methylthiobenzoic acid
Fraxetin	Tebupirimfos
Trolox	Inabenfide
Irigenol	Oxaloglutarate
Phloionolic acid	Propane-1,2,3-tricarboxylate
Nigrescin	L-Arabinono-1,4-lactone
Gossypetin 8-glucoside-3-sulfate	Croconazole
Phenethylamine glucuronide	Hydroxyitraconazole
Purine mononucleotide	Tephrodin
3-Indolebutyric acid	Fentin hydroxide
Gualenate	Phenylmethanesulfonyl fluoride
Cucurbitacin H	His Cys Asp
Spenolimycin	Eudesmic Acid
AX 048	2,6-Dinitrotoluene
1-Caffeoyl-beta-D-glucose	2-chlorohexadecanol
apigenin 7-(4"-Z-p-coumarylglucoside)	MID73253:alpha-(1,2-Dihydroxyethyl)-1,2,3,4-tetrahydro-7-hydroxy-9-methoxy-3,4-dioxocyclopenta[c][1]
Tefluthrin	2-Hydroxy-6-ketononatrienedioate
Sphenostylin D	cis,cis-Muconic acid
Guanosine 3'-phosphate	Hydroxyflutamide

Porphobilinogen	EUK 134
Dihydromyricetin	Phellatin
Imipenem	Thiabendazole
Eriodictyol 7-O-glucoside	Ourateacatechin
N-Desethylquinagolide	gibberellin A28
4-Carboxy-2-hydroxy-6-methoxy-6-oxohexa-2,4-dienoate	4-oxo-nonenal
Neoglucobrassicin	Nap-Ser-OH
Sufentanil	Hydroxypentobarbital
4'-O-beta-D-Glucosyl-cis-p-coumarate	Dibutyl phthalate
phenol	Patuletin 7-galactoside
7H-Furo[3,2-g][1]benzopyran-7-one, 2,3-dihydro-2-hydroxy-9-methoxy-	Galactose-beta-1,4-xylose
Nalbuphine-6-sulfate	Methyl-2-alpha-L-fucopyranosyl-beta-D-galactoside
Cellobiono-1,5-lactone	a-Methyl-3,4-dihydroxyphenylpropionic acid
Machaerol C	Isoorientin 2''-O-(E)-caffeate
ethotoin	Flupoxam
Methanesulfonic acid	4,4'-Biphenyldithiol
Tetroquinone	Dolichyl phosphate D-mannose
5-Aminoimidazole-4-carboxamide-1-Î²-D-ribofuranosyl 5'-monophosphate	Xanthine
Albendazole-beta-hydroxysulphone	Glyceric acid
5-Hydroxy-6-methoxyindole glucuronide	MDL 73492 sulfate
PtdIns-(3,4,5)-P3 (1,2-dihexanoyl)	Pantothenic Acid
O-Phospho-4-hydroxy-L-threonine	Vat Yellow 4
Gliclazide	TyrMe-Asp-OH
Scutellarein	1-(beta-D-Ribofuranosyl)-1,4-dihydronicotinamide
Inosine 5'-monophosphate (IMP)	4-(Î²-D-Glucosyloxy)benzoate
Cys His Ser	Styrene cis-glycol
3,4-Dihydroxyphenylglycol O-sulfate	D-myo-Inositol-1,5-diphosphate
Haemocorin	Myricetin 3-galactoside-3'-rhamnoside
Citrinin	Guanosine 3'-phosphate
11-O-Demethylpradinone I	3'-Sialyllactose
9,11alpha-epoxy-6alpha-acetoxy-cholest-7-en-3beta,5alpha,19-triol	CAY10608
(Â±)-Mevalonolactone	3-Ethylmalate
Sucrose-6-phosphate	6-hydroxy-heptanoic acid
6-Deoxy-5-ketofructose 1-phosphate	9-Amino-1,2,3,4-tetrahydroacridine
(9R,13R)-1a,1b-dinor-10,11-dihydro-12-oxo-15-phytoenoic acid	Ser-Thr-OH
Carfentrazone-ethyl	3-Methyl-2-thiohydantoin

13(1)-Hydroxy-magnesium-protoporphyrin IX 13-monomethyl ester	TyrMe-Phe4Cl-OH
Phenylephrine 3-O-sulfate	Formoterol
Suberic acid	(2R,3S)-2,3-Dimethylmalate
2,3-Dihydroxy-3-methylvaleric acid	1-O-Galloyl-beta-D-glucose
Sinapoyl malate	PtdIns-(3,5)-P2 (1,2-dioctanoyl)
9,10-Dihydro-10-(4-hydroxyphenyl)-pyrano[2,3-h]epicatchin-8-one	Sepiol
Dihydroartemisinin	Asn Cys Asn
PG(14:0/13:0)	Tyr Trp Tyr
2-oxo-undecanoic acid	8S-hydroxy-9Z,12Z-octadecadienoic acid
Ser-Thr-OH	6-Hydroxy-5-methyl-3',4',5'-trimethoxyaurone-4-O-alpha-L-rhamnopyranoside
2-Thiopheneacetic acid, 5-(hydroxyphenylmethyl)-Î±-methyl-	Glabrol
Eupacunin	4-Amino-7-chloroquinoline
Iminoaspartic acid	2-(o-Carboxybenzamido)glutaramic acid
N6-Methyl-2'-deoxyadenosine	PA(14:1(9Z)/0:0)
Artesunate	Amaronol B
2-Oxo-4-methylthiobutanoic acid	4-Hydroxy-2-butynal
n-valeryl acetic acid	Furfural diethyl acetal
apigenin 7-(3''-p-coumaroylglucoside)	Diethyl phenyl phosphate
Swietenolide-3-Acetate	Erythrono-1,4-lactone
Phenyl glucuronide	11,13-dimethoxy-12-hydroxy-9-octadecenoic acid
2',3'-Cyclic UMP	Hypericin
3-Hydroxy-4-methoxyphenylacetic acid	D-Glycerate 3-phosphate
2,3-dinor Thromboxane B1	MS-275
Prontosil	MID68716:N-(4-Chloro-3-methyl-5-isothiazolyl)-N-methyl-2-[p-[(alpha,alpha,alpha-trifluoro-p-tolyl)ox
5,6,7,3',4'-Pentahydroxy-8-methoxyflavone 7-apioside	O-Phospho-L-threonine
Senkirkine	Cinchonain Ib
N-(6-Oxo-6H-dibenzo[b,d]pyran-3-yl)maleamic acid	Inosinic acid
2-(Î±-D-Mannosyl)-3-phosphoglycerate	Kaempferol 3-(2''-p-coumarylglucoside)
Uridine 5'-diphosphoglucuronic acid	L-Arginine
N4-Acetylsulfadimidine	Val Met Lys
Remifentanil	3Î±-Hydroxydeoxodihydrogedunin
Neoplathymenin	3,5-Pyridinedicarboxylic acid, 2-(hydroxymethyl)-6-methyl-4-(2-nitrophenyl)-, 5-methyl ester
Kaempferol 3-glucoside-7-galactoside	Aminofurantoin

dihydrophaseic acid 4-O-beta-D-glucoside	Acacetin 7-glucuronide
Protofarrerol	Xanthene-9-carboxylic acid
Met Ser Met	S-Adenosylhomocysteine
Indolylmethylthiohydroximate	Pseudouridine 5'-phosphate
D-(+)-Xylose	4R-hydroxy-octanoic acid
Vidarabine	Sorbose 1-phosphate
Dibutyl phthalate	2-Hydroxyethanesulfonate
Balofloxacin	11-O-Demethylpradinone II
Adenosine-3'-monophosphate	Quercetagetin hexamethyl ether
Lys-Tyr-OH	Glibornuride M2 (p-carboxyglibornuride)
Lappaol C	4-Nitrophenol
8-Hydroxyquercetagetin	Allicin
2-Formaminobenzoylacetate	Isoxicam
Tagetiin	Quinic acid
Arg Phe Arg	6-Deoxy-5-ketofructose 1-phosphate
2,5-Furandicarboxylic acid	Glutinosone
Xylocarpus A	Glu Asp
24-Nor-5Î²-cholane-3Î±,7Î²,22,23-tetrol	6-C-Galactosylisoscutellarein
2-Amino-5-formylamino-6-(5-phospho-D-ribosylamino)pyrimidin-4(3H)-one	Nodifloretin
Met Phe Ser	Kaempferol 3,4'-dixyloside
Perindoprilat	dIMP
Furfural diethyl acetal	Leinamycin
3,4,5,2',4',6'-Hexahydroxychalcone 2'-glucoside	3-Methylmuconolactone
7-Hydroxy-6-methyl-8-ribityl lumazine	Chamissonin diacetate
Met Phe Glu	Piroxicam
Hexythiazox	Dechloroethylcyclophosphamide
(2R,3S)-2,3-Dimethylmalate	20,21,21-Trifluoro-3-methoxy-19-nor-17alpha-pregna-1,3,5(10),20-tetraen-17-ol
3-Isochromanone	N-Acetyl-5-hydroxysulfapyridine
L-Arginine	9-hydroperoxy-12,13-epoxy-10-octadecenoic acid
Prostaglandin D2-biotin	Nizatidine
2-Furylmercury chloride	CBHA
MC-3761	4'-Chloroaurone
xanthine	Picein
5-Acetoxypalisadin B	p-Chlorophenylalanine
2,3-Dinor-6-keto-PGF1a	SAICAR
2-Formylglutarate	AS-605240
6-Demethoxytangeritin	CP 80633
Asn Ser Asp	Amaronol A
4-Deoxytetronic acid	N-Monomethyl-2-aminoethylphosphonate
Zonisamide	Genipin

Plaunol D	Rosavin
EUK 134	Oxaburimamide
1,4-Bis(chloromethoxymethyl)benzene	Hydantoin-5-propionic acid
Erythrono-1,4-lactone	Eupalitin 3-galactoside
8-Hydroxy-7-methylguanine	WIN54954
cis-3-(6-Hydroxy-7-methoxy-5-benzofuranyl)acrylic acid glucuronide	4,4'-Thiodianiline
methyl (+)-7-isojasmonate	6-Hydroxyluteolin 7-[6''-(3-hydroxy-3-methylglutaryl)glucoside]
4-Hydroxylamino-2,6-dinitrotoluene	2-Methylpropanoyl phosphate
Gulonolactone	5-(4-Acetoxybut-1-ynyl)-2,2'-bithiophene
4-trans-Hydroxyglipizide	Isorhamnetin 3-(6''-(E)-sinapoylsophoroside)
Methyl caffeate	Cyflufenamid
quinoline-3-carboxamides	2-Keto-glutaramic acid
N-Dealkylzuclopenthixol sulfoxide	L-Phenylalanine
Primin	Thr Ser Cys
Quinagolide	4-Hydroxy-desmethylclobazam
His Cys Asp	Quebrachitol
Probenazole	Thionazin
Embelin	11-oxo-octadecanoic acid
(+)-Iridodial	(Z)-But-1-ene-1,2,4-tricarboxylate
Phenacemide	Carboxyltolmetin Diglucuronide
Pilosanol A	5-Hydroxy-3,7,2',4',5'-pentamethoxyflavone
Aloesin	6-Acetyl-D-glucose
Bruceine D	Embelin
2-(5'-Methylthio)pentylmalic acid	Sulfameter
3-Methyl-2-thiohydantoin	4-Hydroxybenzaldehyde
Cyflufenamid	Quercimeritrin
Thr-Ser-OH	Homoisocitrate
(+)-Syringaresinol O-beta-D-glucoside	Bitalosidin
(Z)-But-1-ene-1,2,4-tricarboxylate	tetranor-PGEM
b-D-Glucopyranosiduronic acid, 3-(6-hydroxy-2-naphthalenyl)-1-methylpropyl	Metanephrine
2-hydroxy-decanedioic acid	N-Benzylacetamidine
aluminum acetate	His Thr His
Icariside II	Cys Ser
Asp-Ser-OH	Crufomate
1-Hydroxytacrine glucuronide	6-Methoxyluteolin 7-glucuronide
CDP-3,6-dideoxy-D-mannose	Fentin acetate
(S)-4-Hydroxymandelate	4-Hydroxyphenylglyoxylate
Tephcalostan C	5-Hydroxy-2-oxo-4-ureido-2,5-dihydro-1H-imidazole-5-carboxylate
Cucurbitacin L	1-Naphthoic acid glucuronide

Pseudouridine	7-Hydroxy-6-methyl-8-ribityl lumazine
CAY10608	LysoPE(0:0/18:2(9Z,12Z))
3D-(3,5/4)-Trihydroxycyclohexane-1,2-dione	Sulfolane
2,6-Diisopropylhydroquinone	27-nor-24S-methylcholestan-3beta,4beta,5alpha,6alpha,7beta,8beta,14alpha,15alpha,24-nonol
Debromohymenialdisine	Methaphenilene
Rosavin	Hydroxyphthioceranic acid (C36)
OA-6129 D	N'-Nitrosoanatabine
5-(4-Acetoxybut-1-ynyl)-2,2'-bithiophene	PD 98059
Fluoroacetamide	Kinamycin D
Cimifugin	4-Hydroxycinnamyl alcohol 4-D-glucoside
1-Propene, 1,3,3,3-tetrafluoro-2-(fluoromethoxy)-1-methoxy-, (Z)- (Compound C)	Nitrogen mustard N-oxide
3-hydroxy-cis,cis-muconic acid	Quercetin 3-galactoside-7-xyloside
Vernomenin	2,2-Dimethylglutaric acid
Inosine	Panfuran S
1,2-Dihydroxytacrine	Sulindac glucuronide
LY255283	Asp Asp Ser
Sulfometuron	N-Desmethyldiazepam (Nordazepam)
N-Acetyl-9-O-lactoylneuraminic acid	Porphobilinogen
3beta-(Acetyloxy)-5beta-methyl-6beta-chloroestr-9-en-17-one	Sciadopitysin
luteolin 7-galacturonide-4'-glucoside	Blue pigment
Glu Ala Glu	Ile-Phe4Cl-OH
Plocamene D	7-Deshydroxypyrogallin-4-Carboxylic Acid
3,4-dihydroxymandelate	Succinoadenosine
FucÎ±1-2GalÎ²1-4GlcNAcÎ²-Sp	2(Î±-D-Mannosyl)-D-glycerate
3-Ethylmalate	3,4,2',4',alpha-Pentahydroxychalcone
Ketoconazole	Cys Tyr Asn
Inucrithmin	Anastatin A
Phe Tyr Cys	PE(O-20:0/22:0)
2,4-Dichlorophenoxyacetic Acid, Methyl Ester	(-)-Columbianetin
Lappaol A	Acyclovir (8-hydroxy-9-(2-hydroxythoxymethyl)guanine
4,3'-Hydroxy-2',4',5',6'-methoxychalcone	9-oxo capric acid
5,7,3',4'-Tetrahydroxy-3,6,8,5'-tetramethoxyflavone	9S,10S,11R-trihydroxy-12Z-octadecenoic acid
1-O-Sinapoyl-Î²-D-glucose	Ile Arg Ala
5,6,3'-Trimethoxyflavone	3-(5'-Methylthio)pentylmalic acid
1-Hydroxyhexane-1,2,6-tricarboxylate	2,3-Dihydroxy-3-methylvaleric acid
Parthenin	3-propylmalic acid
epi-dihydrophaseic acid	Maesopsin

Depdecin	Tyr Cys
Genipin	trans,trans-hepta-2,4,6-trienoic acid
Dipyrocetyl	Met-Phe-OH
2-Carboxy-2-hydroxy-8-carboxychromene	Bis(glycerophospho)-glycerol
3,6-Dideoxy-3-oxo-dTDP-D-glucose	Stachyose
Flubenzimine	Gardoside
Dibutyl succinate	Guibourtinidol-4alpha-ol
Galactosylglycerol	7,8-Dihydroxycoumarin
P1,P4-Bis(5'-adenosyl) tetraphosphate (ApppppA)	Flutolanil
Pifithrin-Î±	(+)-Iridodial
2-Hydroxyethanesulfonate	Etidronic acid
5,5'-Dehydrodivanillate	Diisopropyl sulfate
Pirenzepine	Butamifos
1-O,6-O-Digalloyl-beta-D-glucose	Flumetsulam
Compactin diol lactone	Met Thr Trp
Prazosin	Chlorpromazine sulfone
Myricetin 3-galactoside-3'-rhamnoside	L-Tyrosine methyl ester 4-sulfate
Glu Asn Leu	Hypotaurocyamine
Phaseolic acid	2,3-Dinor-TXB2
Trans-2, 3, 4-Trimethoxycinnamate	Isorhamnetin 4'-glucoside
Sporidesmin	GW 409544
beta-Alaninebetaine	Madecassic Acid
8,11-octadecadienoic acid	Inositol cyclic phosphate
Glucocheirolin	SC-58125
4-Hydroxycinnamic acid	Sarin
3Î²-Hydroxydeoxydesacetoxy-7-Oxogedunin	dihydrophaseic acid
19-hydroxy-PGB2	Methyl 7-Deshydroxypyrogallin-4-Carboxylate
N-Methylethanolamine phosphate	Kaempferol 3-p-coumarate
quercetagetin 3-methyl ether 7-O-sulfate	O-hexanoyl-adenosine monophosphate
N-Acetylgalactosamine 6-sulfate	Gentisyl alcohol
3-methyl pyruvic acid	3-Methyluridine
SC-51322	9,10-Dihydro-10-(4-hydroxyphenyl)-pyrano[2,3-h]epicatechin-8-one
4-Hydroxy-6-methylpyran-2-one	Apigeniflavan 5-O-xyloside
Oxaloglutarate	Erythronolide B
Gly Met	Thiofanox
2-glyceryl-PGE2	Ginkgolide C
Flutolanil	Lucernol
2'-Deoxyuridine	Uridine
Loureirin D	Gln Cys Asp
Abscisic acid glucose ester	Haematoxylin

Akeboside Std	DG(14:0/18:3(9Z,12Z,15Z)/0:0)
Glycolic acid	2-Hydroxy-1,4-benzoquinone
1,2-Epoxy-3,4-butanediol 4-methanesulfonate	3'-Methyl-2',4',6'-trihydroxydihydrochalcone
Met Thr Trp	3-beta-D-Glucopyranuronosyloxy-5-methylisoxazole
Palmatoside G	Ambanol
Sinapoyltartronate	Citraconic acid dimethyl ester
Labriformidin	Repenol
Allantoin	Adenine
N-Feruloylglycine	5,7,2',4'-Tetrahydroxy-3,6,5'-trimethoxyflavone
Asn Gln Thr	Bruceantinol
quercetin 3-methyl ether	Cys Trp Tyr
12-O-Palmitoyl-16-hydroxyphorbol 13-acetate	2',3'-Cyclic UMP
S-(3-Methylbutanoyl)-dihydrolipoamide-E	1-Caffeoyl-beta-D-glucose
Gly Trp Lys	Benazepril
2,6,3',4'-Tetrahydroxy-2-benzylcoumaranone	2-Nitronaphthalene
4-(Î²-D-Glucosyloxy)benzoate	Phe4Cl-HoPhe-OH
Idebenone Metabolite (Benzenedecanoic acid, 2,5-dihydroxy-3,4-dimethoxy-6-methyl-)	DL-3-Phenyllactic acid
3-Dimethylallyl-4-hydroxymandelic acid	Canavanine
Hydrocotarnine	Tetradecyl sulfate
His Cys	Novaluron
Hydroxyitraconazole	2''-O-Vanilloylvitexin
quercetin 7-xyloside	Verbenalin
quercetin 3-gentiobioside-7-glucoside	Phorate
Atheroline	11-O-Demethyl-7-methoxypradinone II
Jaceidin 5-glucoside	Lactobionic acid
Abu-His-OH	Epoxiconazole
8-Chloroxanthine	3-(3,4-Dihydroxyphenyl)pyruvate
Distemonanthin	Dianthramine
Cimetidine sulfoxide	Met Met Trp
Myricetin 3-(2''-galloylglucoside)	8-oxo capric acid
5-O-Feruloylquinic acid	2-Amino-5-hydroxyl-4-hydroxylamino-6-nitrotoluene
cis-4-Carboxymethylenebut-2-en-4-olide	N-Adenylylanthranilate
Bis-D-fructose 2',1:2,1'-dianhydride	N-Acetylmuramoyl-Ala
4R-hydroxy-octanoic acid	5-Hydroxythiabendazole
Nifuradene	AL-321
Leu Val	(6S)-vitamin D2 6,19-sulfur dioxide adduct / (6S)-ergocalciferol 6,19-sulfur dioxide adduct
Atorvastatin	2,3-dinor Thromboxane B1
4-(Methylnitrosamino)-1-(3-pyridyl)-1-butanol glucuronide	Nicarbazin
Hippurin-1	Î±-D-Xylose 1-phosphate

Barbituric acid, 5-ethyl-5-(2-hydroxyethyl)-	Acyclovir
Deacetylcephalosporin C	Carbidopa
Cys Glu Arg	2,3-dinor, 6-keto-PGF1Î±
Rutaevin	Lamivudine
2,4,5-Trichlorophenoxyacetic Acid, Isooctyl Ester	Oaxacacin
9-Bromo-11beta-hydroxy-16alpha-methylpregn-4-ene-3,20-dione	Ethylnorepinephrine
Diadenosine diphosphate	Eriodictyol 7-O-glucoside
2-Pyridylthioamide	Isoscutellarein
8-C-beta-D-Glucopyranosyldiosmetin 2''-O-rhamnoside	Eriodictyol 3'-O-glucoside
Cadusafos	Trandolaprilat
Arg Cys Gly	Graveoline
Coixinden B	8-C-Methylvelloquercetin 3-methyl ether
Hymexazol	Demethylwedelolactone
Tricrozarin A	Tyr Tyr Asn
Schisantherin A	2,3-dihydro-2-oxo-1H-Benzimidazole-1-propanoic acid
quercetagetin 4'-methyl ether	4'-O-Methylneobavaisoflavone 7-O-(2''-p-coumaroylglucoside)
6-Thioxanthine 5'-monophosphate	Endecaphyllin X
2E,6E,8E,10E-dodecatetraenoic acid	N-(2,4-Dinitrophenyl)-DL-methionine sulfoxide
Homocysteine-penicillamine disulfide	Artonin P
Ethiprole	3-Phospho-D-erythronate
Hydroxyvernolide	Epicalyxin J
Methyl acetoacetate	hellebrigenin
6-Hydroxymyricetin 6,3',5'-trimethyl ether 3-glucoside	D-Ribitol 5-phosphate
1beta,3beta,5alpha,6beta-tetrahydroxyandrostan-17-one	Asn His Cys
dTDP-4-oxo-2,6-dideoxy-D-glucose	beta-D-4-Deoxy-delta4-GlcA-(1->4)-beta-D-Glc-(1->4)-alpha-L-Rha-(1->3)-beta-D-Glc
4-Hydroxy-2-butynal	9S,10S,11R-trihydroxy-12Z,15Z-octadecadienoic acid
Phlorisovalerophenone	Triphenyl phosphate
Glucoalyssin	Duartin, Dimethyl Ether
Proacacipetalin	Dihydroxyacetone Phosphate Acyl Ester
19-Hydroxytetrangulol	Enoximone sulfone
Homocysteinesulfinic acid	Skullcapflavone I
4-(3,5-Diphenylcyclohexyl)phenol	Podophyllotoxin Acetate
Tricyclazole	Mometasone
Sorbitol-6-phosphate	Luteolin 7-(6''-p-coumarylglucoside)
3-Fluoro-1-(4-hydroxyphenyl)-1-propanone	CHIR99021

PE(P-18:0/18:2(9Z,12Z))	(S)-Acetoin
(5-Phenyl-1,2,4-triazol-3-yl)urea	2-Hydroxy-3-carboxy-6-oxo-7-methylocta-2,4-dienoate
Sucralose	Mimosine
7-Hydroxy-5,4'-dimethoxy-8-methylisoflavone 7-O-rhamnoside	5-Hydroxysulfapyridine
Guanosine triphosphate adenosine	2-hydroxy-2-methyl-butyric acid
Met Lys Asp	Arg Phe Tyr
N6,N6-Dimethyladenosine	6-hydroxy-nonanoic acid
Z-Phe-Phe-CHN2	Burimamide
Theogallin	p-cresol
5-Nitro-ortho-anisidine	Calomelanol H
5-Nitrofurfural	Prolylhydroxyproline
Asp Glu Cys	Oxydeprofos
N-Succinyl-L-glutamate	2'-Deoxyuridine
Diethylpropion(metabolite V-glucuronide)	Sufentanil
alpha-Zearalanol	fenbendazole
6-C-Galactosylisoscutellarein	Irisolone
Cyclopiazonic Acid	O-Desacetylcephalothin
R-4-benzyl-3-isobutyryloxazolidin-2-one	Digoxigenin bisdigitoxoside
Formyl-CoA	4-Naphthalimidobutyric Acid
2,4-Dinitrophenol	gibberellin A17
Kaempferol 3-p-coumarate	Novclobiocin 104
Cyclophosphamide	Maleic acid
Temazepam glucuronide	Nitenin
Nithiazide	Monoanhydroescholtzxanthin
Penicillin V	Succinylacetone
Fenirofibrate glucuronide	Protionamide sulfate
2-Caffeoylisocitrate	Dimethyl suberate
CAY10571	Deacetylcephalosporin C
CCCP	Permethrin
Hibiscetin	1-Deoxy-D-altro-heptulose 7-phosphate
Penciclovir	Zalcitabine monophosphate
9-Hydroxyrisperidone	7-Aminocephalosporanic acid
Alamarine	Phe4Cl-Gly-OH
S-(Hydroxyphenylacetothiohydroximoyl)-L-cysteine	(S)-Dihydroorotate
Chlorobenzilate	Beta-Alanyl-CoA
Delsoline	Ala Asp
4-O-beta-D-Glucosyl-sinapate	1 (2)Î±-Epoxydeoxydihydrogedunin
Rabeprazole	(L-Seryl)adenylate
6-Methylthiopurine ribonucleotide	2-Hydroxy-6-oxo-6-(2-hydroxyphenoxy)-hexa-2,4-dienoate

Diphenylhydantoic acid	4-hydroxy-2-oxo-Heptanedioic acid
Mycinamicin VI	b-D-Glucopyranosiduronic acid, 6-(3-oxobutyl)-2-naphthalenyl
luteolin 7,4'-diglucuronide	Diphenylphosphine Acid
3-O-(3,6-Anhydro-alpha-D-galactopyranosyl)-D-galactose 4-O-sulfate	Ureidoglycine
Ethidimuron	HoPhe-HoPhe-OH
6-Hydroxyluteolin 7-[4''-(3-hydroxy-3-methylglutaryl)glucoside]	2-Caffeoylisocitrate
Glu Phe Trp	1-O-Feruloyl-Î²-D-glucose
2,5-Dimethoxycinnamic acid	8-Hydroxyluteolin 7-[6'''-acethylallosyl-(1->2)-3''-acetylglucoside]
1-methyl-N-(9-methyl-9-azabicyclo[3.3.1]non-3-yl)-7-(sulfooxy)-, endo- glucuronide	Cystathionine sulfoxide
3,2'-Dihydroxy-4,4',6'-trimethoxychalcone	Niflumic Acid
GDP-D-glycero-alpha-D-manno-heptose	Parishin C
Fluorodifen	E3040
Glu Cys Trp	N-Glycosyl-L-asparagine
O-Phosphorylethanolamine	Lappaol D
6-gingerol	Riboflavin cyclic-4',5'-phosphate
Pyriftalid	(+)-Bornyl-diphosphate
N-Isopropylterephthalaldehydamide	Quercetin 3-(2'',3'',4''-triacetylgalactoside)
2-Chloro-1,4-naphthoquinone	p-Hydroxyphenytoin glucuronide
8,9,16-trihydroxy palmitic acid	5-O-Methylhoslundin
N-Nitrosofolic acid	Anhydrobrazilic Acid
N-Carboxytocainide glucuronide	Caffeine
Nap-Nap-OH	Penicillamine cysteine disulfide
Chidamide	3-Hydroxysulfolane
threo-3-Hydroxy-D-aspartate	6-Methoxykaempferol 3-(6''-acetylglucoside)
MID47226:6-(3,4-Dihydroxyphenyl)-6a,12b-dihydro-3,10,11,12-tetrahydroxy-[2]benzopyrano[3,4-c]benzopy	Cellohexaose
Malonamide	2-Keto-3-deoxyoctonate (KDO)
N-D-Glucosylarylamine	Gomphrenol
4'-Hydroxychalcone	MID73251:6-[2,3-Dihydroxy-1-(hydroxymethyl)propyl]-1,2-dihydro-7-hydroxy-9-methoxy-cyclopenta[c][1]b
(4S)-4,6-Dihydroxy-2,5-dioxohexanoate	Cladribine
Phe Trp Cys	Mometasone Metabolite (Pregna-1,4-diene-3,20-dione, 9,21-dichloro-6,11,17-trihydroxy-16-methyl-, (6b
Torasemide	Phenyl glucuronide
10-Oxabenzo[def]chrysen-9-one	6'''-(3-Hydroxy-3-methylglutaroyl)isoviolanthin
7,8-Dihydroxycoumarin	Nocodazole

Thymidine	punaglandin 1
b-D-Glucopyranosiduronic acid, 6-(3-oxobutyl)-2-naphthalenyl	Wightin
11beta-Chloromethylestradiol	Alizarin
Isoorientin 7-O-rhamnoside	Pro Cys Gln
L-Isoleucine	Stealthin C
Hydroxytetrabenazine	Flubenzimine
m-Hydroxyphenylpyruvic acid	3,6-dioxo-decanoic acid
CDP-ribitol	Demethylbellidifolin
Ile Ser Arg	Dihydroisomilletenone methyl ether
Novobiocin	(-)-Pinoresinol glucoside
Trp Leu Asp	5-[2-(hydroxymethyl)-5-methylphenoxy]-2,2-dimethyl-Pentanoic acid (Gemfibrozil M4)
GW 848687X	Tetranor-PGEM-d6
3-Phospho-D-erythronate	5-Acetylamino-6-formylamino-3-methyluracil
Ergoline-1,8-dimethanol, 10-methoxy-6-methyl-, (8b)-	Aldohexose 6-phosphate
Flecainide	Guanine
MID42332:(22R)-1Î±,22,25-trihydroxy-26,27-dimethyl-23,24-tetradehydro-20-epivitamin D3 / (22R)-1Î±,22,	N-Oleoyl-L-Serine
p-Hydroxycarvedilol sulfate	Texasin
5'-Butyrylphosphouridine	2-Formylglutarate
Quinalizarin	Idarubicinol aglycone
Fumarprotocetraric Acid	9-hydroperoxy-12,13-dihydroxy-10-octadecenoic acid
Quinazoline acetic acid (3(2H)-Quinazolineacetic acid, 1,4-dihydro-2,4-dioxo-)	PLC thio-PIP2
Lactobionic acid	Hibiscetin
b-D-Glucopyranosiduronic acid, 6-(3-hydroxybutyl)-2-naphthalenyl	Epigallocatechin 5,3',5'-trimethyl ether 3-O-gallate
6-Prenyleriodictyol	Methylnoradrenaline
Medicagol	Tyr Tyr Tyr
Pratensin A	Kalbreclasine
Pyruvic acid	Arg Arg Phe
6Î²-Hydroxytriamcinolone acetonide	Benzo[a]pyrene-7,8-dihydrodiol-9,10-oxide
His Asp	3-Hydroxymethyltriazolopthalazinone
Estrone Benzoate	4-Hexyloxyphenol
Guanosine diphosphate adenosine	Methyloxaloacetate
Piperaduncin B	N2-Methylguanine
Met Met Trp	Nitrazepam
7-Deshydroxypyrogallin-4-Carboxylic Acid	5,6,3',5'-Tetrahydroxy-3,7,8,4'-tetramethoxyflavone

Arbutin 6-phosphate	8-C-beta-D-Glucopyranosyldiosmetin 2"-O-rhamnoside
Pro Trp Gly	Orsellinic acid
2-Naphthalenepropanol, 6-methoxy-a-methyl-, hydrogen sulfate	Uroporphyrin I
Mesquitol-4beta-ol 3,8-dimethyl ether	12-Methoxy-4,4-Bisnor-5Î±-8,11,13-Podocarpatrien-3-Ol
Methylmalonic acid	Cys Glu Lys
5-O-Methylvisamminol	Gentisin
N-[(diphenylmethoxy)acetyl]-Glutamine	4-O-beta-D-Glucosyl-sinapate
1'-Acetoxyeugenol acetate	Pro Glu Asn
Penicillamine disulfide	N-Acetyl-D-glucosamine 1,6-bisphosphate
4-oxo-nonenal	5'-S-Methyl-5'-thioinosine
Thioquinox	Trovafloxacin
Cuminaldehyde	3-hydroxy-cis,cis-muconic acid
2'-Hydroxy-5',6'-dimethoxy-3,4-methylenedioxyfurano[2",3":4',3'] dihydrochalcone	Medicagol
4-hydroxy-valeric acid	Xanthosine
5-Hydroxy-7,3',4',5'-tetramethoxyflavanone	Quercetin 3'-isobutyrate
Epibatidine	4-Hydroxy-3-nitrosobenzamide
Rhizocticin C	Benomyl
5-Sulfosalicylic acid	Homocysteinesulfinic acid
Dolichyl phosphate D-mannose	Trp Asn Gly
3-Dimethylallyl-4-hydroxybenzaldehyde	His Lys Val
dTMP	N1-(2-Hydroxyethyl)flurazepam
Ala Asp	Aramite
MID42473:1Î±,25-dihydroxy-26,26,26,27,27,27-hexafluoro-16,17,23,23,24,24-hexadehydro-19-norvitamin D3	Villosone
Flumioxazin	2-Hydroxytacrine
Pyridoxamine	Lamioside
Ser Arg Tyr	Cibarian
D-glycero-D-manno-Heptose 7-phosphate	Azaserine
N-Acetylaspartylglutamic Acid	Volkenin
Gemichalcone B	PE(16:0/0:0)
Succinoadenosine	2-N,6-N-Bis(2,3-dihydroxybenzoyl)-L-lysine amide
ensulizole	Arg Lys
Gly Trp Gly	Flecainide
Ginkgolide A	2,4-Dichlorophenoxybutyric Acid, Methyl Ester
Repenol	7-Nitroindazole

8-oxo-nonanoic acid	5-Hydroxy-7,8-dimethoxyflavanone 5-rhamnoside
Chloropropylate	2-Aminoadenosine
Thr Ile Thr	L-alpha-Aspartyl-L-hydroxyproline
3,4-Dimethylbenzoic acid	2-Methyl-1-nitroanthraquinone
2,8-Dihydroxy-3,4,9,10-tetramethoxypterocarpan	Dalpanol O-glucoside
7-Hydroxymethyl-12-methylbenz[a]anthracene sulfate	Levan
Cephalosporin C	Quercetin 3-(6"-malonylglucoside)-7-rhamnoside
Gentisyl alcohol	3-Methyleneoxindole
1,1,1-Trichloro-2,2-bis(4-hydroxyphenyl)ethane	Lupinalbin A
4'-Chloroaurone	4'-O-beta-D-Glucosyl-cis-p-coumarate
N,N-Bis(2-chloroethyl)-DL-alanine	Hymexazol
Prolylhydroxyproline	Sertraline Carbamic acid
Chalconaringenin 2'-O-glucoside 4'-O-gentobioside	cis-1,2-Dihydroxy-1,2-dihydrodibenzothiophene
Glafenine	2-Pyrimidin-2-yl-Propionic Acid
Harzianopyridone	5-Hydroxypyrazinoic acid
Oxalosuccinic acid	Î²-Phenylalanoyl-CoA
3,4,2',4',6',beta-Hexahydroxychalcone 2'-glucoside	PGF1Î²
4-Phthalimidoglutaramic acid	Rhodocladonic Acid
11-bromo-undecanoic acid	4-[[5-(acetylamino)-1H-indol-3-yl]methyl]-3-methoxy-N-[(2-methylphenyl)sulfonyl]-Benzamide
Asn Gly Gly	Iprobenfos
Sulisobenzone	Cadusafos
5,6,2',3',4'-Pentamethoxyflavone	Thr Ser Asn
apigenin 7-sulfate	Met Phe
tert-butyl p-(bromomethyl) Benzoate	Resorcinolnaphthalein
Tyrphostin B44 (-)	Hydroxynicorandil
apigenin 7-glucuronide-4'-rhamnoside	Isoetin 7,2',4',5'-tetramethyl ether
N-Acetylglucosamine 6-phosphate	2-cyano-Pyrimidine
Isofurcatain 7-O-glucoside	2,3,5-Trihydroxytoluene
Azomycin	4-Deoxy-beta-D-gluc-4-enuronosyl-(1,3)-N-acetyl-D-galactosamine 4-sulfate
Baileyin	dihydrodigoxin
PGF1Î²	2-Furylmercury chloride
Coronopilin	Debromohymenialdisine
(E)-4-stilbenol	Acitretin Ro 23-4750
Deazaflavin	2-keto valeric acid

Isorhamnetin 3-glucosyl-(1->2)-galactoside-7-glucoside	Asp-Val-OH
4-Methylumbelliferyl Î²-D-glucuronide	all-trans-heptaprenyl diphosphate
Indole-3-acetaldoxime N-oxide	13,14-dihydro-16,16-difluoro Prostaglandin E1
Desmethylranitidine	(2R)-5,7,3',4'-Tetrahydroxyflavanone 6-C-glucoside
Hydroxydiphenoxylic acid(HDPA)	His Ile Tyr
Met Glu Gln	Piretanide
Cucurbitacin F	Trp His Ala
Diguanosine triphosphate	Cyanthoate
Clobetasol propionate	Sinapic acid
(3S)-7-hydroxy-2',3',4',5',8-pentamethoxyisoflavan	Thr Ser
5-O-Methylembelin	1,4-DPCA
Î³-hexenoic acid	4-Hydroxy-L-glutamic acid
5-(alpha-Phenylethyl)semioxamazide	L-Oleandrosyl-oleandolide
3-o-Ethyl-L-ascorbic acid	13Z-octadecenoic acid
methyl 11-(3,5-epidioxy-2-ethyl-cyclopentyl)-9-hydroperoxy-10-undecenoate	Actinorhodin
Procarbazine	Fraxin
Prostaglandin E2-biotin	Norathyriol
8-oxo capric acid	5,7,8,3',4'-Pentahydroxy-3,6-dimethoxyflavone
ent-Fisetinidol-(4beta->8)-catchin-(6->4beta)-ent-fisetinidol	Metiamide
Asn-Trp-OH	Pinocembrin 7-O-neohesperidoside 2'''-O-acetate
Fludrocortisone	Steroid O-sulfate
Meperidine N-oxide	2-carboxylic acid derivative of Oxamniquine
12a-Hydroxy-9-Demethylmunduserone-8-Carboxylic Acid	Mitomycin
Thiarubrine A	(S)-3-(Imidazol-5-yl)lactate
Scutellarein 6-glucoside	LPA(P-16:0e/0:0)
PS(14:1(9Z)/14:0)	9,13-dihydroxy-10-ethoxy-11-octadecenoic acid
Sinalbin	1,2-Dihydroxymint lactone
Gentisin	2,3-dinor-11b-PGF2Î±
1-(3-Carboxypropyl)-3,7-dimethylxanthine	6-Hydroxyluteolin 6-sulfate
Demethylwedelolactone	3-Thiatetradecanoic Acid
Laurolitsine	Myricetin 3'-glucoside
Ikarisoside D	Pyruvic acid
Toxoflavine	Bensulfuron-methyl
Cinchonain Id	Eupachloroxin
Sarisan	Propane-1,2-diol 1-phosphate
2-Methylcitric acid	Quercetagetin 7-methyl ether

Hesperetin 7-O-glucoside	Sulbenicillin
L-Arabinono-1,4-lactone	Tyr Pro Pro
5,7,3',4',5'-Pentahydroxy-3,6-dimethoxyflavone	7,8-Dihydroneopterin
Leukotriene D4	Nicofetamide
6-Demethylsterigmatocystin	Dimefuron
Ile Met Met	Hydroxyibuprofen
Methyl-2-alpha-L-fucopyranosyl-beta-D-galactoside	12S-acetoxy-punaglandin 2
PA(13:0/0:0)	3-methoxy Prostaglandin F1Î±
Aztreonam	6-Hydroxyluteolin 6-glucuronide
Cefamandole	3,5,7,2',5'-Pentahydroxyflavone
2,4-Dioxotetrahydropyrimidine D-ribonucleotide	Folic acid
3-Hydroxy-7-aminonitrazepam	Cycloguanil
Distemonatin	3,4-dihydroxymandelate
N-Succinyl-2-amino-6-oxopimelate	N-Glycolyl-D-mannosamine 6-phosphate
4-Hydroxyphenylglyoxylate	Hydroxyvernolide
Methylthiobenzoic acid	4-hydroxy-valeric acid
Selinidin	Captopril
Albizziine	MID42396:26,27-diethyl-1Î±,25-dihydroxy-22-thia-20-epivitamin D3 / 26,27-diethyl-1Î±,25-dihydroxy-22-t
2-Propylsuccinic acid	Kaempferol 3-(6G-malonylneohesperidoside)
p-Benzosemiquinone	8-C-alpha-L-Arabinosylluteolin
Dubinidine	2-Hydroxy-6-oxo-6-phenylhexa-2,4-dienoate
Gomphrenol	Deaminohydroxyblasticidin S
Fortimicin KK1	Scutellarein 4'-methyl ether 7-(2",6"-diacetylalloside)
L-Galactono-1,4-lactone	1-Phenyl-5-mercaptotetrazole
Olsalazine	4-(2-hydroxypropoxy)-3,5-dimethyl-Phenol
Adenophostin B	Gossypetin 7-methyl ether 8-acetate
Phenylmercury acetate	Phenol
1-Phosphatidyl-1D-myo-inositol 3-phosphate	Tomentin 6-galactoside
2-Hydroxybutane-1,2,3-tricarboxylate	Catechin 5-O-beta-D-glucopyranoside
Estradiol disulfate	Ethyl butyrylacetate
Hydroxyphthioceranic acid (C48)	Tefluthrin
2-Methyl-3-oxopropanoic acid	Temazepam glucuronide
Guanidinosuccinic Acid	N-Ac-Tyr-Val-Ala-Asp-CMK
Hydroxyibuprofen	5-Azacytidine 5'-monophosphate
8-cinnamoyl-3,4-dihydro-5,7-dihydroxy-4-phenylcoumarin	PS(21:0/0:0)
D(-)-Î²-hydroxy butyric acid	Nifurtimox
D-myo-Inositol-1,5-diphosphate	Euparotin
Flemiwallichin D	Longicaudatin

Lansoprazole	Desacetylcefotaxime lactone
Bis(glutathionyl)spermine disulfide	PE(P-20:0/21:0)
Duartin (-)	Herbacetin 8-(2",3",4"-triacetylxyloside)
PI(18:0/18:2(9Z,12Z))	N-Acetyl-9-O-lactoylneuraminic acid
MID42413:1Î±,25-dihydroxy-26,27-dimethyl-22,22,23,23-tetradehydro-24a,24b-dihomo-20-epivitamin D3 / 1	6,8-dihydroxy-octanoic acid
17beta-(Acetylthio)estra-1,3,5(10)-trien-3-ol acetate	Narciclasine
4-Hydroxytrazodone	DHAP(8:0)
Avermectin A1b monosaccharide	Tyr-Met-OH
3-tert-Butyl-5-methylcatchol	Stemonone
4-p-coumaroylquinic acid	Lyxosylamine
2H-1-Benzopyran-6-acetic acid, 7-hydroxy-8-methoxy-2-oxo-	Canavaninosuccinate
Monoethyl phthalate	Ptd(S)Ins-(3,4)-P2 (1,2-dioctanoyl)
punaglandin 1	3,5-Dinitro-L-tyrosine
2-O-[2-O-(alpha-D-Mannopyranosyl)-alpha-D-glucopyranosyl]-3-phospho-D-glycerate	Dinor-PGE2
Fosthiazate	Isovaleric acid
Madecassic Acid	2-(p-Methoxyphenyl)-3-(m-chlorophenyl)acrylonitrile
3-beta-D-Glucopyranuronosyloxy-5-methylisoxazole	Barbatoflavan
3,5,7-Tris(acetyloxy)-2-[4-(acetyloxy)-3-hydroxyphenyl]-4H-1-benzopyran-4-one	Tazobactam
Isoprothiolane sulfoxide	His Cys Gly
Isoorientin 2"-O-(E)-caffeate	Okanin 4-methyl ether 4'-glucoside
Gly-Gly-OH	7-Oxotetradecanoic acid
Idebenone Metabolite (Benzenebutanoic acid, 2,5-dihydroxy-3,4-dimethoxy-6-methyl-)	Phenyl acetate
2(Î±-D-Mannosyl)-D-glycerate	3,4,5,2',4',6'-Hexahydroxychalcone 2'-glucoside
5-Amino-6-(5'-phosphoribitylamino)uracil	Maximaisoflavone B
5-(3,4-Diacetoxybut-1-ynyl)-2,2'-bithiophene	Monoisobutyl phthalic acid
Pitrazepin	5,7,3',4',5'-Pentahydroxy-3,6-dimethoxyflavone
Teniposide	Nap-Ala-OH
AMPA	Patellamide A
Asn-Nap-OH	Colnelenic acid
N-Acetyl-L-Histidine	L-Tyrosine, 3-methoxy-a-methyl-, hydrogen sulfate
AG-1478	Capsazepine
Ala-Ser-OH	Gyrophoric Acid

Hecogenin Acetate	MID47226:6-(3,4-Dihydroxyphenyl)-6a,12b-dihydro-3,10,11,12-tetrahydroxy-[2]benzopyrano[3,4-c]benzopy
Nizatidine	Leu Val
3-Methyleneoxindole	5-Acetoxypalisadin B
3,6-dioxo-decanoic acid	Erioflorin acetate
Hydroxyphthioceranic acid (C37)	Orientin 2",6"-diacetate
PE(19:0/20:3(8Z,11Z,14Z))	2',Î²-Dihydroxychalcone
2',4',6'-Trimethoxy-3,4-methylenedioxydihydrochalcone	1-amino-3,3-diethoxypropane
Val-Val-OH	Luteolin 7,3'-dimethyl ether 4'-glucoside
Flaccidin B	Neoduleen
Piceid	Glu Asn Met
9,13-dihydroxy-10-ethoxy-11-octadecenoic acid	8-Hydroxyluteolin 4'-methyl ether 7-(6'''-acetylallosyl)(1->2)(6"-acetylglucoside)
JWH 081	Pongamoside A
8-Oxo-dGMP	Pyridoxine phosphate
Methylthioribosyl phosphate	2-methyl-dodecanedioic acid
Vanillyl alcohol	5'-Oxoinosine
L-Leucine	Dihydropicromycin
Euchrenone b2	Tetracenomycin C
3'-Demethylstaurosporine	(-)-Amurensisin
luteolin 7-glucuronosyl-(1->2)-glucuronide-4'-glucuronide	7-Piperazin-1-yl-thieno[2,3-c] Pyridine
p-Cresol glucuronide	Propaphos
8-Hydroxyluteolin 7-glucoside	2,4-Dihydroxytacrine
Dimethyl suberate	Triflumizole
dTDP-2,6-dideoxy-D-glycero-hex-2-enos-4-ulose	Akeboside Std
Chalconaringenin 2'-rhamnosyl-(1->4)-xyloside	Orotidine
Diflubenzuron	p-Hydroxyphenobarbital glucuronide
Fludioxonil	9S-hydroxy-10S,11S-epoxy-12Z,15Z-octadecadienoic acid
Tyr Ser Tyr	Decaketide tricyclic intermediate
Phe4Cl-Asp-OH	CMN 131
Platycarpanetin 7-O-laminaribioside	Dolichyl b-D-glucosyl phosphate
Trinexapac-ethyl	Wy 14643
3-keto-n-caproic acid	cortisol 21-sulfate
Glutinosone	Amidosulfuron
Thiodicarb	Quercetin 3-methyl ether
Xamoterol	Manniflavanone
b-D-Glucopyranuronic acid, 1-(6-methoxy-2-naphthaleneacetate)	Cucumerin A
Flutamide	Knipholone

7-Hydroxymethotrexate	Caracurine V
Ethynylestradiol 3-sulfate	(6S,9R)-Vomifoliol
Kaempferol 3-isorhamninoside-7-rhamnoside	Fludioxonil
3-(3,4-Dihydroxyphenyl)pyruvate	Flemiwallichin D
5,7,4'-Trihydroxyflavanone 7-sulfate	Pollenitin 8-butyrate
PA(14:1(9Z)/0:0)	4-Fluorophenylacetic acid
Arg Cys Ser	14S-hydroxy-hexadecanoic acid
Oil Orange SS	Penicillin V
6-Hydroxymyricetin 3,6,3',5'-tetramethyl ether 7-glucoside	1-Nitropyrene
Imidacloprid	Isoniazid alpha-ketoglutaric acid
3,5-Dinitro-L-tyrosine	Penicillamine disulfide
Soularubinone	TyrMe-Tyr-OH
Dexchlorpheniramine	Gallocatechin 3-O-gallate
6-Methylthioguanosine monophosphate	Ile Arg Ile
Barbiturate	Tamarixetin 5-glucoside-7-glucuronide
Gly Gly Phe	Cycloartomunoxanthone
1,5-Naphthalene diisocyanate	Furmecyclox
quinoxaline-2-carboxylic acid	12-Tridecynoic acid
6-O-Methylguanine	Compactin diol lactone
C22-OH Sulfatide	Gln Tyr Cys
4-Methylthiobutyl-desulfoglucosinolate	Senkirkine
Ophiopogonin D	Pydanon
Thyrotropin releasing hormone	Dihydrojasmonic Acid, Methyl Ester
Asp Asp Ser	Î±-Thiophenecarboxylic acid
Artoindonesianin B	1,8-Naphthyridine-3-carboxylic acid, 1-ethyl-1,4-dihydro-7-hydroxy-4-oxo-
Dipropyl disulfide	Phe4Cl-His-OH
Glutathionylspermidine	6,8-Di-C-beta-D-arabinopyranosylapigenin
L-Oleandrosyl-oleandolide	Hydroxyphthioceranic acid (C37)
4-Chloro-17alpha-methyl-17beta-hydroxy-4-androsten-3-one	Indoleamine
Magnesium protoporphyrin monomethyl ester	Tamarixetin 3-O-sulfate
Texasin	11-bromo-undecanoic acid
(R)-(Homo)2-citrate	4'-Hydroxyflurbiprofen
Val Ile Asp	PE(22:5(7Z,10Z,13Z,16Z,19Z)/24:0)
Demeclocycline	p-Hydroxytiaprofenic acid
11-O-Demethyl-17-O-deacetylvindoline	Robinetinidol-(4alpha->8)-catechin-(6->4alpha)-robinetinidol
5,6-Dihydroxy-7,8,4'-trimethoxyflavanone	5,6-Dimethylbenzimidazole
AG-123	N2-Succinylglutamic acid
5'-Hydroxysulfapyridine glucuronide	Laricitrin 3-(6"-acetylglucoside)
Hypusine	Phosacetim

D-4'-Phosphopantothenate	S-Succinyldihydrolipoamide
Asp Pro	6,8-Di-C-methylkaempferol 7-methyl ether
alpha-L-Rhamnopyranosyl-(1->2)-beta-D-galactopyranosyl-(1->2)-beta-D-glucuronopyranoside	Suprofen Methyl Ester
Met-Abu-OH	Pyrazosulfuron
Polydine	UDP-D-galacturonate
Tephrosol	7-oxo-11E,13-Tetradecadienoic acid
7-Mercaptoheptanoic acid	Cedrediprenone
beta-Cymaropyranose	Zineb
Aesculin	Bupivacaine
Cytarabine	Syringetin
Myricetin 3-O-(4"-O-acetyl-2"-O-galloyl)-alpha-L-rhamnopyranoside	Euchrenone a5
Leu-Nap-OH	1-Naphthalenesulfonic acid
Chloranocryl	Abu-Met-OH
Pentamidine	1,2-Dihydroxy-3-keto-5-methylthiopentene
Ile Ala	Gly-His-OH
Asp Arg Cys	Pedunculagin
Met Asp Gly	5-amino-1-[3,4-dihydroxy-5-(hydroxymethyl)oxolan-2-yl]imidazole-4-carboxamide
Scopoline	Dehydroascorbic acid
3-(3,5-Diiodo-4-hydroxyphenyl)lactate	L-Galactono-1,4-lactone
CAY10597	PG(14:1(9Z)/14:1(9Z))
5S-HETE di-endoperoxide	Fosfosal
2,3-Dinor-TXB2	N-Succinyl-L-glutamate
Alpha-D-Fucose	Platycarpanetin 7-O-laminaribioside
Met Gln Tyr	U-0126
4-Fluorocyclohexadiene-cis,cis-1,2-diol	Baicalein 6-methyl ether 7-glucuronide
Sophoramine	Monodehydroascorbate
Phe Thr Phe	Formimidoyl-fortimicin A
Thr Tyr	Ile Ser Arg
Neosaxitoxin	Isorhamnetin 3-(4''',6'''-diacetylglucosyl) (1->3)-galactoside
Anatoxin a(s)	Chicoric acid
CMP-N-acetylneuraminic acid	2-Oxo-6-methylthiohexanoic acid
9S,10S,11R-trihydroxy-12Z,15Z-octadecadienoic acid	PE(19:0/0:0)
2-Hydroxy-3-(4-methoxyethylphenoxy)-propanoic acid	Neodunol
LY364947	Sebacic acid
Psicofuranine	Kaempferol 3-(3"-p-coumaryl-6"-ferulylglucoside)

9,10-epoxy-13-oxo-11-octadecenoic acid	Dehydroamlodipine
Halfordinol	GDP-D-glycero-alpha-D-manno-heptose
2-Dehydro-D-xylonate	Guanosine diphosphate adenosine
3-Methoxy-4-Hydroxyphenylglycol Sulfate	Trp Tyr Tyr
Arg Arg Asn	Rehmaionoside B
2',3'-Cyclic adenosine monophosphate	O-Carbamoyl-deacetylcephalosporin C
alpha-Hederin	Quercetin 3-isobutyrate
Succinic anhydride	Vamidothion
Glucoerucin	11beta-Chloromethylestradiol
Sulfisoxazole	19(R)-hydroxy-PGF1Î±
4-Hydroxy-3-methylbenzoic acid	Quisqualic acid
Met His Cys	Vomicine
Phosphinothricin	Anthemis glycoside A
5-O-Caffeoylshikimic acid	Chrysoeriol 7,4'-diglucuronide
Orellanine	Isoorientin 2'',6''-diacetate
Cys Pro	9,13-octadecadiynoic acid
Griseofulvin	10-Oxabenzo[def]chrysen-9-one
GW 590735	5-Aminoimidazole-4-carboxamide-1-Î²-D-ribofuranosyl 5'-monophosphate
3,4-Dihydroxyphthalate	3-Hydroxy-L-tyrosyl-AMP
Demeton-S-methylsulphon	His Asn Cys
Hinokiflavone	Mulberrofuran C
3-(2'-Methylthio)ethylmalic acid	Patulin
Cerosilin B	Sulindac sulfone
Cyclochlorotine	Calomelanol D-1
GlcCer(d18:0/22:0)	dTDP-3-methyl-4-oxo-2,6-dideoxy-L-glucose
N-Ethylmaleimide-S-glutathione	Palmatoside G
Specionin	Clavamycin A
(-)-Acanthocarpan	TWS119
3b,16b-Dihydroxyandrostenone sulfate	5-Phosphonooxy-L-lysine
Triamcinolone	Leu Leu Pro
3-hydroxy-suberic acid	Galactomannan
Propionylglycine	Nalbuphine-6-sulfate
Lappaol D	Tetrahydroxypteridine
TyrMe-Met-OH	cis-4,5-Dihydroxycyclohexa-1(6),2-diene-1,2-dicarboxylate
Trp Lys Glu	Glycoperine
3-hydroxy-tetradecanedioic acid	Tyr Arg Phe
fosphenytoin	5-(3-Hydroxy-4-acetoxybut-1-ynyl)-2,2'-bithiophene
Flumazenil acid	Alphitonin
JWH 073 5-hydroxyindole metabolite	6-Hydroxyluteolin 5,6,3',4'-tetramethyl eter 7-cellobioside

Pro Arg Ile	Nimesulide
Avenanthramide A	butalbital
9a-Fluoroallotetrahydrocortisol	Gambirtannine
4-Deoxy-beta-D-gluc-4-enuronosyl-(1,3)-N-acetyl-D-galactosamine 4-sulfate	3-[(4-Carboxy-4-methylpentyl)oxy]-4-methylbenzoic acid (Gemfibrozil M3)
CCG-1423	Isopenicillin N
(2R,4R)-tert-butyl 4-(hydroxymethyl)-2-phenylthiazolidine-3-carboxylate	Nemertelline
Sertraline	Laurencione diacetate
6-(N-Acetyl-alpha-D-glucosaminyl)-1-phosphatidyl-1D-myo-inositol	(Â±)9-HpODE
Asn His Gln	(R)-4'-Hydroxy-3,4-dimethoxydalbergione
Laurinterol	11-Hydroxyiridodial glucoside pentaacetate
albendazole sulfoxide	Dibutyl succinate
floxuridine	Purine mononucleotide
3-[(4-Carboxy-4-methylpentyl)oxy]-4-methylbenzoic acid (Gemfibrozil M3)	Deoxyadenosine monophosphate
Tetracenomycin B3	Thr-Phe4Cl-OH
Trp Cys Glu	2-(4'-Methylthio)butylmalic acid
Gingerdione	3-Dehydroquinic acid
Mazindol	CAY10535
Phosacetim	E3040 sulfate
Quizalofop-P-tefuryl	Met Ser Asn
Robinobiose	Undecanedioic acid
Lumazine	Val-Abu-OH
17Î±,20Î²-Hydroxyprogesterone sulfate	3-Methoxy-4-hydroxyphenylglycol glucuronide
Lupinisol B	Gly Thr Cys
monodesmethyl chlorpheniramine	Apigenin 7-(6'''-acetylallosyl-(1->2)glucoside)
Glu Phe Met	Caohuoside D
cis-4,5-Dihydroxycyclohexa-1(6),2-diene-1,2-dicarboxylate	Silafluofen
Endothal	(Â±)-Mevalonolactone
Eupalitin 3-galactoside	Dihydroartemisinin
Succinylproline	P1,P4-Bis(5'-adenosyl) tetraphosphate (ApppppA)
8-Hydroxyluteolin 4'-methyl ether 8-glucoside-3'-sulfate	7H-Dibenzo[c,g]carbazole
Candimine	Thr Arg Cys
Coriamyrtin	Trp Glu
MID42012:24-(dimethoxyphosphoryl)-25,26,27-trinorvitamin D3 / 24-(dimethoxyphosphoryl)-25,26,27-trin	DG(21:0/22:1(13Z)/0:0)[iso2]
9alpha-Fluoro-11beta,16alpha,17alpha,21-tetrahydroxypregn-4-ene-3,20-dione	Neocarzinostatin chromophore

dTDP-L-olivose	Mometasone Metabolite (Pregna-1,4-diene-3,20-dione, 9,21-dichloro-6,11,17-trihydroxy-16-methyl-, (6a
Benzimidazole	p-Hydroxybenzyldesulphoglucosinolate
Paeonilactone B	Quercetin 3-sophoroside-7-glucuronide
Val-Thr-OH	NAc-DNP-Cys
9,10-epoxy-11-hydroxy-12-octadecenoic acid	1-O,2-O,6-O-Trigalloyl-beta-D-glucose
1-Acetoxypinoresinol	2-(2'-Methylthio)ethylmalic acid
5beta-scymnol	5-undecenoic acid
Diadenosine triphosphate	Anatibant
2-Imino-3-(7-chloroindol-3-yl)propanoate	N-Dealkylzuclopenthixol sulfoxide
Tyr Phe Arg	7-Hydroxymethyl-12-methylbenz[a]anthracene sulfate
Cys Arg Asp	3-Ketosucrose
4-(2-hydroxypropoxy)-3,5-dimethyl-phenol	DL-Methionine sulfoxide
4-Dedimethylamine-4-oxo-anhydro-7-Cl-tetracycline	VER-50589
Dantrolene	3-Methylindole
Inosinic acid	4-Methyl-5-thiazoleethanol
p-Nitroglutethimide	5,6-Dihydroxy-7,8,4'-trimethoxyflavanone
Arg Ser Cys	Dihydroxytrazodone
Neu5AcÎ±2-3GalÎ²1-4GlcNAcÎ²-Sp	Lys-Lys-OH
2-Thiopheneacrylic acid	Fenthion
His Ala Gly	Monomethyl glutaric acid
Pro Gly Ser	Phenothrin
Cyclic CMP	TRIM
PI(O-16:0/17:2(9Z,12Z))	5-(3,4-Dihydroxyphenyl)-5-ethylbarbituric acid
WIN56291	p-Acetamidophenyl glucuronide
p-(3,4-Dihydro-6-methoxy-2-naphthyl)phenol	Scutellarein 6-glucoside
3,5-Dinitrosalicylic acid	Cyclo(deltaAla-L-Val)
Benzo[a]pyrene-cis-9,10-dihydrodiol	Flavonol 3-O-beta-D-glucosyl-(1->2)-beta-D-glucosyl-(1->2)-beta-D-glucoside
HMT-toxin	Isoscutellarein 4'-methyl ether 8-(6''-n-butylglucuronide)
Isradipine Metabolite (3,5-Pyridinedicarboxylic acid, 4-(2,1,3-benzoxadiazol-4-yl)-2,6-dimethyl-, mo	4-pentynoyl-Coenzyme A
10-Hydroxydesipramine	1,8-ANS
6-(alpha-D-Glucosaminyl)-1D-myo-inositol	CAY10589
Chlorfensulphide	Metaldehyde
4-Nitrophenol	Isradipine Metabolite (3,5-Pyridinedicarboxylic acid, 4-(2,1,3-benzoxadiazol-4-yl)-1,4-dihydro-2,6-d

Aciculatin	Microlenin
IDFP	2,3-dihydroxy-3-methylbutyric acid
5-(4-Hydroxybut-1-ynyl)-2,2'-bithiophene	Abu-Thr-OH
Cyazofamid	GW 590735
Vomicine	Mevinphos
(Â±)9-HpODE	Prazepam
2-Cyanopyridine	2',4'-Dihydroxy-3'-isovaleryloxy-6'-methoxychalcone
Elephantorrhizol	Hectochlorin
WIN54954	Dinor-PGD2
(S)-dihydrolipoic acid	Dyphylline
3-(4-Hydroxyphenyl)propionic acid	Jaceidin 4'-glucuronide
Carboxyibuprofen	Sudan I
Norstictic Acid	alpha-L-Rhamnopyranosyl-(1->2)-beta-D-galactopyranosyl-(1->2)-beta-D-glucuronopyranoside
Swertianin	D-Lombricine
Tyr Asp Tyr	PA(15:1(9Z)/0:0)
E3040 sulfate	p-Cresol glucuronide
Ximaosteroid D	Acenaphthenequinone
quercetin 3-(2''-acetylgalactoside)	2,3,4'-Trihydroxy-4-Methoxybenzophenone
Glu His Cys	Taccalonolide A
Spinasaponin A	Formononetin 7-O-(6''-acetylglcoside)
Patuletin 3-glucoside-7-sulfate	2S-hydroxylauric acid
Chaparrinone	Tricyclazole
(R)-2,3-Dihydroxy-3-methylpentanoate	Lys Met Met
3-Methoxymandelic acid-4-O-sulfate	Chloramphenicol Monoglucuronide
Scriptaid	Cys Tyr
(Â±)-Goniothalesdiol	Epigallocatechin 3-O-caffeate
Dothistromin	O-Feruloylgalactarate
Cys Trp Tyr	Usambarensine
Traumatic Acid	2-cis,6-trans-farnesyl diphosphate
5-trans Fluprostenol	Thymonin
7-Methoxy-2-methylisoflavone	2,3-dinor-PGE1
3-(3-Methylbutyl)tricetin 5-neohesperidoside	Dipyrocetyl
Dimefox	MK 571
D-Lysine	Limocitrol 3-glucoside
2,2-Dimethyl-3-(4-methoxyphenyl)-4-propyl-2H-1-benzopyran-7-ol acetate	Cephamycin C
MID42020:26,26,26,27,27,27-hexafluoro-1Î±,25-dihydroxy-23,23,24,24-tetradehydrovitamin D3 / 26,26,26,	Quercetin 3-(2''',3''',5'''-triacetyl-alpha-L-arabinofuranosyl)(1->6)-glucoside
4-Hydroxymethyl-3-methoxyphenoxyacetic acid	2,6-Diamino-4-hydroxy-5-N-methylformamidopyrimidine

Met Pro Gly	Swietenolide-3-Acetate
MCPA-thioethyl	Inosine
Meclofenoxate	(4S)-4,6-Dihydroxy-2,5-dioxohexanoate
bis(4-fluorophenyl)-Methanone	8E,10E-Dodecadienyl acetate
Gly-Nap-OH	3-Hydroxysuberic acid
Latanoprost Lactol	3,5,7-Trihydroxy-8-methoxy-4'-prenyloxyflavone
Neohesperidin Dihydrochalcone	S-(N-Hydroxy-N-methylcarbamoyl)glutathione
Benzyl thiocyanate	BML-190
C75	Dillenetin
Cryptopleurine	methyl 9,15-dihydroperoxy-10E,12Z,16E-octadecatrienoate
4-hydroxy enanthoic acid	alpha-Zearalanol
Prephenic acid	Traumatic Acid
(S)-Acetoin	Isradipine Metabolite (2,1,3-Benzoxadiazole, 3,5-pyridinedicarboxylic acid deriv.)
WWL70	5-Hydroxydantrolene
Carprofen	7-methyl-3-oxooctanoic acid
gibberellin A3 O-beta-D-glucoside	LysoPE(18:3(6Z,9Z,12Z)/0:0)
His Met	AG-490
LacCer(d18:0/22:0)	3-(3,5-Diiodo-4-hydroxyphenyl)lactate
5-(3,4-Dihydroxyphenyl)-5-ethylbarbituric acid	Dicrotophos
Lupinalbin A	Indospicine
Sinapic acid	2-octenal
2,4,6,8,10-dodecapentaenal	Adouetine Z
Asp Glu Asn	Hydrosorbic acid
N1-(5-Phospho-D-ribosyl)-AMP	Brianthein X
Propofol glucuronide	8-Epideoxyloganin tetraacetate
Wharangin	p-Benzosemiquinone
Dyphylline	Vanilpyruvic acid
Isochamaejasmin	Dubiusine
Ser Gln Gly	11-O-Demethylpradinone I
6-Chloroguanine	Flutamide
Clomeprop	Isophylloflavanine
Evasterioside B	N-1-Desalkylflurazepam
7-methyl-3-oxooctanoic acid	Licorice glycoside C2
Acevaltrate	Naringenin 7-O-(2",6"-di-O-alpha-rhamnopyranosyl)-beta-glucopyranoside
Patulin	Triacetin
5,7,3'-Trihydroxy-6,4',5'-trimethoxyflavanone	DAF-2
Erythronolide B	Furconazole-cis
Leonurine	Vicianose
Papyriferic acid	Disulfoton

Barbamide	7-oxo-11Z-Tetradecenoic acid
Cercosporin	Pterine
Ala Phe Phe	epi-4'-hydroxyjasmonic acid
U-0126	Gly Lys Cys
N2,N5-Dibenzoyl-L-ornithine	N-Butyl-1H-pyrazolo[3,4-d]pyrimidin-4-amine
(1R,6R)-6-Hydroxy-2-succinylcyclohexa-2,4-diene-1-carboxylate	Benzyl nicotinate
Idebenone Metabolite (QS-4)	Glaucarubinone
Kaempferol 3-(2G-glucosylrutinoside)	N-Desmethylketazolam
Ala Glu Ala	Griseofulvic Acid
Propachlor	Panaxytriol
5-Hydroxy-7,8-dimethoxyflavanone 5-rhamnoside	beta-Alaninebetaine
Pilosanol C	D-Biopterin
Hydrosorbic acid	Swertianin
Syringin	Firocoxib
20,21,21-Trifluoro-3-methoxy-19-nor-17alpha-pregna-1,3,5(10),20-tetraen-17-ol	(R)-Roscovitine
Bolusanthin	Gly Met Met
Lamiide	Alectrol
Flumazenil	Paeonoside
Dinor-PGE2	2-hydroxy-decanedioic acid
Kinamycin D	Arg Cys Ser
TyrMe-Asp-OH	Neodulin
Hydramethylnon	Ser-Nap-OH
Sulfacetamide	Asp Glu Asn
Bensulfuron-methyl	Carbaryl
Deoxyguanosine diphosphate (dGDP)	Docosanediol-1,14-disulfate
Glu-P-2	Oxmetidine
Tos-Arg-CH2Cl	Benzo[b]naphtho[2,1-d]thiophene
Arg Gly Gly	10-deacetylbaccatin III
luteolin 7-sulfate-3'-rutinoside	raclopride
Levan	Methitural
Watasenia luciferin	Herbacetin 4'-methyl ether
100-2	1,8-Diazacyclotetradecane-2,9-dione
Rehderianin I	4-Hydroxyaminoquinoline N-oxide
Hypromellose	5-(3,4-Dihydroxy-1,5-cyclohexadien-1-yl)-5-ethylbarbituric acid
4,4-Disubstituted cyclohexenone	C75
Digitalose	Fluorouracil
PS(P-18:0/22:1(11Z))	His-His-OH
(+/-)-trans-Acenaphthene-1,2-diol	Quinol glucuronide
Fusarenone X	2-Amino-4-oxo-6-(1,2,3-Trihydroxypropyl)-diquinoid-7,8-dihydroxypterin

11-Hydroxyiridodial glucoside pentaacetate	3,5,7,3',5'-Pentahydroxy-6,4'-dimethoxyflavone
Edulane	CAY10506
Fenvalerate	2S-aminoheptanoic acid
dCMP	Apocynin A
Gly-Asp-OH	Lys-Ser-OH
LY293111	Sepiapterin
1Î±,25-dihydroxy-2Î²-(2-hydroxyethoxy)vitamin D3 / 1Î±,25-dihydroxy-2Î²-(2-hydroxyethoxy)cholecalciferol	Eremophilenolide
Cys Met	Herbacetin 7-methyl ether 3-(2"-(E)-feruloylglucoside)
D-Glucuronic acid	Licoflavone A
Met-His-OH	6-methyltetrahydropterin
N-stearoyl histidine	Idebenone Metabolite (Benzenehexanoic acid, 2,5-dihydroxy-3,4-dimethoxy-6-methyl-)
His-TyrMe-OH	PMEG
PtdIns-(1,2-dioctanoyl)	3-(2,3-Dihydroxyphenyl)propanoate
2-Methyl-5-isopropylhexa-2Z,5-dienal	Leucodelphinidin 3-O-(beta-D-glucopyranosyl-(1->4)-alpha-L-rhamnopyranoside)
PG(15:1(9Z)/0:0)	Zwittermicin A
5-O-Methylbiochanin A	Acetylaminodantrolene
Rigin	Cefuroxime
4'-Hydroxyflurbiprofen	Chaksine
Furosemide	Ethosuximide M3
(S)-Dihydroorotate	Loganic Acid
13,14-dihydro-PGE1	Heterodendrin
1-Fluoro-25-hydroxy-16-ene-23-yne-26,27-hexadeuterovitamin-D3	(-)-Nutlin-3
5-Heptynoic acid, 7-hydroxy-; 7-Hydroxy-5-heptynoic acid	2H-Dibenz[b,f]azepin-2-one
Chorismate	Piceid
1-Naphthoic acid glucuronide	2-Phenethylsulfanyl-5,6,7,8-tetrahydro-benzo[4,5]thieno[2,3-d]pyrimidin-4-ylamine
2-Octenedioic acid	Carajuflavone
3,4-Dihydroxy-3,4-dihydro-9-fluorenone	2',3',5'-triacetyl-5-Azacytidine
4-Sulfobenzaldehyde	Bacteriochlorophyllide b
Apetalolide	4-Methylumbelliferyl Î²-D-glucuronide
Gly Lys Phe	6-Methoxytaxifolin
Triflusulfuron-methyl	N4-Acetylcytidine
2-Aminomethylpyrimidine	N-D-Ribosylpurine
3-Chloro-8Î²-Hydroxycarapin, 3,8-Hemiacetal	Chlorocresol
Isoxathion	Glutaconic acid
Rhizocticin B	Afrormosin

Bruceantinol	4,7,10,13,16,19-Docosahexaynoic acid
quinoline alkaloid	Trolamine
Tazobactam	7,8-Didemethyl-8-hydroxy-5-deazariboflavin
3',4'-Dimethoxyflavone	7-Methyl-2-hydroxy-6-oxoocta-2,4-dienoate
9,10-dihydroxy-hexadecanoic acid	Leu Pro Pro
Cobalt-dihydro-precorrin 6	Aspidinol
6-Hydroxy-7-methyl-3',4',5'-trimethoxyaurone 4-O-rhamnoside	Irisxanthone
Phe Asn Val	Tephcalostan C
Cys Glu Glu	Flavine mononucleotide (FMN)
Herbacetin 8-(2'',3'',4''-triacetylxyloside)	Hydrazinopthalazinone
His Pro His	Leonurine
Ile Glu	Proacacipetalin
Picein	3-Fumarylpyruvate
Sudan I	(+)-Gallocatechin
UDP-2-acetamido-4-amino-2,4,6-trideoxyglucose	Arg Gly Gly
Ethosuximide M3 glucuronide	Piperacillin
Leucodelphinidin 3-O-(beta-D-glucopyranosyl-(1->4)-alpha-L-rhamnopyranoside)	2-(Hydroxymethyl)-3-(acetamidomethylene)succinate
JWH 073 N-butanoic acid metabolite	N-octanoyl-L-Homoserine lactone
Tebuthiuron	Sulfamethizole
5-(3-Buten-1-ynyl)-2,2'-bithiophene	19-hydroxy-nonadecanoic acid
CBS 113A	2,2-Dimethyl-3-(4-methoxyphenyl)-4-propyl-2H-1-benzopyran-7-ol acetate
4-(4-Deoxy-Î±-D-gluc-4-enuronosyl)-D-galacturonate	7-Hydroxyethyltheophylline
Convalloside	Kaempferol 3-apioside-7-rhamnosyl-(1->6)-(2''-(E)-caffeoylglactoside)
Doxefazepam	His Ser Trp
Shiromodiol diacetate	2-Methyl-5-isopropylhexa-2Z,5-dienal
Melithiazole E	Ethyl phenothiazine-2-carbamate
Telomestatin	Silandrin
14,15-HxA3-D(11S)	2-Hydroxyzotepine
AL-321	3D-(3,5/4)-Trihydroxycyclohexane-1,2-dione
5-Hydroxypyrazinamide	Gly Gly Phe
Panaxytriol	Asp Cys Asp
5-L-Glutamyl-taurine	Carindacillin
Miglitol	8-Cinnamoyl-3,4-dihydro-5,7-dihydroxy-4-phenylcoumarin
Gentisein	Pro Asp
SC-19220	10-Hydroxycamtothecin
Thien-2-ylacetate	2-glyceryl-6-keto-PGF1Î±
Met-Met-OH	Asp-HoPhe-OH

Tyr Asp	Gossypetin 3-sophoroside-8-glucoside
Gemcitabine	Î²-vinyl acrylic acid
5,7,2',3',4'-Pentahydroxy-3,6-dimethoxyflavone 7-glucoside	MID42072:(25S)-26,26,26-trifluoro-1Î±,25-dihydroxyvitamin D3 / (25S)-26,26,26-trifluoro-1Î±,25-dihydro
Fluoranthene	6-Prenyleriodictyol
Phytolaccoside B	8-Caffeoyl-3,4-dihydro-5,7-dihydroxy-4-phenylcoumarin
MS-275	Diazepam
O-Desmethylangolensin	Broussoflavonol B
Desmethyl methotrimeprazine 5-sulfoxide	DG(20:0/21:0/0:0)[iso2]
Drimenin	fosphenytoin
Mannopine	Pentoxazone
Epigallocatchin 3-O-(4-hydroxybenzoate)	Pretetramid
Labetalol	5,12-Dihydroxanthommatin
Trilobatin	Icilin
(S)-2-Aceto-2-hydroxybutanoic acid	Mefenamic acid Metabolite (b-D-Glucopyranuronic acid, 1-[2-[(3-carboxy-2-methylphenyl)amino]benzoate
Ala Val Tyr	Metaproterenol 3-O-sulfate
2-Protocatchoylphloroglucinolcarboxylate	Met-Ser-OH
Flaccidine	Thonningianin B
Gln Trp Trp	17Î±,20Î²-Hydroxyprogesterone sulfate
p-Salicylic acid	Avenanthramide-C methyl ester
202-791	Ro 48-8071
apigenin 7-galactoside	Asn Gly
Tricin 7-rutinoside-4'-glucoside	S-ethyl N-[4-(trifluoromethyl)phenyl] Isothiourea
Bergenin	Sulfometuron
Dextrorphan sulfate	Cys Cys Gln
Phenazepam	Limonoate
Simmondsin	3-hydroxy-tetradecanedioic acid
Uroporphyrin I	Lys Pro Glu
Ala Trp Gly	4-Hydroxycinnamic acid
Disulfiram	Val Asp Met
N-Methyl-(R,S)-tetrahydrobenzylisoquinoline	Suberic acid
Lyngbyatoxin	3,8-dimethoxy-5,7-dihydroxy-3',4'-methylenedioxyflavone
Thr Ser Cys	Glu Asp Asp
4-Benzyloxy-2'-hydroxy-3',4',5',6'-tetramethoxychalcone	N4-Acetylsulfadimidine
Ser Pro Arg	3-sulfopropyltrimethylsilane
Xipamide O-glucuronide	4-Fluorocyclohexadiene-cis,cis-1,2-diol

Acetyl-maltose	Adunctin E
Leu-Asp-OH	Val Asp Pro
Phosphodimethylethanolamine	Xanthopterin-B2
Chrysosplenin	3-keto-n-caproic acid
Prochlorperazine sulfone	Adenosine-3'-monophosphate
Triangularine	Pro Cys Pro
4-Nitroquinoline-1-oxide	4'-O-Methylcarthamidin 7-(2-p-coumaroylglucoside)
Asp-Asp-OH	Daunorubicinol
Cucurbitacin P	Tepanone
Digitoxigenin monodigitoxoside	Ala Trp Cys
oleanolic acid 3-O-beta-D-glucosiduronic acid	Arbutin
3-O-acetylecdysone 2-phosphate	Thiodiacetic acid sulfoxide
Gly-Abu-OH	Athamantin
1-(2-Carboxyphenylamino)-1'-deoxy-D-ribulose 5'-phosphate	Azafenidin
HDBA	10-Hydroxy-3,7-dimethyl-2E,6E-decadienoic acid
Sulfamerazine	Euchrenone b3
Gossypetin 3,3'-dimethyl ether	N-Feruloylglycine
(+)-trans-allethrin	(4S)-7-Hydroxy-4-isopropenyl-7-methyl-2-oxo-oxepanone
3,4-Dihydroxymandelaldehyde	Naphthofluorescein
Phenyl acetate	Northienamycin
luteolin 7,3'-dimethyl ether 4'-glucoside	Quercetin 3-(3'',6''-di-p-coumarylglucoside)
Leu Ile	XAV939
Isorhamnetin 3-glucosyl-(1->6)-galactoside-7-glucoside	7-Hydroxy-3',4'-dimethoxyflavone
Met His	Asn Cys Thr
Cromolyn	Hexandraside E
Gln Tyr Cys	Thiamethoxam
2-[3-Carboxy-3-(methylammonio)propyl]-L-histidine	8-Hydroxymianserin
3-Galactosyllactose	Kaempferol 3-(6''-sinapylglucosyl)-(1->2)-galactoside
Haplogenin	Wedelolactone
tBu-Honaucin A	Asn Asp Cys
Diguanosine diphosphate	12-hydroxyjasmonic acid
Asn Ile Asn	1-Phenyl-3-(phenylsulfonyl)-2-propen-1-one
His Arg Ile	His Asp
Gly Met Met	Goitrin
Mefenamic acid Metabolite (b-D-Glucopyranuronic acid, 1-[2-[[3-(hydroxymethyl)-2-methylphenyl]amino]	MID41991:(5Z)-1Î±,25-dihydroxy-3-deoxy-3-thiavitamin D3 3-oxide / (5Z)-1Î±,25-dihydroxy-3-deoxy-3-thia

Met-Ala-OH	Probenecid
Diacetoxyscirpenol	Mefloquine
Vanillin	Phytolaccoside B
Sebacic acid	APF
2-Oxosuberate	Cerarvensin 2''-O-rhamnoside
3-(Pyrazol-1-yl)-L-alanine	Dihydrotricetin 7,3'-dimethyl ether
Deoxyribonolactone	Neburon
Triflumuron	Ranunculin
Landomycin D	Triethylene glycol diglycidyl ether
Pro Glu Trp	6-Hydroxykynurenate
PE(20:3(8Z,11Z,14Z)/20:0)	N-(7-Mercaptoheptanoyl)threonine 3-O-phosphate
7-Methylpyrido[3,4-c]psoralen	Salidroside
Asperuloside	3-Methylcholanthrene
quercetin 3-(6''-malonylglucoside)	Barbituric acid, 5-ethyl-5-(2-hydroxyethyl)-
Docosahexaenoyl Serotonin	Flaviolin
Pyrocatchol glucuronide	His His Arg
Demethylcitalopram	3-o-Ethyl-L-ascorbic acid
Tebufenpyrad	4-Chloro-4'-biphenylol
quercetagetin 4'-methyl ether 7-(6-(E)-caffeylglucoside)	Quizalofop-ethyl
Piretanide	8-Oxocoformycin
Triticonazole	Inosine 5'-monophosphate (IMP)
1-O-Feruloyl-Î²-D-glucose	TyrMe-Phe-OH
Rimonabant	Alamarine
Gly Phe	N-Desmethylperazine
Lys-Lys-OH	Acacetin 7-(2G-rhamnosyl)-rutinoside
NSC 210902	Leu-Ala-OH
Phellatin	Suxibuzone
Eicosapentaenoyl Serotonin	3,4-Dehydrochlorambucil
Phe Pro Lys	Naringenin 7-(4,6-digalloylglucoside)
6-Hydroxy-5-methoxyindole glucuronide	Quercetin 3-(2''-acetylgalactoside)
Calcein	6-oxo-Nonan-1-ol
(Â±)8-gingerol	Gallocatechin-4beta-ol
1,2-Benzisoxazole, 6-fluoro-3-(4-piperidinyl)-	cis-Zeatin riboside monophosphate
4,4'-Biphenyldithiol	Clomeprop
5-Phosphonooxy-L-lysine	farnesyl triphosphate
Oxazepam	Phe4Cl-Met-OH
Angustone C	Quercetin 5,7,3',4'-tetramethyl ether 3-rutinoside
Oligomycin D	2-Deoxystreptidine
6alpha,9-Difluoro-11beta-hydroxypregn-4-ene-3,20-dione	Paroxetine

9-oxo-2E-decenoic acid	Kikkanol C
Tyr Lys Ser	Trichilin A
3-Hydroxydodecanedioic acid	Viomycin
D-Ribose	N1-Amidinostreptamine 6-phosphate
10E,12E-tetradecadiene-4,6-diynoic acid	Cetirizine
Broussonol E	5,6,5'-Trihydroxy-3,7,2',4'-tetramethoxyflavone
Fludiazepam	Glycerophospho-N-Oleoyl Ethanolamine
Fusicoccin H	Musk xylene
Tyr Tyr Asn	(Â±)12,13-DiHOME
Ala Ala Met	Isorhamnetin 3-rhamnoside-7-sophoroside
Phosphoribosylglycinamide	Sulfaquinoxaline
(-)-Amurensisin	5,4'-Dihydroxy-6-C-prenylflavanone 4'-xylosyl-(1->2)-rhamnoside
CE-108	Ammoresinol
N-methylundec-10-enamide	Diospyrin
Evasterioside A	Methadone
Isoembigenin	Pirimiphos-ethyl
DHAP(8:0)	Sulfacetamide
Glucosylgalactosyl hydroxylysine	edetate
Ethosuximide M3	Normetanephrine sulfate
Asn-Met-OH	D-Camphorsulfonate
Met Pro	Ecgonine
(-)-Pinoresinol glucoside	Pyridoxamine-5'-Phosphate
Ile Ile Asp	Diclocymet
Isobutylglycine	Rhein glucuronide
Phenylmercuric Acetate	Cys Asp Arg
Piperidolate	5-(2'-Carboxyethyl)-4,6-Dihydroxypicolinate
Thr Tyr Trp	PD 169316
Triamcinolone acetonide glucuronide	MG(0:0/22:2(13Z,16Z)/0:0)
10-Hydroxy-3,7-dimethyl-2E,6E-decadienoic acid	5,7-Dihydroxychromone
MID42317:1Î±,25-dihydroxy-24a,24b-dihomo-23-oxa-20-epivitamin D3 / 1Î±,25-dihydroxy-24a,24b-dihomo-23-	Astringin
Pandamine	CAY10429
PI(17:2(9Z,12Z)/14:1(9Z))	Indanofan
Pro Gln Pro	R207910
CAY10638	Didymocalyxin B
Acalyphin	Quercetin 3-(2''-galloylgalactoside)
Deoxyloganin tetraacetate	Ala Met
3-Amino-4,7-dihydroxy-8-methylcoumarin	Enilconazole
5-Acetylamino-6-formylamino-3-methyluracil	N6-(Î´2-Isopentenyl)-adenosine 5'-monophosphate
Desmethylzopiclone	Thioquinox

Estra-1,3,5(10)-triene-3,17beta-diol 3-phosphate	Acevaltrate
Myricetin 3-(6"-galloylglucoside)	Matteuorienate B
4,5-Dihydroxysulfadiazine	2"-O-alpha-L-Rhamnosyl-6-C-fucosyl-3'-methoxyluteoiin
Cucurbitacin O	4-Methylumbelliferyl heptanoate
Lys Arg Leu	Lithospermic acid
MG(0:0/22:2(13Z,16Z)/0:0)	Met His Cys
10,11-Dihydro-dihydroxy-carbamazepine	Pteroyltriglutamic acid
Lys His Val	Desmethylnaproxen-6-O-sulfate
2-Phenylaminoadenosine	estrone 3-sulfate
E,E-Dienestrol-2,3-oxide	Pyraclonil
3-propylmalic acid	Allopurinol-1-ribonucleoside
Hosloppin	Pro Arg Ile
Oxidized Renilla luciferin	9-oxo-2E-decenoic acid
catchin 4'-O-beta-D-glucopyranoside	Kaempferol 3-rhamnosyl-(1->2)-galactoside-7-rhamnoside
Erosnin	makisterone B
Chlorbufam	NS 1608
Nifurthiazole	(-)-11-hydroxy-9,10-dihydrojasmonic acid 11-beta-D-glucoside
PI(21:0/0:0)	Silymarin
Fucosyllactose	Diethyl Oxalpropionate
TyrMe-Nap-OH	Millettosin
5-undecenoic acid	17-Octadecene-5,7,15-triynoic acid, 18-bromo-, (E)-
Tyr Glu Trp	2'-Hydroxyflavone
3-Hydroxyclonazepam	Cucurbitacin O
Copalyl diphosphate	Zalcitabine
Dihydropteroic acid	7-Methoxyflavone
Asp Arg Asp	Didesmethylloperamide
Laurencione diacetate	(Â±)7-epi Jasmonic Acid
Paroxetine	4,2',3',4'-Tetrahydroxychalcone 4'-O-(2"-O-p-coumaroyl-6"-O-acetyl)glucoside
Leucadenone D	Chlorprothixene
Thr Cys Ser	Fortimicin FU-10
Astragaloside III	Ala-Phe4Cl-OH
Niflumic Acid	Epiafzelechin 3-O-gallate
quercetin 4'-isobutyrate	Dehydrophytosphingosine
Semiglabrin	1,3-Glyceryl dinitrate glucuronide
11E-octadecen-9-ynoic acid	Ala-Ser-OH
aldicarb	Faratroside
Glu Lys Glu	Isosorbide 5-mononitrate glucuronide
Pantoic acid	mucic acid

quercetin 3-galactoside-7-xyloside	Lys Thr Thr
2-Hydroxy-6-oxo-6-phenylhexa-2,4-dienoate	Flumazenil acid
4-(2-Chlorophenyl)-5-methoxycabonyl-3-ethoxycarbonyl-6-methylpicolinic acid	1,2,4-Triazole-3-carboxamide
PI(P-20:0/22:4(7Z,10Z,13Z,16Z))	3-Hydroxynitrazepam
Cys Tyr Cys	4-Oxoglutaramate
Isoduartin Methyl Ether	Miraxanthin-III
Zaprinast	6-Hydroxyluteolin 6,4'-dimethyl ether 7-glucoside
2-hydroxy pelargonic acid	Granisetron metabolite 4
Diphenoxylic acid(DPA)	3S-hydroxy-decanoic acid
Olmesartan Medoxomil	6-Deoxyjacareubin
Robustaol A	b-D-Glucopyranosiduronic acid, 3-(6-methoxy-2-naphthalenyl)-1-methylpropyl
Stoloniferone M	Gly-Gly-OH
luteolin 5-(6"-malonylglucoside)	Isorhynchospermin
Arg Ala His	Methicillin
Thalassemine	Pranlukast
Arg Pro Glu	Asp Gly Thr
PI(O-20:0/17:0)	Quercetin 3-methyl ether 5-glucoside-3'-sulfate
Septentriodine	1-O,6-O-Digalloyl-beta-D-glucose
Clitidine 5'-phosphate	2,4-Diamino-6,7-dimethoxyquinazoline
Clofop	3-Hydroxylidocaine glucuronide
Digitoxigenin bisdigitoxoside	4-Hydroxyclobazam
Digoxigenin	Asn Cys Asp
Imidazoleacetic acid ribotide	N-Succinyl-L-diaminopimelic acid
N-Ac-Tyr-Val-Ala-Asp-CMK	Indole-3-acetaldehyde oxime
Linoside B	Ovalitenin C
Chlorpropham	Perindopril
dTDP-D-oliose	4-Nitroquinoline-1-oxide
Nicarbazin	Mycothiol
Mecoprop-P	1,3,4,5-Tetracaffeoylquinic acid
(-)-Columbianetin	CAY10597
Encecalin	Leu Ala Glu
Lophirone D	4-Hydroxy-L-threonine
Ethylenethiourea	Kaempferol 7-methyl ether 3-[3-hydroxy-3-methylglutaryl-(1->6)]-[apiosyl-(1->2)-galactoside]
Tribenuron methyl	Ritipenem acoxil
2,6-Dioxo-6-phenylhexanoate	3-Chloro-8Î²-Hydroxycarapin, 3,8-Hemiacetal
iodovulone I	6alpha-Fluoro-17-hydroxypregn-4-ene-3,20-dione acetate
Oxydeprofos	Benzoximate
Diethyldithiocarbamate (DDC)	Diethylstilbestrol monosulfate

Isoscutellarein 7-(6'''-acetylallosyl-(1->2)-6''-acetylglucoside)	desethyletomidate
2,3-dinor-PGE1	Pravadoline
7-N,N-Dimethylamino-1,2,3,4,5-pentathiocyclooctane	N-Acetylcarbocysteine
Arg Phe Phe	3-Oxo-3-ureidopropanoate
Methylarbutin	Caloxanthin sulfate
Cys Gly Asn	Limocitrol 3-[alpha-L-arabinopyranosyl-(1->3)[galactosyl-(1->6)]-galactoside]
TG(18:1(9Z)/20:4(5Z,8Z,11Z,14Z)/22:5(7Z,10Z,13Z,16Z,19Z))[iso6]	MID73229:(1aalpha,2beta,3alpha,11calpha)-1a,2,3,11c-Tetrahydro-6,11-dimethylbenzo[6,7]phenanthro[3,4
Enalapril	Tyr-Thr-OH
Asn-Ala-OH	(S)-N-[3-(3,4-Methylenedioxyphenyl)-2-(mercaptomethyl)-1-oxoprolyl]-(S)-alanine
Methylophiopogonone A	Clofazimine
Phe Leu Asp	Propicillan
Bacteriochlorophyllide b	(Â±)-Mucronulatol
Tyr Phe Tyr	2-Keto-6-acetamidocaproate
5-(3-Hydroxy-4-acetoxybut-1-ynyl)-2,2'-bithiophene	Anastatin B
7-Deoxygardoside metyl ester tetraacetate	Apodine
Losartan Metabolite (1H-Imidazole-2-propanol, 4-chloro-5-(hydroxymethyl)-a-methyl-1-[[2'-(1H-tetrazo	Glutarylglycine
Parthenosin	Perindopril lactam
Ser Met Trp	Quercetin 5,7,3',4'-tetramethyl ether 3-galactoside
URB447	4Z-decenoic acid
Deracoxib	Nuarimol
Metronidazole	2-methyl-tridecanedioic acid
5'-Dehydroadenosine	Luteoskyrin
Chebulinic acid	9-bromo-decanoic acid
10-Hydroxymorroniside	CI Pigment Red 3
2,3',4,6-Tetrahydroxybenzophenone	Methyltriazolopthalazine
CAY10429	Pteryxin
4,14-dihydroxy-octadecanoic acid	Ser-Phe4Cl-OH
4'-Methoxychalcone	Tyrphostin B44 (-)
His-Met-OH	2-Hydroxy-4,5,6-trimethoxydihydrochalcone
Anthragallol	3-butyrl propionic acid
N-(2-fluro-ethyl)-eicosanoyl amine	Cyclohexylsulfamate
Stercurensin	Ketamine metabolite (Cyclohexanone, 2-amino-2-(2-chlorophenyl)-6-hydroxy-)

NNAL-N-glucuronide	trans-2-[(Dimethylamino)methylimino]-5-[2-(5-nitro-2- furyl)-vinyl]-1,3,4-oxadiazole
PI(P-20:0/22:6(4Z,7Z,10Z,13Z,16Z,19Z))	Corpaine
Hydrocortisone caproate	Gly Ala Cys
L791456	3-tert-Butyl-5-methylcatechol
Nivalenol in acetonitrile	Geraniin
Halosulfuron-methyl	L-161,982
N,N'-Diethylthiourea	Sulfisoxazole
S-Prenyl-L-cysteine	Val Ile Asp
7-oxo-11E,13-Tetradecadienoic acid	2-Fluoroaniline
6-Formylindolo [3,2-B] carbazole	6-Desmethoxy hormothamnione diacetate
Cys Ile	Licodione
3-(2-chloro-10H-phenothiazin-10-yl)propan-1-amine	Luteolin 7-O-(2-apiofuranosyl-4-glucopyranosyl-6-malonyl)glucopyranoside
Demeton-S-methyl	Phe4Cl-Ser-OH
Fluticasone 17-carboxylic acid	Pro Met Asn
4-Hydroxyriluzole glucuronide	9alpha-Fluoro-11beta,16alpha,17alpha,21-tetrahydroxypregn-4-ene-3,20-dione
Ceftizoxime	Phenylenediamine
Acacetin 7-(4'''-acetylrutinoside)	Streptamine phosphate
L-Cysteinesulfonic acid	Thiadiazin
MID42524:(24aE)-1Î±,25-dihydroxy-24a,24b-didehydro-24a,24b-dihomovitamin D3 /(24aE)-1Î±,25-dihydroxy-2	Tuliposide B
Glu Glu Lys	Isoorientin 2''-p-hydroxybenzoate
MID42072:(25S)-26,26,26-trifluoro-1Î±,25-dihydroxyvitamin D3 / (25S)-26,26,26-trifluoro-1Î±,25-dihydro	1-Hydroxyhexane-1,2,6-tricarboxylate
Phe-Nap-OH	2-(2-Chloro-phenyl)-5-(5-methylthiophen-2-yl)-[1,3,4]oxadiazole
5a,11a-Dehydrooxytetracycline	Silyhermin
Cys Tyr Asn	5'-Phosphoribosyl-N-formylglycinamide (FGAR)
Cyclohexylsulfamate	Phytosphingosine
Griseofulvic Acid	Premithramycin A2'
phytyl diphosphate	Apigenin 4'-glucoside
Syringic acid	Isomollupentin 7-O-glucoside-2''-O-arabinoside
Tetracenomycin B1	Jaceidin 7-neohesperidoside
Ala Ile His	1-(3-Carboxypropyl)-3,7-dimethylxanthine
Quinalphos	Annolobine
Spiredine	Hypusine
Okanin 4-methyl ether 4'-O-(2''-O-caffeoyl-6''-O-acetylglucoside)	AMPA
11-O-Demethyl-7-methoxypradinone II	Ile-Leu-OH

B-(4-Fluorobenzoyl)propionic acid	Isopentenyladenosine-5'-diphosphate
Brianthein X	N-Nitrosofolic acid
farnesyl triphosphate	Pyriftalid
tetrahydro-L-Biopterin	Sucrose-6-phosphate
2,3-DCPE	Tegafur
Coronatine	Asp Tyr Cys
Gln Phe Gly	Pubescenol
Leu Ala Glu	Chlorohyssopifolin A
Fenofibric acid	Ketoconazole Metabolite (2,3-Piperazinedione, 1-[4-[[2-(2,4-dichlorophenyl)-2-(1H-imidazol-1-ylmethy
Penicillamine cysteine disulfide	Rhynchosin
Colnelenic acid	Spirodiclofen
Dolichyl b-D-glucosyl phosphate	Thiotepa
Lamioside	AM630
Patuletin 7-O-sulfate	CAY10505
2,4,6-trimethyl-3,5-dinitrobenzonitrile	Gln Ala Lys
Asp Thr Tyr	Hexythiazox
Hoslundal	L-Canaline
Mometasone Metabolite (Pregna-1,4-diene-3,20-dione, 9,21-dichloro-6,11,17-trihydroxy-16-methyl-, (6a	Atheroline
3-(2-Carboxyethenyl)-cis,cis-muconate	Diflufenican
8-Azaadenosine	(1E)-4-Oxobut-1-ene-1,2,4-tricarboxylate
AL 8810	11,12-Dihydroxybenzo[a]pyrene
Isoflavone 7-O-beta-D-glucoside	Dibenzo[a,e]fluoranthene
Ala Pro Gln	Nap-Nap-OH
Gelsemicine	2,8-Dihydroxyadenine
Scopolin	B-Norcholest-4-en-3-one
Ala Lys Thr	Xylobiose
Monotropein	Coformycin
Gnidicin	Dodecyl glucoside
Morphine 6-sulfate	6-Desmethoxy hormothamnione triacetate
Endecaphyllin X	Acetyl-maltose
Pristinamycin IB	Asn Gly Ala
Fluconazole glucuronide	Norbixin
Letrozole	PI-103
MID66857:alpha-Galactosyl-(1-6)-alpha-galactosyl-(1-6)-alpha-galactosyl-(1-6)-alpha-galactosyl-(1-6)	(E)-2-(2-Furyl)-3-(5-nitro-2-furyl)acrylamide
Metaproterenol	4,4-Difluoro-17beta-hydroxyandrost-5-en-3-one propionate
CI Acid Orange 3	Asn Ser Asp

Fluoren-9-one	1,3-Dipropyl-8-cyclopentylxanthine [DPCPX]
Isocaviunin 7-O-glucoside	Isouvaretin
1,7-Dimethyluric acid	Neotenone
AK-toxin I	Tyr-Phe4Cl-OH
Epoxy Fluor 7	Halofenozide
18-Hydroxycortisol	P1,P2-Bis(5'-adenosyl) triphosphate
4-PIOL	5,7,4',5'-Tetrahydroxy-3,6,2'-trimethoxyflavone
Cys Cys Asp	His Asp Lys
Cefixime	N2,N2-Dimethylguanosine
furazolidone	Zopiclone N-oxide
Glu Glu Arg	(+)-Blebbistatin
PS(20:1(11Z)/18:3(9Z,12Z,15Z))	Bunazosin
8S-hydroxy-9Z,12Z-octadecadienoic acid	DG(20:1(11Z)/24:0/0:0)
Arg His Glu	Solanidane skeleton
Cefpodoxime	Auranofin
D-Glucosamine 6-phosphate	Sclerotiorin
Gitoxin	Boscalid
quinol glucuronide	lipoamide
Trp-P-2	SC-1271
Enantiomultijugin	2H-1-Benzopyran-2-one, 6-(1,2-dihydroxyethyl)-7-hydroxy-8-methoxy-glucuronide
Stictic Acid	2-Hydroxy-3,4-dimethoxybenzoic Acid
5-Phosphoribosylamine	Met Glu Met
Deacetyl-N-monodemethyldiltiazem	5'-Methoxyhydnocarpin-D
Glutathionylaminopropylcadaverine	Antimycin A1
N-Benzylphthalimide	Mebendazole metabolite (Carbamic acid, [5-(hydroxyphenylmethyl)-1H-benzimidazol-2-yl]-, methyl ester
Asp-Phe4Cl-OH	7-[2-Trifluoromethyl-4-(2-hydroxyphenyl)-1,3-dioxan-cis-5-yl]-hept-5z-enoic Acid
AVE-1625	Desmethylnizatidine
(6S)-6-Hydroxy-1,4,5,6-tetrahydronicotinamide-adenine dinucleotide	Erythrinin A
Beraprost	Isoorientin 7,3',4'-trimethyl ether
FKGK 11	N,N-Dimethylamiloride
Sulfoxotolrestat	PAPA NONOate
Diflunisal	Ketospirilloxanthin
2- (2,6-Dimethoxyphenoxyethyl)Aminomethyl-1,4-Benzodioxane (WB 4101)	N4-Acetylsulfadoxine
PA(21:4(6Z,9Z,12Z,15Z)/0:0)	Quercetin 7,3',4'-trimethyl ether
Streptidine	7-oxo-11E-Tetradecenoic acid
2-octenal	CAY10606

3-O-(6-O-alpha-D-Xylosylphospho-alpha-D-mannopyranosyl)-alpha-D-mannopyranose	Gln Tyr Lys
S-(2,2-Dichloro-1-hydroxy)ethyl glutathione	Phe4Cl-Tyr-OH
Azaleatin	Bromo-3-hydroxy-4- (succin-2-yl)-caryolane Î³-lactone
PG(17:0/0:0)	cis-4-Carboxymethylenebut-2-en-4-olide
Apraclonidine	Cys Gln Thr
2-Methacryloyloxyethyl phenyl phosphate	Purpuritenin A
Homolanthionine	Coriamyrtin
p-Hydroxymethylphenidate	Kurziflavolactone A
Daphnoline	Phe Gln Cys
4-Isopropylbenzoic acid	S-(3-Methylbutanoyl)-dihydrolipoamide-E
Met Asn Tyr	Stemonal
XE991	N-Acetylmuramic acid
2',3',5'-triacetyl-5-Azacytidine	Trifluridine
Spironolactone	Arg Trp Gln
5,3',4'-Trihydroxy-3-methoxy-6,7-methylenedioxyflavone 4'-glucuronide	Moxalactam
(3E)-4-(2-Carboxyphenyl)-2-oxobut-3-enoate	Chlorpromazine
Dihydrorhodamine 123	Fludiazepam
AM694	Liriodenine
Ethyl Tricosanoate	Euchrenone a15
3-quinolinecarboxylic acid, 7-amino-1-ethyl-6-fluoro-1,4-dihydro-4-oxo-	Abu-Phe4Cl-OH
Istamycin B0	Embigenin 2''-(2'''-acetylrhamnoside)
2-(1,3-Benzodioxol-5-yl)-3,5,6,8-tetramethoxy-7-[(3-methyl-2-butenyl)oxy]-4H-1-benzopyran-4-one	Cinoxacin
2,3-Dihydroisogedunin	Ile Leu
5'-Phosphoguanylyl(3'->5')guanosine	Lasonolide A
Pro Pro Trp	Longifolonine
2-Hydroxyhepta-2,4-dienedioate	Leu Gln Asp
Asn His Ser	Oxycarboxin
Eupacunolin	Î±-Cyano-3-Hydroxycinnamic Acid
Viomycin	N4-Phosphoagmatine
2-Phenylethanol glucuronide	Scarlet Red
2,6-Dinitrotoluene	8-Hydroxyalanylclavam
Cefaclor	Cassiaoccidentalin A
Luteoskyrin	Digitoxigenin bisdigitoxoside
Adynerin	Mequitazine
Lucanthone	7-Desmethylpapaverine sulfate
Slaframine	Anhydrotetracycline
dTDP-4-oxo-5-C-methyl-L-rhamnose	Isoscoparin 2''-(6-(E)-p-coumaroylglucoside)

His His	3-Hydroxydodecanedioic acid
Lobelanine	Estradiol disulfate
3-Methyluric acid	Ticlopidine
PG(22:6(4Z,7Z,10Z,13Z,16Z,19Z)/0:0)	Valerosidatum
3-methyl-dodecanedioic acid	Thenylchlor
Epimedoside E	3-(3-Methylbutyl)tricetin 5-neohesperidoside
Isobarbaloin	Gly-Ser-OH
Norathyriol	Tributyrin
Ser Asp Gln	Flavin adenine dinucleotide (FAD)
2-Aminoadenosine	N-(p-Nitrobenzyl)phthalimide
Phe-Met-OH	Formononetin 7-O-rutinoside
p-cresol	Rabeprazole
C20-OHSulfatide	RG-108
Neotenone	5,7,3'-Trihydroxy-6,4',5'-trimethoxyflavanone
N-hexanoyl-L-Homoserine lactone	Î²-D-Glucopyranosiduronic acid, 2-(1H-indol-4-yloxy)-1-[[(1-methylethyl)amino]methyl]ethyl
Betalamic acid	N2,N5-Dibenzoyl-L-ornithine
chlorovulone III	Parthenosin
N4-Phosphoagmatine	7a-Hydroxy-O-carbamoyl-deacetylcephalosporin C
5-(2'-Formylethyl)-4,6-dihydroxypicolinate	Dihydrojasmonic Acid
Bis(2-chloro-1-methylethyl)ether	Dofetilide
Lucernol	2-Chloro-5-methyl-cis-dienelactone
MID73251:6-[2,3-Dihydroxy-1-(hydroxymethyl)propyl]-1,2-dihydro-7-hydroxy-9-methoxy-cyclopenta[c][1]b	Asn Phe His
Rhein glucuronide	8-Oxo-dGMP
Arbutin	Diallyl disulfide
Benzo[b]naphtho[2,1-d]thiophene	Glycyl-H-1152
Mascaroside	N,N'-Diacetylchitobiosyldiphosphodolichol
MDL 73492 sulfate	TG(16:1(9Z)/16:1(9Z)/17:2(9Z,12Z))[iso3]
1,2-Dihydroxy-3,4-epoxy-1,2,3,4-tetrahydronaphthalene	Thiamylal
MID41992:(5Z)-(3S)-1Î±,25-dihydroxy-3-deoxy-3-thiavitamin D3 3-oxide / (5Z)-(3S)-1Î±,25-dihydroxy-3-de	AL-294
14S-hydroxy-hexadecanoic acid	Bucladesine
Tinidazole	Cys Asn Met
Ser Asn Gly	HPF
Thr Asn Ser	Luteolin 7-(6'''-acetylsophoroside)
Gossypetin 7-methyl ether 8-acetate	Metoxuron
Ser Ser Ala	19-Hydroxytetrangulol
AM-toxin I	dCMP
Nitrendipine	Isochamaejasmin

N-stearoyl phenylalanine	TG(18:3(9Z,12Z,15Z)/18:3(9Z,12Z,15Z)/18:3(9Z,12Z,15Z))
SB 242084	TyrMe-Ala-OH
Aspidinol	Trinexapac-ethyl
b-D-Glucopyranuronic acid	Cacalol
Terminalin	(Â±)14-HDoHE
3-Piperidinemethanol, 4-(4-fluorophenyl)-, (3S,4R)-glucuronide	Alprostadil alfadex
Fluprostenol Lactone Diol	L-Adrenaline
quercetin 3-(2"-galoylrutinoside)	Met Cys Thr
Guazatine	SN-38
N2-Succinylglutamic acid	Thymusin 6-isobutyrate
24Z-ethylidene-cholest-5-en-3beta-ol 3-O-beta-D-glucopyranoside	Quercetin 3-(6"-malonylglucoside)-7-glucoside
Candesartan	Triamcinolone
Guibourtinidol-4alpha-ol	ThioFluor 623
3,5-Dinitro-4-hydroxyphenylpyruvate	9-Fluoro-17beta-hydroxy-6alpha,17-dimethylandrost-4-ene-3,11-dione
Ala Glu His	CCG-1423
3'-O-methylbatatasin III	PG(15:1(9Z)/0:0)
Scopoloside I	Pyrazinoic acid
Simplexin	Devazepide
UDP-4-amino-4,6-dideoxy-N-acetyl-beta-L-altrosamine	Glu Val Ala
Myricetin 3'-O-(6"-p-coumaroyl)glucoside	Kaempferol 7-xyloside
Met Pro Pro	Lophirone J
1-[2-Bromo-1-(4-chlorophenyl)ethenyl]-2-chlorobenzene	Valdecoxib
5-(hydroxymethyl)- 2-Furancarboxylic acid	1-(5'-Phosphoribosyl)-5-formamido-4-imidazolecarboxamide
MID42071:(25R)-26,26,26-trifluoro-1Î±,25-dihydroxyvitamin D3 / (25R)-26,26,26-trifluoro-1Î±,25-dihydro	Arg Arg Lys
Yellow AB	Atrolactic acid
Thr Lys Thr	Fusicoccin H
Caloxanthin sulfate	2,3,4-Trihydroxybenzylhydrazide
9,13-octadecadiynoic acid	3,5,7-Tris(acetyloxy)-2-[4-(acetyloxy)-3-hydroxyphenyl]-4H-1-benzopyran-4-one
Gly Pro Lys	Cephalexin
DIBOA-glucoside	Dioclein
Lasalocid A	LysoPE(0:0/24:6(6Z,9Z,12Z,15Z,18Z,21Z))
Transfluthrin	Methyl bisnorbiotinyl ketone
agavoside A	Phe4Cl-Ile-OH
Gly Asn Asp	SK&F 91581

Minabeolide-7	S-Pyruvylglutathione
m-Trifluoromethylhippuric acid	3,4-Dihydroxy-3,4-dihydro-9-fluorenone
Cassiaoccidentalin A	5Î²-Cholestane-3Î±,7Î±,12Î±,24,26-pentol
Diethylstilbestryl disulfate	Leukotriene E3
2',2'-Bisepigallocatchin Digallate	Prontosil
Clorazepate	Epigallocatechin 3-O-cinnamate
Dihydrorobinetin	Lys Gly His
Hydrocortisone cypionate	Met Asn Pro
Met-Ser-OH	Oxapyrazon
(S)-AL 8810	Perazine
Glu Thr Leu	5,7,4'-Trihydroxy-8,3'-dimethoxyflavanone
Inosine 2',3'-cyclic phosphate	Griseofulvin
(10S)-Juvenile hormone III acid diol	Hycanthone
7-Aminocephalosporanic acid	Luteolin 4'-sulfate
DHAP(10:0)	Patuletin 3-(6''-p-coumaroylglucoside)
Phosphamidon	Phe Lys His
Kaempferide 5-glucoside-7-glucuronide	Hexandraside C
(4S)-7-Hydroxy-4-isopropenyl-7-methyl-2-oxo-oxepanone	TG(18:0/18:2(9Z,12Z)/20:4(5Z,8Z,11Z,14Z))[iso6]
Alectrol	Val Phe
Filipin III	Catheduline E2
Thr-Phe-OH	Myricetin 3-acetylrhamnoside
MG(0:0/20:2(11Z,14Z)/0:0)	p-Chlorobenzhydrol
Thimerosal	Anhydrochlortetracycline
3-oxo-dodecanoic acid	Urdamycin B
Gluconapin	C-8 Ceramide
Ile Ala Val	Nalmefene
L787257	N-Methylanthraniloyl-CoA
Reduced flavine adenine dinucleotide (FADH2)	11-dehydro-TXB2-d4
GW 409544	2-glyceryl-PGE2
Lys Glu Glu	7-Methoxy-5,6:3',4'-bis(methylenedioxy)flavone
1,8-Diazacyclotetradecane-2,9-dione	Apuleisin
Methylmalonyl-CoA	Carfecillin
Tris(2-chloroethyl)phosphate	Epicatechin 3-O-(2-trans-cinnamoyl-beta-D-allopyranoside)
3,5-Pyridinedicarboxylic acid, 4-(2,3-dichlorophenyl)-2,6-dimethyl-, 2-hydroxyethyl methyl ester	Ketamine
5-Amino-6-(5'-phosphoribosylamino)uracil	Ritanserin
Kaempferol 3-gentiobioside-7-glucuronide	2-(Acetamidomethylene)succinate
(Â±)-Mucronulatol	5-O-Methyleriodictyol 7-glucosyl-(1->4)-galactoside
2-Anthramine	Dihydrosanguinarine

Hexandraside C	Fucoxanthin
Propaphos	Monotropein
flaviolin	7-Methylguanine
CCT018159	Anatoxin a(s)
Cloxyfonac	17beta-Estradiol-3-(beta-D-glucuronide) 17-sulfate
Lonchocarpenin	3,4-Dihydroxyphenylglycol O-sulfate
Met Met Met	Ile Ile Asp
Norselic acid E	Isopentyl pyrophosphate
Calomelanol I	LTB4-d4
1-Ethylcarbamyl-4-methylpiperazine	PtdIns-(4)-P1 (1,2-dioctanoyl)
Gardenoside	Sanaganone
JP104	Tyr-Phe-OH
D and C Red No. 9	AG-183
Diisopropyl adipate	Istamycin A2
Hydroxymelphalan	Chamanetin
Ser Asp Gly	Glu Pro
Asp Cys Asp	Phe4Cl-Abu-OH
13H-Dibenzo[a,g]fluorene	Argiotoxin 636
dTDP-D-mycaminose	Cys Glu Phe
Asn Ser Tyr	Istamycin A3
Cefuroxime axetil	PA(16:0/0:0)[cyclic]
Cys Ser His	Sulfamethoxazole glucuronide
AminoDAHP	2-Propylsuccinic acid
Boc-Pro-Phe(NMe)-Gly-OMe	Terbufos
Captopril disulfide	4-Chloro-17alpha-methyl-17beta-hydroxy-4-androsten-3-one
Tyr-Tyr-OH	bromodiphenhydramine
9-hydroperoxy-12,13-dihydroxy-10-octadecenoic acid	Cholestane-3,7,12,25-tetrol-3-glucuronide
Brompheniramine (didemethylated)	Piromidic Acid
Chlorotoluron	Lupanyl Acid
3,17beta-Diacetoxyestra-1,3,5(10)-trien-6-one	2-Hydroxyflemichapparin C
4-(Cytidine 5'-diphospho)-2-C-methyl-D-erythritol	Thr-Nap-OH
UDP-N-acetyl-D-galactosamine	3-Chlorotyrosine
Deuteroporphyrin IX	4-Hydroxylevamisole
N-Acetyl-8-O-methyl-Neuraminic acid	AG-126
PG(20:4(5Z,8Z,11Z,14Z)/14:0)	Benfluralin
Phorate	Ile Glu
Gln Pro Thr	Nympholide A
PS(22:4(7Z,10Z,13Z,16Z)/22:1(11Z))	Triazophos
Pseudobaptigenin 7-O-glucoside	Benzo[a]pyrene-cis-4,5-dihydrodiol

4-(4-Chlorophenyl)-4-hydroxypiperidine	Acetylcorynoline
Histamine	Dubamine
Indinavir-N-oxide	Haplophyllidine
Glu His Thr	Pectolinarigenin 7-glucuronide
Gly Asn Phe	p-Nitroglutethimide
Aflatoxin M1	2-carboxy-Pyrimidine
Anthemis glycoside A	Apigenin 6-C-glucosyl-7-O-(6-malyl-glucoside)
Cys Ser Ser	Trp Cys Cys
Desmethylnaproxen-6-O-sulfate	17Î²-Estradiol 17-(Î²-D-glucuronide)
MID42365:(20S)-1Î±,20,25-trihydroxy-24a,24b-dihomovitamin D3 / (20S)-1Î±,20,25-trihydroxy-24a,24b-diho	6,8-Di-C-arabionopyranosylluteolin
12alpha-(Chloromethyl)-12-hydroxy-pregn-4-ene-3,20-dione	Homoserine lactone
Bz-Arg-OEt	Lunamarine
Lys Ser Gly	Thiazafluron
Phosphoribosylamine	Val Pro Val
Tricetin 7,3',4',5'-trimethyl eter 5-xylosyl-(1->2)-rhamnoside	Meptazinol glucuronide
3-Hydroxypromazine glucuronide	Tamsulosin
Deoxyadenosine diphosphate (dADP)	N-Acetylphenylalanine beta-naphthyl ester
His Ala His	STF-62247
Rutaecarpine	Ikarisoside B
Thiofanox	Quercetin 3-(2"-caffeylglucuronide)
Triethylene glycol diglycidyl ether	Withasomnine
cis-1,2-Dihydroxy-1,2-dihydrodibenzothiophene	(+)-Tephrorin A
Cortisol 21-sulfate	Albafuran A
Abu-Abu-OH	ICI D1694
Ammoresinol	Lys Gln Lys
Aplysinal	Isorhamnetin 3-glucosyl-(1->6)-galactoside-7-glucoside
Deoxycoformycin	1-Deoxy-D-xylulose
Adenosine5'-monophosphate	Leu Glu Gln
Phe4Cl-Gly-OH	Phe-Phe4Cl-OH
Glucosamine 6-sulfate	Caulerpin
CAY10415	Polydine
CAY10554	Arg Asp Gly
Acronycidine	Glu Glu Met
Lycodine	Asn His Ile
5-Hydroxy-7,4'-dimethoxy-4-phenylcoumarin 5-O-galactoside	b-D-Glucopyranuronic acid
5-Hydroxythiophene-2-carbonyl-CoA	Fluprostenol Lactone Diol
Phaclofen	Gnidicin

N-Formylnorfloxacin	PG(21:0/21:0)
Veronicafolin 3-O-sulfate	Stictic Acid
11-O-Demethylpradinone II	Mesoporphyrin IX
7-Methoxy-5,6:3',4'-bis(methylenedioxy)flavone	Pro Thr Pro
Boc-Pro-DVal(NMe)-Val-OMe	Anthemis glycoside B
S,S,S,-Tributylphosphorotrithioate	Caryoptin
9-Fluoro-16alpha-hydroxyandrost-4-ene-3,11,17-trione	Gelsemicine
Asn Pro Gln	Quercetin 3,3'-dimethyl ether 4'-(2-methylbutyrate)
Chikusetsusaponin III	Phthalocyanine
Dextrin	3-O-L-rhamnosyl-3-hydroxydecanoyl-3-hydroxydecanoic acid
Indanofan	Pyricarbate
Magnoloside C	2-Deoxy-2,3-dehydro-N-acetylneuraminic acid
Arctiopicrin	4-Chloro-N1-methyl-N1-(4-carboxy-2-hydroxy-2-methylbutyl)-m-benzenedisulfonamide glucuronide
SB 243213	Alcophosphamide
5,6,7,4'-Tetrahydroxyflavanone 6,7-diglucoside	Asp Asn Glu
Buclizine	Bz-Arg-OEt
Chartreusin	Abscisic acid glucose ester
Crotoxyphos	PI(14:1(9Z)/0:0)
Allidochlor	S-Nitroso-L-glutathione
gypsogenin 3-O-rhamnosylglucosiduronic acid	2,4-Dichlorobenzoyl-CoA
N-(6-Oxo-6H-dibenzo[b,d]pyran-3-yl)-2,2,2-trifluoroacetamide	2-hydroxy-tricosanoic acid
PE(O-18:0/18:0)[U]	Aklomide
Asp Asp Cys	Frutinone A
GW 0742	Oxoproflaxacin
Hydroxycitric acid	Catalposide
Ikarisoside C	Clorazepate
Nilutamide	Ibogaine
Pregnanediol-3-glucuronide	S-(Formylmethyl)glutathione
Cinobufagin	Temocaprilat
Kaempferol 3-(6''-sulfatogetiobioside)	Triuvaretin
Benzofenap	Pinnatifinoside A
Sulindac sulfone	2'-Deoxycytidine diphosphate (dCDP)
Flufenamic Acid	Hexadecyl Acetyl Glycerol
Pinocembrin 7-rhamnosylglucoside	Asebotin
3-Deoxy-D-glycero-D-galacto-2-nonulosonic acid	Hydroxyacetic acid uroporphyrin III

malonyl-CoA	Phenylboronic acid
PF-3845	Hydroxymethylbilane
5-Hydroxypyrazinoic acid	Mitotane
Calomelanol C	Protopine
11Z,19-Eicosadienyl acetate	4-Quinolinemethanol, 2,8-bis(trifluoromethyl)-
3-Hydroxy-7-acetylaminonitrazepam	Halfordinol
Arg Arg	10-(beta-Dimethylaminopropionyl)phenothiazine
Idebenone Metabolite (Benzenebutanoic acid, 2-hydroxy-3,4-dimethoxy-6-methyl-5-(sulfooxy)-)	Clofibrate
Integerrenine	Eseramine
Parathion	Ginkgolide J
Deacetoxycephalosporin C	Kanokoside A
2-Methoxyxanthone	Watasenia luciferin
Kanzakiflavone 1	Deacetoxycephalosporin C
Phe4Cl-Ser-OH	Oxazepam glucuronide
Senecionine	Tyr-HoPhe-OH
Fenothiocarb sulfoxide	9,10-dihydroxy-Octadecanedioic acid
N1,N5-Tri-di(hydroxyferuloyl)-N10-sinapoyl-spermidine	Asp Met His
RG-108	2-Nitropyrene
Arg Arg Arg	Isoathyriol
Furamizole	Benorilate
MID73229:(1aalpha,2beta,3alpha,11calpha)-1a,2,3,11c-Tetrahydro-6,11-dimethylbenzo[6,7]phenanthro[3,4	Phenazine-1,6-dicarboxylic acid
4-Hydroxyaminoquinoline N-oxide	Dopexamine
Demethylphylloquinone	Oxadiazon
Pro Pro Leu	S-(Hydroxymethyl)mycothiol
5,8,3',4',5'-Pentahydroxy-3,7-dimethoxyflavone	1-Acetoxypinoresinol
5-Fluorouridine	cholesterol sulfate
6-hydroxy-7Z,9E-Octadecadiene-11,13,15,17-tetraynoic acid	Cys Trp Gln
Asn Tyr Asn	Tomentin 6-glucoside
3beta,21-Dihydroxy-pregna-5,7,9(11)-trien-20-one diacetate	Aklanonic acid methyl ester
Glu Trp Met	albendazole sulfoxide
Grepafloxacin	Mureidomycin A
L-161,982	Thr-Thr-OH
3-methyl-2-quinoxalinone	14-Dihydroxycornestin
Bisindolylmaleimide IV	Cefacetrile
HC Red No. 3	Myricetin 3'-O-(6"-p-coumaroyl)glucoside

Wy 14643	6-Hydroxymyricetin 6,3',5'-trimethyl ether 3-glucoside
5-azauridine	Bis(2-chloro-1-methylethyl)ether
Naringin	Magnoloside C
Pandaroside A	Asn-Asn-OH
Pro Glu Asn	Benzamil
Pseudohypericin	(2-Butylbenzofuran-3-yl)(4-hydroxyphenyl)ketone
Met Asn His	1,5-Naphthalene diisocyanate
Rhodomyrtoxin B	Citalopram aldehyde
1-O-Galloyl-beta-D-glucose	Descinolone
O-Methylpongamol	10-Hydroxydihydrosanguinarine
N6-(1,2-dicarboxyethyl)-AMP	Ala Ile His
Perindopril lactam	O-Desmethyloxotolrestat
3-Methylindolepyruvate	resorcinol sulfoxide
Aspulvinone H	4-Nitro-3-(trifluoromethyl)aniline
Val Ser Arg	Asperuloside tetraacetate
17-hydroxyandrostane-3-glucuronide	Cobalt-precorrin 4
4'-Hydroxy-4-(4-hydroxystyryl)-7-methoxyflavan	GalÎ²1-4GlcÎ²-Sp
Fusicoccin A	Spinatoside
azaperone	4-Nitrophenol-alpha-D-galactopyranoside
Methdilazine	1H-1,2,4-Triazole-1-propanoic acid, 4,5-dihydro-3-(1-hydroxyethyl)-5-oxo-4-(2-phenoxyethyl)-
1H-1,2,4-Triazole-1-propanoic acid, 4,5-dihydro-3-(1-hydroxyethyl)-5-oxo-4-(2-phenoxyethyl)-	Asp-His-OH
5,2',6'-Trihydroxy-7-methoxyflavone	N-(diphenylmethylene) Glycine benzyl ester
5-Amino-1-ribofuranosylimidazole-4-carboxyamide	JP104
Brimonidine	Paclobutrazol
Leucomycin A6	Yersiniabactin
Arg Pro Pro	Chloramphenicol palmitate
3-(2,4-Cyclopentadien-1-ylidene)pregn-4-en-20-one	Melanin
(S)-N-[3-(3,4-Methylenedioxyphenyl)-2-(acetylthio)methyl-1-oxoprolyl]glycine benzyl ester	Clorobiocin
Phe Lys Val	Precorrin 6Y
Thr Arg	His Gly Lys
UDP-2,3-diacetamido-2,3-dideoxy-alpha-D-mannuronate	Jamaicamide A
Angolensic Acid, Methyl Ester	N-Adenylyl-L-phenylalanine

Tuliposide B	7-Hydroxyondansetron sulfate
Tyr Asn Asp	Dehydrodolineone
Zolpidem Metabolite I	Stercurensin
Glucosisymbrin	Calyxin G
Acetylcaranine	FTY720 phenoxy-biotin
Descinolone acetonide	Thioridazine 2,5-disulfone
Farnesyl pyrophosphate	Thysanone
Imazosulfuron	Ziprasidone
Sphingofungin C	N-Fluorenylacetamide
Mefruside	Val Phe Gly
8-Hydroxydaidzein	6-Hydroxy-7-methyl-3',4',5'-trimethoxyaurone 4-O-rhamnoside
CGP 57380	4,4'-Diapophytoene/ Dehydrosqualene
Nanchangmycin	Encecalin
Citalopram-N-Oxide	Leu Cys Phe
Nicofetamide	Decarbamoylgonyautoxin 1
PG(O-20:0/22:6(4Z,7Z,10Z,13Z,16Z,19Z))	Desmethylzopiclone
Quassimarin	Streptidine
Arg Glu Asp	Robinetinidol 3-O-gallate
catchin-4-ol 3-O-beta-D-galactopyranoside	1-Chloro-2,2-bis(4'-chlorophenyl)ethylene
Arg Val Cys	Diphenadione
Crimidine	Sirodesmin H
His Asn His	Phenisopham
Salermide	Premithramycin A1
Flumipropyn	Scutellarein
(3S,7R)-iso-jasmonic acid	JWH 019
N-(3S-hydroxydecanoyl)-L-serine	Patrinoside
3-O-Sulfogalactosylceramide	Benazeprilat
Enoxacin	Catechin 5-O-beta-D-glucopyranoside-4'-Me
5'-Phosphoribosyl-N-formylglycinamide (FGAR)	Omeprazole sulfide
Ancistrotectorine	Apigenin 4'-(2''-feruloylglucuronosyl)-(1->2)-glucuronide
DTP	N-Desalkylflupenthixol
Gly Tyr Lys	Bruceine D
Gallocatchin 3-O-gallate	Isopimpinellin
Alprostadil alfadex	MID42039:(22E)-(25S)-26,26,26-trifluoro-1Î±,25-dihydroxy-22,23-didehydrovitamin D3 / (22E)-(25S)-26,2
Geniposidic acid	Chitobiose
Malaoxon	PG(O-20:0/21:0)
Dextromoramide M4	Ser Gly Pro
Flunarizine	Luteolin 7-(6''-ferulylglucoside)
4'-Desmethylpapaverine sulfate	15-deoxy-Î”12,14-Prostaglandin J2-biotin

Dehydrogriseofulvin	Deoxycytidine
Azukisaponin III	Pro Met Pro
Lactodifucotetraose	Thioridazine
Leu Glu Phe	Bebeerine
Myricetin 3-sambubioside	10-formyldihydrofolate
AG-825	Quassimarin
Asiaticoside	4-Hydroxyalprazolam
Gly Pro Arg	Ser His Trp
S-(N-Hydroxy-N-methylcarbamoyl)glutathione	Magnesium protoporphyrin
N4-Acetylcytidine	12a-Hydroxydolineone
PI(20:4(5Z,8Z,11Z,14Z)/19:0)	8-C-beta-D-Glucofuranosylapigenin 2"-O-acetate
Isoxapyrifop	Trp-Met-OH
Wightin	Quizalofop-P-tefuryl
H-89	Vitamin D3 sulfoconjugate
NS 1608	Deoxypumiloside
Phylloflavan	Cys Asp
Tamsulosin	Fenazaquin
Cytidine diphosphate (CDP)	Integerrenine
Phe Pro Arg	Asn Asp
3,6-Dimethoxyestra-1,3,5(10),6,8-pentaene-17beta-carboxylic acid methyl ester	Diethylcarbamazine
5-O-Methylhoslundin	Artelastochromene
Corynoline	Asp Glu Glu
Pro Cys Asp	Quinoline-3-carboxamides
20-hydroxy N-Arachidonoyl Taurine	GSK264220A
Chlordane	1,2-Dioctanoyl-sn-glycerol
Phe4Cl-Thr-OH	Nizatidine-N-oxide
PS(O-16:0/0:0)	Val Asp Phe
Typhaneoside	GalÎ²1-3[FucÎ±1-4]GlcNAcÎ²-Sp
Ditalimfos	Rhamnocitrin 3-glucosyl-(1->2)-galactoside
Gnetin A	Cetraxate
Arg Asp Asp	N,N-Dihydroxy-L-tyrosine
Tingenone	7-Epiloganin tetraacetate
TG(16:0/18:1(9Z)/20:0)[iso6]	Grantionin
flavonol 3-O-beta-D-glucosyl-(1->2)-beta-D-glucoside	Khayanthone
N-Desmethylclobazam	ABT-869
Lithocholate 3-O-glucuronide	Furano[2",3":6,7]aurone
DG(19:0/20:3(8Z,11Z,14Z)/0:0)[iso2]	Thalassemine
Carboxyltolmetin glucuronide	Deoxyinosine
Robinetinidol-(4alpha->8)-catchin-(6->4alpha)-robinetinidol	2-deoxyecdysone 22-phosphate

Asn Tyr Met	Prodiamine
Deoxyuridine monophosphate (dUMP)	LY293111
Tetrahydrozoline	Terbutaline-1-glucuronide
Val Lys Gly	Gly-Nap-OH
His Pro Pro	PS(18:3(9Z,12Z,15Z)/15:1(9Z))
Gly Val Gln	Okanin 4-methyl ether 4'-O-(6"-O-p-coumaroylglucoside)
Reserpic acid	N,N-Didesmethyltamoxifen
Glu Asp Ile	Phe Gly Ser
Diacerin	Saquinavir
Dianoside A	4-(Methylnitrosamino)-1-(3-pyridyl)-1-butanol glucuronide
O-Desmethyllevomepromazine	Exserohilone
JWH 250	Isorhamnetin 3-rhamnosyl-(1->2)-gentiobiosyl-(1->6)-glucoside
Narcotoline	Thr Glu Phe
Yinyanghuo A	(S)-MG132
PI(22:1(11Z)/18:2(9Z,12Z))	Auraviketone
Glucobrassicin	Thr-Met-OH
Cymarin	Trimidox
UDP-N-acetyl-3-(1-carboxyvinyl)-D-glucosamine	Trp His Met
Vitexin 2"-O-acetyl 4"-O-rhamnoside	Isorhamnetin 3-glucosyl-(1->2)-galactoside-7-glucoside
5-Chrysenecarboxylate	Acacetin 7-O-[6"-O-glucosyl-2"-O-(3'"-acetylrhamnosyl)glucoside
PG(20:4(5Z,8Z,11Z,14Z)/17:0)	Glycine, N-[(3a,5b,7a)-3-hydroxy-24-oxo-7-(sulfooxy)cholan-24-yl]-
Coumestrol	MID84216:(23S,25R)-12alpha,17alpha,23-trihydroxyspirost-5-en-3beta-yl O-alpha-L-rhamnopyranosyl-(1-2
Iristectorigenin A 7-O-gentiobioside	MID66857:alpha-Galactosyl-(1-6)-alpha-galactosyl-(1-6)-alpha-galactosyl-(1-6)-alpha-galactosyl-(1-6)
Lys Ser Glu	Chaetoglobosin A
ZK 168281	Chalconaringenin 4-glucoside
Ramiprilat glucuronide	3-Hydroxypromazine glucuronide
Cys Gly Phe	5,2'-Dihydroxyflavone
Phe Cys Phe	Hallactone B
6-Butyryl-5-hydroxy-4-phenylseselin	Teniposide
Arg Cys Cys	Ala Ser Ala
Norpromazine	Spinochalcone C
Borreverine	3'-Methoxypongapin
GW 7647	Dihydroneopterin phosphate
Hedamycin	Estradiol-17-phenylpropionate

His Gly Ala	Myrtucommulone A
Pinacidil	Hydroxytinidazole glucuronide
Trp Ser	Troglitazone
PI(22:0/0:0)	2'-N-Acetylparomamine
PA(P-16:0/0:0)	Mearnsetin 3-galactosyl-(1->4)-galactoside
PtdIns-(4)-P1 (1,2-dihexanoyl)	Deoxycytosine
Aminopterin	Pentetic acid
HC Toxin	Pro Gly Trp
Lasalocid	Phe4Cl-Thr-OH
Phenothrin	Ser Ile Asp
PS(12:0/0:0)	pravastatin dihydrodiol
Lys Asn Val	Avermectin B1b monosaccharide
Lys Gly Gly	Gemfibrozil, metabolite II
Usambarine	Quercetin 3-(2'''-p-coumarylsambubioside)-7-glucoside
1-Naphthalenesulfonic acid	Ticarcillin
3,7,12-Trioxochola-1,5-dien-24-oic Acid	5,7-Dihydroxy-3',4'-dimethoxy-6,8-dimethylflavone
Amaroswerin	Fluazifop
Asp Asn Glu	Gossypetin 7,4'-dimethyl ether 8-acetate
TEPP	12R-acetoxy-7Z-punaglandin 3
19-hydroxy-Resolvin E1	Ala Asp Pro
Dimepiperate	His Gln Arg
Millettosin	Kaempferol 3-isorhamninoside-7-rhamnoside
p-Hydroxyatorvastatin	Amorphigenol O-vicianoside
11-dehydro-2,3-dinor-TXB2	PG(14:1(9Z)/12:0)
Kanamycin C	17,18-dehydro-clavulone I
Tyr Arg Asn	Coreopsin
Tamibarotene	Rotenonone
TG(16:0/20:1(11Z)/20:5(5Z,8Z,11Z,14Z,17Z))[iso6]	Tilorone
Glu Lys Lys	AM-580
Convoline	Glu Pro Glu
Deltorphin B	Met Asp His
Risperidone	MID42028:26,26,26,27,27,27-hexafluoro-1Î±,24-dihydroxyvitamin D3 / 26,26,26,27,27,27-hexafluoro-1Î±,24
Bendroflumethiazide	2-Dimethylamino-5,6-dimethylpyrimidin-4-ol
Rhynchosin	25-O-(2''-beta-D-glucopyranosyl-beta-D-glucopyranosyl)-25-hydroxy-11E-eicosenoic acid
Cys Gln Cys	Ser Pro Arg
Palmitoyl glucuronide	Minosaminomycin

Cylindrospermopsin	Nitrovin
S-(2-Chloroacetyl)glutathione	NU 7026
3-Deoxy-D-manno-octulosonate 8-phosphate	(7R)-7-(4-Carboxybutanamido)cephalosporanate
DL-9-hydroxy stearic acid	Phe Met Cys
Docosanamide	D-Fructofuranose 1,2':2,3'-dianhydride
Neriifolin	Hematoporphyrin
Testosterone phenylpropionate	Sialyl-Lewis X
Lithocholic acid taurine conjugate	Leu Arg Cys
PS(22:1(11Z)/22:6(4Z,7Z,10Z,13Z,16Z,19Z))	Epigallocatechin 3-O-(3-O-methylgallate)
Thr Trp Pro	abrusoside A
Triazolam	Precorrin 3A
4",5"-Dihydro-3,5,3',4'-tetrahydroxy-4",6",6"-trimethylpyrano[2,3:7,8]flavone	Thr Asp Ile
9-Fluoro-11beta,16alpha-dihydroxypregn-4-ene-3,20-dione	Glaucolide B
Gln Phe Phe	p-[N-Propyl-N-(3-hydroxypropyl)sulfamoyl]benzoic acid
7-Methyl-2-hydroxy-6-oxoocta-2,4-dienoate	TG(17:2(9Z,12Z)/18:3(9Z,12Z,15Z)/20:5(5Z,8Z,11Z,14Z,17Z))[iso6]
Fenson	Metyrapone
Aureol	Avermectin A2b aglycone
Gln Gln Arg	Desmethylloperamide
Hydroquinine	Tyr Met Cys
MID47232:6a,12b-Dihydro-3,10,11,12-tetrahydroxy-6-(3,4,5-trihydroxyphenyl)-[2]benzopyrano[3,4-c]benz	Tyr-Asn-OH
Lys His Ser	1-[2-Bromo-1-(4-chlorophenyl)ethenyl]-2-chlorobenzene
Lys Trp Lys	Arachidoyl glycine
3-Hydroxyethylbacteriochlorophyllide a	5,2',4',5'-Tetrahydroxy-3-(3-hydroxy-3-methylbutyl)-6",6"-dimethylpyrano[2",3":7,8]flavone
8-Hydroxyluteolin 7-xyloside	His-Nap-OH
PE(20:3(5Z,8Z,11Z)/20:1(11Z))	Demeton-S-methylsulphon
Bifonazole	Met-His-OH
Heneicosanyl oleate	Bonafousine
PS(22:6(4Z,7Z,10Z,13Z,16Z,19Z)/12:0)	Hydramethylnon
JWH 210	TG(16:1(9Z)/17:0/18:0)[iso6]
Leu Pro Pro	Cys His His
Tubulosine	Quercetin 3-(2'''-ferulylsambubioside)-7-glucoside
Carmoisine	
Epithienamycin C	

Mezlocillin metabolite (4-Thiazolidinecarboxylic acid, 5,5-dimethyl-2-[[[[[[3-(methylsulfonyl)-2-oxo	
Aspidospermine	
3-alpha-androstanediol glucuronide	
(+)-Acutifolin A	
(6R)-vitamin D2 6,19-sulfur dioxide adduct / (6R)-ergocalciferol 6,19-sulfur dioxide adduct	
Arg Asp	
Indoxacarb	
S-Acetylphosphopantetheine	
Triflumizole	
3,12-Dioxochola-1,4,9(11)-trien-24-oic Acid	
PE(22:5(7Z,10Z,13Z,16Z,19Z)/24:0)	
3,3'-Dimethoxybenzidine-4,4'-diisocyanate	
Dilazep	
3-Methoxy-4-hydroxyphenylethylene glycol	
Morphine 3-sulfate	
Nedaplatin	
Cys Ser Met	
Uvaretin	
11,11,11,12,12-Pentafluoro-9Z-dodecenyl acetate	
25-O-(2''-beta-D-glucopyranosyl-beta-D-glucopyranosyl)-25-hydroxy-11E-eicosenoic acid	
6-(2-Amino-2-carboxyethyl)-7,8-dioxo-1,2,3,4,7,8-hexahydroquinoline-2,4-dicarboxylate	
Riluzolamide glucuronide	
Lys Cys Trp	
Halistanol sulfate	
Haloxyfop methyl	
Pradimicin A	
Chlorochrymorin	
Fisetinidol-4beta-ol 3,4,7,3',4'-pentamethyl ether	
Flupoxam	
TG(16:0/17:1(9Z)/17:1(9Z))[iso3]	
7-O-Methyllicoricidin	
Bryaquinone	
Clionamide	
Cilastatin	
N-palmitoyl methionine	
Ser Lys Pro	
Premithramycin A2'	
1-deoxy-1-[methyl[3-phenyl-3-[4-(trifluoromethyl)phenoxy]propyl]amino]-b-D-Glucopyranuronic acid	
Chlorflavonin	

DG(O-16:0/18:1(9Z))	
Urdamycin F	
LysoPE(0:0/18:1(11Z))	
N-dodecanoyl-L-Homoserine lactone-3-hydrazone-fluorescein	
(Â±)12,13-DiHOME	
TG(17:1(9Z)/20:3(8Z,11Z,14Z)/22:1(13Z))[iso6]	
Citalopram aldehyde	
Dihydropicromycin	
5â€²-Deoxy-5â€²-(methylthio)adenosine	
Cotylenin F	
Isopentenyladenine-9-N-glucoside	
1-Amino-2,4-dibromoanthraquinone	
Hydroxysaquinavir M3	
3-O-Demethylamorphigenin	
Ala Asn Leu	
N-oleoyl glutamine	
Cinnarizine	
Mestranol	
SU 6656	
Diclobutrazol	
Tyr Ile Asn	
PG(22:6(4Z,7Z,10Z,13Z,16Z,19Z)/22:2(13Z,16Z))	
Dehydropachyrrhizone	
Pheophorbide a	
Scopoloside II	
Arg Pro Met	
8-Allyl-2-phenyl-8H-1,3a,8-triaza-cyclopenta[a]indene	
Istamycin A3	
Anandamide (20:2, n-6)	
JWH 018	
Cycloartomunoxanthone	
Ser His Cys	
Irigenin, Dibenzyl Ether	
Boscalid	
1,2,3,7,8,9-Hexachlorodibenzofuran	
Goniodomin A	
Pro Ala Arg	
Thiethylperazine	
Isorhamnetin 3-glucuronide-7-sulfate	

PA(P-20:0/22:1(11Z))	
Parishin C	
4'-Demethylepipodophyllotoxin	
PI(16:0/20:5(5Z,8Z,11Z,14Z,17Z))	
Cob(I)yrinate a,c diamide	
Fenofibrate	
X-206	
Formononetin 7-O-(6''-acetylglcoside)	
4-Pyridinol, 2-[[[5-(difluoromethoxy)-1H-benzimidazol-2-yl]sulfinyl]methyl]-3-methoxy-	
Fenirofibrate	
Myxalamid C	
N-Octadecyl-N'-propyl-sulfamide	
Acacetin 7-O-[6''-O-glucosyl-2''-O-(3'''-acetylrhamnosyl)glucoside	
Etorphine	
PS(O-16:0/18:0)	
Ala-Tyr-OH	
PS(12:0/12:0)[U]	
Isomucronulator 7-O-glucoside	
PtdIns-(3)-P1 (1,2-dioctanoyl)	
