# Supplementary material for: Alternative Growth Promoters Modulate Broiler Gut Microbiome and Enhance Body Weight Gain
Source: Front Microbiol. 2017 Oct 26;8:2088. doi: 10.3389/fmicb.2017.02088 (PMC5662582; doi:10.3389/fmicb.2017.02088)

Supplementary Material

Alternative growth promoters modulate broiler gut microbiome and enhance body weight gain

Serajus Salaheen, Seon-Woo Kim, Bradd J. Haley, Jo Ann S. Van Kessel, Debabrata Biswas

*** Correspondence:** Debabrata Biswas: dbiswas@umd.edu

Supplementary Figure 2: Differential composition of chicken cecal microbiota: bacterial distribution at genus level in individual samples.

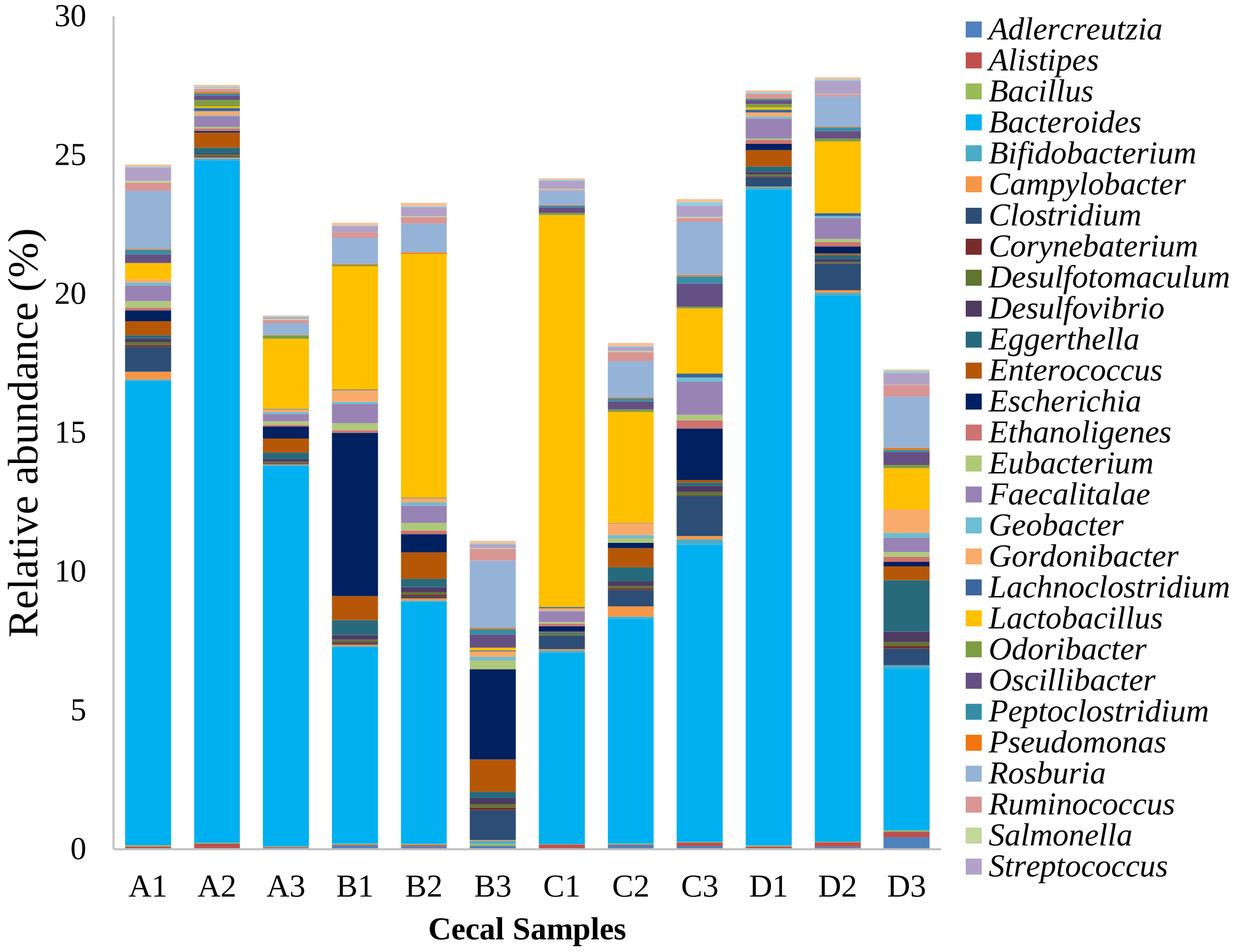

Supplement: Supplementary file 3 [file Data_Sheet_3.PDF]
