# Supplementary material for: Alternative Growth Promoters Modulate Broiler Gut Microbiome and Enhance Body Weight Gain
Source: Front Microbiol. 2017 Oct 26;8:2088. doi: 10.3389/fmicb.2017.02088 (PMC5662582; doi:10.3389/fmicb.2017.02088)
Supplement: Supplementary file 4 [file Data_Sheet_4.PDF]

Supplementary Material

Alternative growth promoters modulate broiler gut microbiome and enhance body weight gain

Serajus Salaheen, Seon-Woo Kim, Bradd J. Haley, Jo Ann S. Van Kessel, Debabrata Biswas

*** Correspondence:** Debabrata Biswas: dbiswas@umd.edu

Supplementary Table 2: Core resistome of chickens from group A with relative abundances of ARGs in individual samples. Relative abundance was adjusted to abundance per 10 million sequences.

ARG groups	ARGs	Relative abundances		
		A1	A2	A3
Aminocoumarin resistance	aminocoumarin resistant alaS [Escherichia coli str. K-12 substr. MG1655]	10	2	3
Aminoglycoside resistance	APH(2")-Ig [Campylobacter coli CVM N29710]	22	5	9
Aminoglycoside resistance	APH(3')-IIIa [Campylobacter coli CVM N29710]	190	124	72
Aminoglycoside resistance	APH(2")-IVa [Enterococcus casseliflavus]	3	2	1
Aminoglycoside resistance	AAC(6')-Ip [Escherichia coli]	14	14	4
Aminoglycoside resistance	APH(2")-IIa [Escherichia coli]	6	12	4
Aminoglycoside resistance	ANT(6)-Ia [Exiguobacterium sp. S3-2]	7	14	8
Aminoglycoside resistance	AAC(6')-Ie-APH(2")-Ia [Staphylococcus aureus]	2	1	1
Aminoglycoside resistance	aad(6) [Streptococcus oralis]	46	58	35
Bacitracin resistance protein	bacA [Escherichia coli str. K-12 substr. MG1655]	3	1	4
Beta-lactamase	CcrA beta-lactamase [Bacteroides fragilis]	140	312	338
Chloramphenicol resistance	cat [Vibrio anguillarum]	12	7	2
Efflux pump confering resistance	acrB [Enterobacter cloacae]	8	2	8
Efflux pump confering resistance	cpxA [Escherichia coli O157:H7 str. Sakai]	8	1	6
Efflux pump confering resistance	cpxR [Escherichia coli O157:H7 str. Sakai]	3	1	3

Efflux pump confering resistance	acrE [Escherichia coli str. K-12 substr. MG1655]	6	1	4
Efflux pump confering resistance	acrF [Escherichia coli str. K-12 substr. MG1655]	13	2	13
Efflux pump confering resistance	emrB [Escherichia coli str. K-12 substr. MG1655]	4	1	8
Efflux pump confering resistance	mdtA [Escherichia coli str. K-12 substr. MG1655]	5	1	3
Efflux pump confering resistance	mdtB [Escherichia coli str. K-12 substr. MG1655]	11	2	9
Efflux pump confering resistance	mdtC [Escherichia coli str. K-12 substr. MG1655]	10	2	10
Efflux pump confering resistance	mdtF [Escherichia coli str. K-12 substr. MG1655]	14	3	13
Efflux pump confering resistance	mdtH [Escherichia coli str. K-12 substr. MG1655]	4	1	5
Efflux pump confering resistance	mdtL [Escherichia coli str. K-12 substr. MG1655]	8	1	7
Efflux pump confering resistance	mdtM [Escherichia coli str. K-12 substr. MG1655]	6	1	4
Efflux pump confering resistance	mfd [Escherichia coli str. K-12 substr. MG1655]	11	2	10
Efflux pump confering resistance	msbA [Escherichia coli str. K-12 substr. MG1655]	6	1	5
Efflux pump confering resistance	YojI [Escherichia coli str. K-12 substr. MG1655]	5	1	5
Efflux pump confering resistance	acrD [Escherichia coli str. K-12 substr. W3110]	13	2	12
Efflux pump confering resistance	baeS [Escherichia coli str. K-12 substr. W3110]	5	1	5
Efflux pump confering resistance	emrA [Escherichia coli str. K-12 substr. W3110]	4	1	4
Efflux pump confering resistance	mdtE [Escherichia coli str. K-12 substr. W3110]	6	1	4
Efflux pump confering resistance	mdtO [Escherichia coli str. K-12 substr. W3110]	9	2	7
Efflux pump confering resistance	mdtP [Escherichia coli str. K-12 substr. W3110]	11	1	8
Efflux pump confering resistance	emrY [Escherichia coli]	1	1	1
Efflux pump confering resistance	mdfA [Escherichia coli]	4	1	2
Efflux pump confering resistance	tolC [Escherichia coli]	5	1	7
Efflux pump confering resistance	acrA [Klebsiella pneumoniae subsp. pneumoniae HS11286]	1	1	1
Efflux pump confering resistance	sav1866 [Staphylococcus aureus subsp. aureus COL]	1	2	1
Fluoroquinolone resistance	patA [Escherichia coli str. K-12 substr. MG1655]	4	1	8

Glycopeptide antibiotics resistance	vanV [Enterococcus faecalis V583]	30	14	12
Glycopeptide antibiotics resistance	vanG [Enterococcus faecalis]	5	1	2
Glycopeptide antibiotics resistance	vanRG [Enterococcus faecalis]	7	2	3
Glycopeptide antibiotics resistance	vanRL [Enterococcus faecalis]	0	1	1
Glycopeptide antibiotics resistance	vanSG [Enterococcus faecalis]	4	2	3
Glycopeptide antibiotics resistance	vanRD [Enterococcus faecium]	1	1	2
Glycopeptide antibiotics resistance	vanRC [Enterococcus gallinarum]	1	1	1
Glycopeptide antibiotics resistance	vanSC [Enterococcus gallinarum]	2	2	2
Macrolide-Lincosamide-StreptograminB resistance	ErmB [Enterococcus faecalis]	2	2	2
Macrolide-Lincosamide-StreptograminB resistance	vatE [Enterococcus faecium]	4	10	24
Macrolide-Lincosamide-StreptograminB resistance	mefA [Exiguobacterium sp. S3-2]	2	3	2
Macrolide-Lincosamide-StreptograminB resistance	ErmG [Lysinibacillus sphaericus]	1	6	1
Macrolide-Lincosamide-StreptograminB resistance	lnuC [Streptococcus agalactiae]	116	178	167
Macrolide-Lincosamide-StreptograminB resistance	ErmB [Streptococcus pyogenes]	1	6	1
Polymyxin resistance	arnA [Escherichia coli str. K-12 substr. MG1655]	7	1	6
Polymyxin resistance	PmrE [Escherichia coli str. K-12 substr. MG1655]	10	13	12
Polymyxin resistance	PmrB [Escherichia coli str. K-12 substr. W3110]	5	1	5
Polymyxin resistance	PmrC [Escherichia coli str. K-12 substr. W3110]	8	1	5
Streptothricin resistance	sat-4 [Campylobacter coli]	42	62	34
Sulfonamide resistance	leuO [Escherichia coli str. K-12 substr. MG1655]	4	1	3
Tetracycline resistance	tetQ [Bacteroides fragilis]	132	496	460
Tetracycline resistance	tetW [Bifidobacterium longum]	1865	1589	1524
Tetracycline resistance	tet44 [Campylobacter fetus subsp. fetus]	77	69	42
Tetracycline resistance	tetO [Campylobacter jejuni]	91	69	21

Tetracycline resistance	tet32 [Clostridiaceae bacterium K10]	93	56	24
Tetracycline resistance	tetM [Clostridium difficile 630]	2	2	3
Tetracycline resistance	tetS [Listeria monocytogenes]	2	2	3
Tetracycline resistance	tet40 [Streptococcus suis]	21	39	33
Tetracycline resistance	Streptomyces cinnamoneus EF-Tu mutants conferring resistance to elfamycin [Streptomyces cinnamoneus]	137	142	133

Supplementary Table 3. Core resistome of chickens from group B with relative abundances of ARGs in individual samples. Relative abundance was adjusted to abundance per 10 million sequences.

ARG groups	ARGs	Relative abundance		
		B1	B2	B3
Aminocoumarin resistance	aminocoumarin resistant alaS [Escherichia coli str. K-12 substr. MG1655]	17	4	50
Aminocoumarin resistance	aminocoumarin resistant cysB [Escherichia coli str. K-12 substr. MG1655]	80	13	8
Aminoglycoside resistance	APH(2")-Ig [Campylobacter coli CVM N29710]	9	7	9
Aminoglycoside resistance	APH(3')-IIIa [Campylobacter coli CVM N29710]	490	601	163
Aminoglycoside resistance	ANT(6)-Ib [Campylobacter fetus subsp. fetus]	3	2	25
Aminoglycoside resistance	ANT(6)-Ib [Campylobacter jejuni]	383	259	101
Aminoglycoside resistance	kdpE [Escherichia coli str. K-12 substr. MG1655]	18	6	11
Aminoglycoside resistance	AAC(6')-Ip [Escherichia coli]	19	32	21
Aminoglycoside resistance	APH(2")-IIa [Escherichia coli]	19	36	13
Aminoglycoside resistance	ANT(6)-Ia [Exiguobacterium sp. S3-2]	54	44	9
Aminoglycoside resistance	AAC(6')-Ie-APH(2")-Ia [Staphylococcus aureus]	4	6	2
Aminoglycoside resistance	aad(6) [Streptococcus oralis]	249	265	56
Aminoglycoside resistance	ANT(2")-Ia [uncultured bacterium]	3	1	1
Bacitracin resistance protein	bacA [Escherichia coli str. K-12 substr. MG1655]	26	7	15
Beta-lactamase	robA [Enterobacter cloacae]	14	2	6
Efflux pump confering resistance	acrA [Enterobacter cloacae]	11	2	6
Efflux pump confering resistance	acrB [Enterobacter cloacae]	71	16	38
Efflux pump confering resistance	emrD [Escherichia coli B185]	34	7	23
Efflux pump confering resistance	cpxA [Escherichia coli O157:H7 str. Sakai]	54	12	40
Efflux pump confering resistance	cpxR [Escherichia coli O157:H7 str. Sakai]	26	5	16
Efflux pump confering resistance	evgA [Escherichia coli O157:H7 str. Sakai]	2	1	1
Efflux pump confering resistance	H-NS [Escherichia coli O157:H7 str. Sakai]	4	1	1
Efflux pump confering resistance	gadW [Escherichia coli O25b:H4]	10	2	5
Efflux pump confering resistance	acrE [Escherichia coli str. K-12 substr. MG1655]	32	9	21
Efflux pump confering resistance	acrF [Escherichia coli str. K-12 substr. MG1655]	82	17	50

Efflux pump confering resistance	acrS [Escherichia coli str. K-12 substr. MG1655]	11	2	5
Efflux pump confering resistance	adiY [Escherichia coli str. K-12 substr. MG1655]	16	3	7
Efflux pump confering resistance	emrB [Escherichia coli str. K-12 substr. MG1655]	46	11	30
Efflux pump confering resistance	emrR [Escherichia coli str. K-12 substr. MG1655]	17	3	6
Efflux pump confering resistance	evgS [Escherichia coli str. K-12 substr. MG1655]	22	7	12
Efflux pump confering resistance	gadE [Escherichia coli str. K-12 substr. MG1655]	9	3	2
Efflux pump confering resistance	mdtA [Escherichia coli str. K-12 substr. MG1655]	31	6	20
Efflux pump confering resistance	mdtB [Escherichia coli str. K-12 substr. MG1655]	91	17	66
Efflux pump confering resistance	mdtC [Escherichia coli str. K-12 substr. MG1655]	82	21	58
Efflux pump confering resistance	mdtD [Escherichia coli str. K-12 substr. MG1655]	36	6	26
Efflux pump confering resistance	mdtF [Escherichia coli str. K-12 substr. MG1655]	95	24	61
Efflux pump confering resistance	mdtH [Escherichia coli str. K-12 substr. MG1655]	30	6	21
Efflux pump confering resistance	mdtL [Escherichia coli str. K-12 substr. MG1655]	43	8	24
Efflux pump confering resistance	mdtM [Escherichia coli str. K-12 substr. MG1655]	48	10	26
Efflux pump confering resistance	mfd [Escherichia coli str. K-12 substr. MG1655]	104	21	57
Efflux pump confering resistance	msbA [Escherichia coli str. K-12 substr. MG1655]	40	9	19
Efflux pump confering resistance	YojI [Escherichia coli str. K-12 substr. MG1655]	36	9	20
Efflux pump confering resistance	acrD [Escherichia coli str. K-12 substr. W3110]	96	21	60
Efflux pump confering resistance	baeR [Escherichia coli str. K-12 substr. W3110]	22	7	11
Efflux pump confering resistance	baeS [Escherichia coli str. K-12 substr. W3110]	42	7	26
Efflux pump confering resistance	CRP [Escherichia coli str. K-12 substr. W3110]	22	3	11
Efflux pump confering resistance	emrA [Escherichia coli str. K-12 substr. W3110]	38	8	27
Efflux pump confering resistance	emrE [Escherichia coli str. K-12 substr. W3110]	4	2	2
Efflux pump confering resistance	gadX [Escherichia coli str. K-12 substr. W3110]	11	2	5
Efflux pump confering resistance	marA [Escherichia coli str. K-12 substr. W3110]	6	1	3

Efflux pump confering resistance	mdtE [Escherichia coli str. K-12 substr. W3110]	41	9	26
Efflux pump confering resistance	mdtG [Escherichia coli str. K-12 substr. W3110]	34	7	18
Efflux pump confering resistance	mdtN [Escherichia coli str. K-12 substr. W3110]	32	4	21
Efflux pump confering resistance	mdtO [Escherichia coli str. K-12 substr. W3110]	68	18	47
Efflux pump confering resistance	mdtP [Escherichia coli str. K-12 substr. W3110]	60	14	47
Efflux pump confering resistance	emrK [Escherichia coli]	8	2	5
Efflux pump confering resistance	emrY [Escherichia coli]	14	4	5
Efflux pump confering resistance	fyuA [Escherichia coli]	24	6	7
Efflux pump confering resistance	mdfA [Escherichia coli]	32	6	17
Efflux pump confering resistance	tolC [Escherichia coli]	52	10	26
Efflux pump confering resistance	acrA [Klebsiella pneumoniae subsp. pneumoniae HS11286]	11	1	6
Efflux pump confering resistance	mexB [Pseudomonas aeruginosa]	2	1	1
Efflux pump confering resistance	sdiA [Salmonella enterica subsp. enterica serovar Typhimurium str. LT2]	3	1	1
Efflux pump confering resistance	mdtK [Salmonella enterica subsp. enterica serovar Typhimurium]	5	1	2
Efflux pump confering resistance	sav1866 [Staphylococcus aureus subsp. aureus COL]	2	3	2
Efflux pump confering resistance	rosB [Yersinia enterocolitica (type O:8)]	10	2	7
Fluoroquinolone resistance	patA [Escherichia coli str. K-12 substr. MG1655]	47	9	31
Glycopeptide antibiotics resistance	VanRI [Desulfitobacterium hafniense]	1	1	1
Glycopeptide antibiotics resistance	vanV [Enterococcus faecalis V583]	20	26	18
Glycopeptide antibiotics resistance	vanG [Enterococcus faecalis]	6	4	3
Glycopeptide antibiotics resistance	vanRG [Enterococcus faecalis]	6	9	3
Glycopeptide antibiotics resistance	vanSG [Enterococcus faecalis]	6	8	7
Glycopeptide antibiotics resistance	vanYG1 [Enterococcus faecalis]	2	4	2
Glycopeptide antibiotics resistance	vanRA [Enterococcus faecium]	3	2	1
Glycopeptide antibiotics resistance	vanRC [Enterococcus gallinarum]	1	1	1

Glycopeptide antibiotics resistance	vanSC [Enterococcus gallinarum]	4	5	4
Macrolide-Lincosamide-StreptograminB resistance	ErmG [Bacteroides thetaiotaomicron]	2	11	12
Macrolide-Lincosamide-StreptograminB resistance	ErmB [Clostridium difficile 630]	2	2	2
Macrolide-Lincosamide-StreptograminB resistance	ErmB [Enterococcus faecium]	3	4	3
Macrolide-Lincosamide-StreptograminB resistance	vatE [Enterococcus faecium]	20	56	1
Macrolide-Lincosamide-StreptograminB resistance	mefA [Exiguobacterium sp. S3-2]	7	6	7
Macrolide-Lincosamide-StreptograminB resistance	ErmB [Lactobacillus fermentum]	5	9	6
Macrolide-Lincosamide-StreptograminB resistance	ErmG [Lysinibacillus sphaericus]	15	1	1
Macrolide-Lincosamide-StreptograminB resistance	ErmB [Plasmid pIP1527]	6	9	1
Macrolide-Lincosamide-StreptograminB resistance	lnuC [Streptococcus agalactiae]	465	357	224
Macrolide-Lincosamide-StreptograminB resistance	ErmB [Streptococcus pyogenes]	1	2	12
Polymyxin resistance	arnA [Escherichia coli str. K-12 substr. MG1655]	54	12	33
Polymyxin resistance	PmrE [Escherichia coli str. K-12 substr. MG1655]	18	9	11
Polymyxin resistance	PmrF [Escherichia coli str. K-12 substr. MG1655]	22	4	12
Polymyxin resistance	PmrB [Escherichia coli str. K-12 substr. W3110]	43	10	27
Polymyxin resistance	PmrC [Escherichia coli str. K-12 substr. W3110]	53	11	32
Streptothricin resistance	sat-4 [Campylobacter coli]	249	289	53
Sulfonamide resistance	leuO [Escherichia coli str. K-12 substr. MG1655]	19	3	9
Sulphonamide resistance	sul2 [Vibrio cholerae]	1	2	1
Sulphonamide resistance	sul1 [Vibrio fluvialis]	16	2	3
Tetracycline resistance	tetW [Bifidobacterium longum]	2713	2428	2268
Tetracycline resistance	tet44 [Campylobacter fetus subsp. fetus]	48	152	443
Tetracycline resistance	tetO [Campylobacter jejuni]	111	132	49
Tetracycline resistance	tet32 [Clostridiaceae bacterium K10]	78	104	52
Tetracycline resistance	tetM [Clostridium difficile 630]	1	5	2
Tetracycline resistance	tetS [Listeria monocytogenes]	1	1	2
Tetracycline resistance	tet40 [Streptococcus suis]	72	59	88
Tetracycline resistance	tetC [uncultured bacterium]	1	1	2

Supplementary Table 4. Core resistome of chickens from group C with relative abundances of ARGs in individual samples. Relative abundance was adjusted to abundance per 10 million sequences.

ARG groups	ARGs	Relative abundance		
		C1	C2	C3
Aminocoumarin resistance	aminocoumarin resistant alaS [Escherichia coli str. K-12 substr. MG1655]	7	4	28
Aminocoumarin resistance	aminocoumarin resistant cysB [Escherichia coli str. K-12 substr. MG1655]	2	1	9
Aminoglycoside resistance	APH(2")-Ig [Campylobacter coli CVM N29710]	9	18	16
Aminoglycoside resistance	APH(3')-IIIa [Campylobacter coli CVM N29710]	93	101	98
Aminoglycoside resistance	ANT(6)-Ib [Campylobacter jejuni]	105	1	229
Aminoglycoside resistance	APH(2")-IVa [Enterococcus casseliflavus]	2	5	3
Aminoglycoside resistance	kdpE [Escherichia coli str. K-12 substr. MG1655]	2	2	8
Aminoglycoside resistance	AAC(6')-Ip [Escherichia coli]	10	12	17
Aminoglycoside resistance	APH(2")-IIa [Escherichia coli]	8	12	18
Aminoglycoside resistance	ANT(6)-Ia [Exiguobacterium sp. S3-2]	11	14	9
Aminoglycoside resistance	AAC(6')-Ie-APH(2")-Ia [Staphylococcus aureus]	1	3	3
Aminoglycoside resistance	aad(6) [Streptococcus oralis]	41	50	50
Bacitracin resistance protein	bacA [Escherichia coli str. K-12 substr. MG1655]	1	1	10
Beta-lactamase	CcrA beta-lactamase [Bacteroides fragilis]	229	133	75
Beta-lactamase	robA [Enterobacter cloacae]	1	1	4
Chloramphenicol resistance	cat [Vibrio anguillarum]	1	1	17
Efflux pump confering resistance	cmeA [Campylobacter jejuni subsp. doylei 269.97]	1	3	1
Efflux pump confering resistance	cmeB [Campylobacter jejuni subsp. doylei 269.97]	2	7	3
Efflux pump confering resistance	acrB [Enterobacter cloacae]	6	5	25
Efflux pump confering resistance	emrD [Escherichia coli B185]	4	3	12
Efflux pump confering resistance	cpxA [Escherichia coli O157:H7 str. Sakai]	6	4	17
Efflux pump confering resistance	cpxR [Escherichia coli O157:H7 str. Sakai]	1	1	7
Efflux pump confering resistance	gadW [Escherichia coli O25b:H4]	1	1	6
Efflux pump confering resistance	acrE [Escherichia coli str. K-12 substr. MG1655]	2	4	12
Efflux pump confering resistance	acrF [Escherichia coli str. K-12 substr. MG1655]	8	6	31
Efflux pump confering resistance	acrS [Escherichia coli str. K-12 substr. MG1655]	1	1	5

Efflux pump confering resistance	adiY [Escherichia coli str. K-12 substr. MG1655]	1	1	6
Efflux pump confering resistance	emrB [Escherichia coli str. K-12 substr. MG1655]	6	5	18
Efflux pump confering resistance	emrR [Escherichia coli str. K-12 substr. MG1655]	2	1	6
Efflux pump confering resistance	evgS [Escherichia coli str. K-12 substr. MG1655]	3	2	9
Efflux pump confering resistance	gadE [Escherichia coli str. K-12 substr. MG1655]	1	1	3
Efflux pump confering resistance	mdtA [Escherichia coli str. K-12 substr. MG1655]	3	2	8
Efflux pump confering resistance	mdtB [Escherichia coli str. K-12 substr. MG1655]	8	8	28
Efflux pump confering resistance	mdtC [Escherichia coli str. K-12 substr. MG1655]	8	6	29
Efflux pump confering resistance	mdtD [Escherichia coli str. K-12 substr. MG1655]	4	2	10
Efflux pump confering resistance	mdtF [Escherichia coli str. K-12 substr. MG1655]	8	6	40
Efflux pump confering resistance	mdtH [Escherichia coli str. K-12 substr. MG1655]	2	2	8
Efflux pump confering resistance	mdtL [Escherichia coli str. K-12 substr. MG1655]	3	2	13
Efflux pump confering resistance	mdtM [Escherichia coli str. K-12 substr. MG1655]	4	4	16
Efflux pump confering resistance	mfd [Escherichia coli str. K-12 substr. MG1655]	11	9	28
Efflux pump confering resistance	msbA [Escherichia coli str. K-12 substr. MG1655]	4	3	15
Efflux pump confering resistance	YojI [Escherichia coli str. K-12 substr. MG1655]	5	4	10
Efflux pump confering resistance	acrD [Escherichia coli str. K-12 substr. W3110]	9	9	34
Efflux pump confering resistance	baeR [Escherichia coli str. K-12 substr. W3110]	1	1	6
Efflux pump confering resistance	baeS [Escherichia coli str. K-12 substr. W3110]	3	3	16
Efflux pump confering resistance	CRP [Escherichia coli str. K-12 substr. W3110]	3	1	9
Efflux pump confering resistance	emrA [Escherichia coli str. K-12 substr. W3110]	1	3	11
Efflux pump confering resistance	mdtE [Escherichia coli str. K-12 substr. W3110]	2	4	18
Efflux pump confering resistance	mdtG [Escherichia coli str. K-12 substr. W3110]	2	2	9
Efflux pump confering resistance	mdtN [Escherichia coli str. K-12 substr. W3110]	4	4	12
Efflux pump confering resistance	mdtO [Escherichia coli str. K-12 substr. W3110]	5	5	28

Efflux pump confering resistance	mdtP [Escherichia coli str. K-12 substr. W3110]	7	5	24
Efflux pump confering resistance	emrK [Escherichia coli]	1	1	4
Efflux pump confering resistance	mdfA [Escherichia coli]	4	2	13
Efflux pump confering resistance	tolC [Escherichia coli]	4	3	18
Efflux pump confering resistance	acrA [Klebsiella pneumoniae subsp. pneumoniae HS11286]	1	1	5
Efflux pump confering resistance	sav1866 [Staphylococcus aureus subsp. aureus COL]	2	3	1
Fluoroquinolone resistance	patA [Escherichia coli str. K-12 substr. MG1655]	4	6	20
Glycopeptide antibiotics resistance	vanV [Enterococcus faecalis V583]	10	11	17
Glycopeptide antibiotics resistance	vanG [Enterococcus faecalis]	1	2	3
Glycopeptide antibiotics resistance	vanRG [Enterococcus faecalis]	1	4	3
Glycopeptide antibiotics resistance	vanSG [Enterococcus faecalis]	4	7	9
Glycopeptide antibiotics resistance	vanYG1 [Enterococcus faecalis]	1	1	2
Glycopeptide antibiotics resistance	vanRD [Enterococcus faecium]	1	2	1
Glycopeptide antibiotics resistance	vanSC [Enterococcus gallinarum]	3	4	3
Macrolide-Lincosamide-StreptograminB resistance	ErmG [Bacteroides thetaiotaomicron]	2	9	27
Macrolide-Lincosamide-StreptograminB resistance	ErmB [Enterococcus faecalis]	2	2	2
Macrolide-Lincosamide-StreptograminB resistance	ErmB [Enterococcus faecium]	2	2	3
Macrolide-Lincosamide-StreptograminB resistance	vatE [Enterococcus faecium]	130	27	12
Macrolide-Lincosamide-StreptograminB resistance	mefA [Exiguobacterium sp. S3-2]	5	1	4
Macrolide-Lincosamide-StreptograminB resistance	ErmG [Lysinibacillus sphaericus]	13	1	3
Macrolide-Lincosamide-StreptograminB resistance	ErmB [Plasmid pIP1527]	1	1	1
Macrolide-Lincosamide-StreptograminB resistance	lnuC [Streptococcus agalactiae]	258	279	281
Macrolide-Lincosamide-StreptograminB resistance	ErmB [Streptococcus pyogenes]	1	1	2
Polymyxin resistance	arnA [Escherichia coli str. K-12 substr. MG1655]	5	4	14
Polymyxin resistance	PmrE [Escherichia coli str. K-12 substr. MG1655]	8	6	16

Polymyxin resistance	PmrF [Escherichia coli str. K-12 substr. MG1655]	2	2	8
Polymyxin resistance	PmrB [Escherichia coli str. K-12 substr. W3110]	4	5	12
Polymyxin resistance	PmrC [Escherichia coli str. K-12 substr. W3110]	5	2	19
Streptothricin resistance	sat-4 [Campylobacter coli]	45	37	41
Tetracycline resistance	tetQ [Bacteroides fragilis]	403	205	135
Tetracycline resistance	tetW [Bifidobacterium longum]	1611	2150	2082
Tetracycline resistance	tet44 [Campylobacter fetus subsp. fetus]	25	73	20
Tetracycline resistance	tetO [Campylobacter jejuni]	61	42	107
Tetracycline resistance	tet32 [Clostridiaceae bacterium K10]	60	28	75
Tetracycline resistance	tetM [Clostridium difficile 630]	7	1	3
Tetracycline resistance	tetS [Listeria monocytogenes]	2	1	2
Tetracycline resistance	tet40 [Streptococcus suis]	65	80	51

Supplementary Table 5. Core resistome of chickens from group D with relative abundances of ARGs in individual samples. Relative abundance was adjusted to abundance per 10 million sequences.

Group	Gene	Relative abundance		
		D1	D2	D3
Aminocoumarin resistance	aminocoumarin resistant alaS [Escherichia coli str. K-12 substr. MG1655]	5	7	3
Aminoglycoside resistance	APH(2")-Ig [Campylobacter coli CVM N29710]	18	4	8
Aminoglycoside resistance	APH(3')-IIIa [Campylobacter coli CVM N29710]	91	92	61
Aminoglycoside resistance	ANT(6)-Ib [Campylobacter jejuni]	74	1	27
Aminoglycoside resistance	APH(2")-IVa [Enterococcus casseliflavus]	2	3	1
Aminoglycoside resistance	AAC(6')-Ip [Escherichia coli]	20	13	10
Aminoglycoside resistance	APH(2")-IIa [Escherichia coli]	9	13	4
Aminoglycoside resistance	ANT(6)-Ia [Exiguobacterium sp. S3-2]	5	10	3
Aminoglycoside resistance	APH(3')-Ia [Serratia marcescens]	1	1	1
Aminoglycoside resistance	aad(6) [Streptococcus oralis]	38	49	18
Bacitracin resistance protein	bacA [Escherichia coli str. K-12 substr. MG1655]	1	2	1
Beta-lactamase	CcrA beta-lactamase [Bacteroides fragilis]	250	218	117
Chloramphenicol resistance	cat [Vibrio anguillarum]	2	23	2
Efflux pump confering resistance	acrB [Enterobacter cloacae]	3	3	2
Efflux pump confering resistance	emrD [Escherichia coli B185]	2	2	2
Efflux pump confering resistance	cpxA [Escherichia coli O157:H7 str. Sakai]	2	7	2
Efflux pump confering resistance	acrE [Escherichia coli str. K-12 substr. MG1655]	2	3	1
Efflux pump confering resistance	acrF [Escherichia coli str. K-12 substr. MG1655]	4	8	2
Efflux pump confering resistance	adiY [Escherichia coli str. K-12 substr. MG1655]	1	1	1
Efflux pump confering resistance	emrB [Escherichia coli str. K-12 substr. MG1655]	2	4	2
Efflux pump confering resistance	mdtA [Escherichia coli str. K-12 substr. MG1655]	1	2	1
Efflux pump confering resistance	mdtB [Escherichia coli str. K-12 substr. MG1655]	3	5	2
Efflux pump confering resistance	mdtC [Escherichia coli str. K-12 substr. MG1655]	3	8	2
Efflux pump confering resistance	mdtF [Escherichia coli str. K-12 substr. MG1655]	5	8	3
Efflux pump confering resistance	mdtL [Escherichia coli str. K-12 substr. MG1655]	2	4	2
Efflux pump confering resistance	mdtM [Escherichia coli str. K-12 substr. MG1655]	1	4	1

Efflux pump confering resistance	mfd [Escherichia coli str. K-12 substr. MG1655]	3	5	3
Efflux pump confering resistance	YojI [Escherichia coli str. K-12 substr. MG1655]	2	2	1
Efflux pump confering resistance	acrD [Escherichia coli str. K-12 substr. W3110]	3	8	2
Efflux pump confering resistance	baeR [Escherichia coli str. K-12 substr. W3110]	1	1	1
Efflux pump confering resistance	baeS [Escherichia coli str. K-12 substr. W3110]	2	4	1
Efflux pump confering resistance	emrA [Escherichia coli str. K-12 substr. W3110]	2	2	1
Efflux pump confering resistance	mdtE [Escherichia coli str. K-12 substr. W3110]	1	5	1
Efflux pump confering resistance	mdtG [Escherichia coli str. K-12 substr. W3110]	1	2	1
Efflux pump confering resistance	mdtN [Escherichia coli str. K-12 substr. W3110]	2	3	1
Efflux pump confering resistance	mdtO [Escherichia coli str. K-12 substr. W3110]	3	4	2
Efflux pump confering resistance	mdtP [Escherichia coli str. K-12 substr. W3110]	3	5	2
Efflux pump confering resistance	mdfA [Escherichia coli]	1	2	2
Efflux pump confering resistance	tolC [Escherichia coli]	2	4	2
Efflux pump confering resistance	sav1866 [Staphylococcus aureus subsp. aureus COL]	2	2	2
Fluoroquinolone resistance	patA [Escherichia coli str. K-12 substr. MG1655]	1	4	2
Glycopeptide antibiotics resistance	vanV [Enterococcus faecalis V583]	16	15	10
Glycopeptide antibiotics resistance	vanG [Enterococcus faecalis]	2	1	2
Glycopeptide antibiotics resistance	vanRG [Enterococcus faecalis]	1	1	2
Glycopeptide antibiotics resistance	vanSG [Enterococcus faecalis]	2	3	10
Glycopeptide antibiotics resistance	vanW [Enterococcus faecalis]	1	1	1
Glycopeptide antibiotics resistance	vanYG1 [Enterococcus faecalis]	1	1	2
Glycopeptide antibiotics resistance	vanRD [Enterococcus faecium]	1	1	1
Glycopeptide antibiotics resistance	vanSC [Enterococcus gallinarum]	3	2	7
Macrolide-Lincosamide-StreptograminB resistance	ErmB [Enterococcus faecalis]	3	3	2
Macrolide-Lincosamide-StreptograminB resistance	vatE [Enterococcus faecium]	12	23	10

Macrolide-Lincosamide-StreptograminB resistance	ErmB [Lactobacillus fermentum]	1	6	10
Macrolide-Lincosamide-StreptograminB resistance	ErmB [Plasmid pAM77]	1	1	11
Macrolide-Lincosamide-StreptograminB resistance	ErmB [Plasmid pIP1527]	1	1	2
Macrolide-Lincosamide-StreptograminB resistance	lnuC [Streptococcus agalactiae]	207	355	88
Macrolide-Lincosamide-StreptograminB resistance	ErmB [Streptococcus pyogenes]	2	2	2
Polymyxin resistance	arnA [Escherichia coli str. K-12 substr. MG1655]	2	3	3
Polymyxin resistance	PmrE [Escherichia coli str. K-12 substr. MG1655]	10	6	8
Polymyxin resistance	PmrB [Escherichia coli str. K-12 substr. W3110]	3	5	2
Polymyxin resistance	PmrC [Escherichia coli str. K-12 substr. W3110]	1	5	1
Streptothricin resistance	sat-4 [Campylobacter coli]	32	49	11
Tetracycline resistance	tetQ [Bacteroides fragilis]	385	386	89
Tetracycline resistance	tetW [Bifidobacterium longum]	1468	1835	1764
Tetracycline resistance	tet44 [Campylobacter fetus subsp. fetus]	45	37	27
Tetracycline resistance	tetO [Campylobacter jejuni]	76	206	15
Tetracycline resistance	tet32 [Clostridiaceae bacterium K10]	79	158	26
Tetracycline resistance	tetM [Clostridium difficile 630]	6	5	1
Tetracycline resistance	tetS [Listeria monocytogenes]	2	2	2
Tetracycline resistance	tet40 [Streptococcus suis]	68	49	128
